# Supplementary material for: ImmunoPET imaging of Trop2 in patients with solid tumours
Source: EMBO Mol Med. 2024 Apr 2;16(5):6. doi: 10.1038/s44321-024-00059-5 (PMC11099157; doi:10.1038/s44321-024-00059-5)
Supplement: Supplementary file 1 — Appendix [file 44321_2024_59_MOESM1_ESM.pdf]

ImmunoPET imaging of Trop2 in patients with solid tumours

Wei Huang[1,#], You Zhang[1,#], Min Cao[2,#], Yanfei Wu[3], Feng Jiao[4], Zhaohui Chu[5], Xinyuan Zhou[1], Lianghua Li[1], Dongsheng Xu[1], Xinbing Pan[1], Yihui Guan[3], Gang Huang[1], Jianjun Liu[1,*], Fang Xie[3,*], Weijun Wei[1,*]

[1]Department of Nuclear Medicine, Institute of Clinical Nuclear Medicine, Renji Hospital, School of Medicine, Shanghai Jiao Tong University, 1630 Dongfang Rd, Shanghai 200127, China.

[2] Department of Thoracic Surgery, Renji Hospital, Shanghai Jiao Tong University School of Medicine, Shanghai 200217, China.

[3] Department of Nuclear Medicine & PET Center, Huashan Hospital, Fudan University, Shanghai 200040, China.

[4] Department of Oncology, State Key Laboratory of Systems Medicine for Cancer, Shanghai Cancer Institute, Renji Hospital, School of Medicine, Shanghai Jiao Tong University, Shanghai 200127, China.

[5] Department of Oncology, Huashan Hospital, Fudan University, Shanghai 20017, China.

[#] The authors contributed equally to the work.

[*] Corresponding author:

Prof. Weijun Wei

Department of Nuclear Medicine, Renji Hospital, School of Medicine, Shanghai Jiao Tong University, 1630 Dongfang Rd, Shanghai 200127, China. E-mail: wwei@shsmu.edu.cn.

Prof. Jianjun Liu

Department of Nuclear Medicine, Renji Hospital, School of Medicine, Shanghai Jiao Tong University, 1630 Dongfang Rd, Shanghai 200127, China. E-mail: ljjsh@sjtu.edu.cn.

Prof. Fang Xie

Department of Nuclear Medicine & PET Center, Huashan Hospital, Fudan University, Shanghai 200040, China. E-mail: fangxie@fudan.edu.cn.

Appendix Figure and Figure legends

Table of Contents

Appendix Figure S1: Page 3

Appendix Figure S2: Page 4

Appendix Figure S3: Page 5

Appendix Figure S4: Page 6

Appendix Figure S5: Page 7

Appendix Figure S6: Page 8

Appendix Figure S7: Page 9

Appendix Figure S8: Page 10

Appendix Figure S9: Page 11

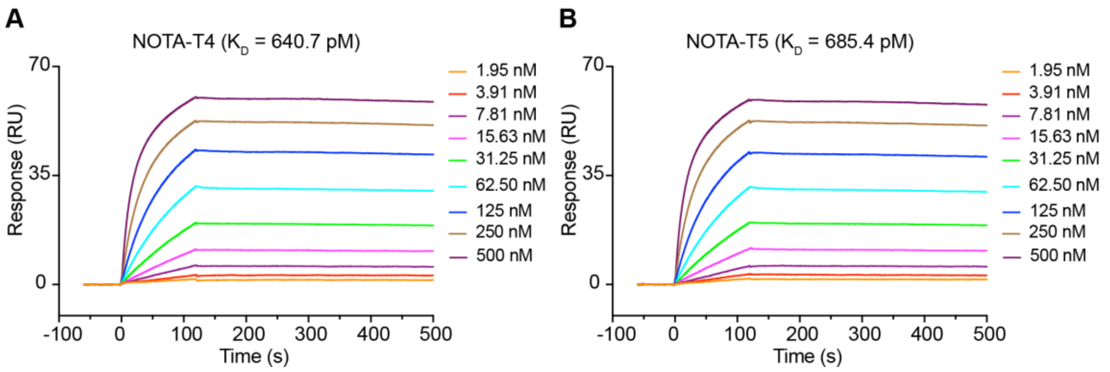

Appendix Figure S1. Surface plasmon resonance (SPR) analysis revealed the binding affinity of NOTA-T4 (A) and NOTA-T5 (B) interacting with recombinant human Trop2 protein.

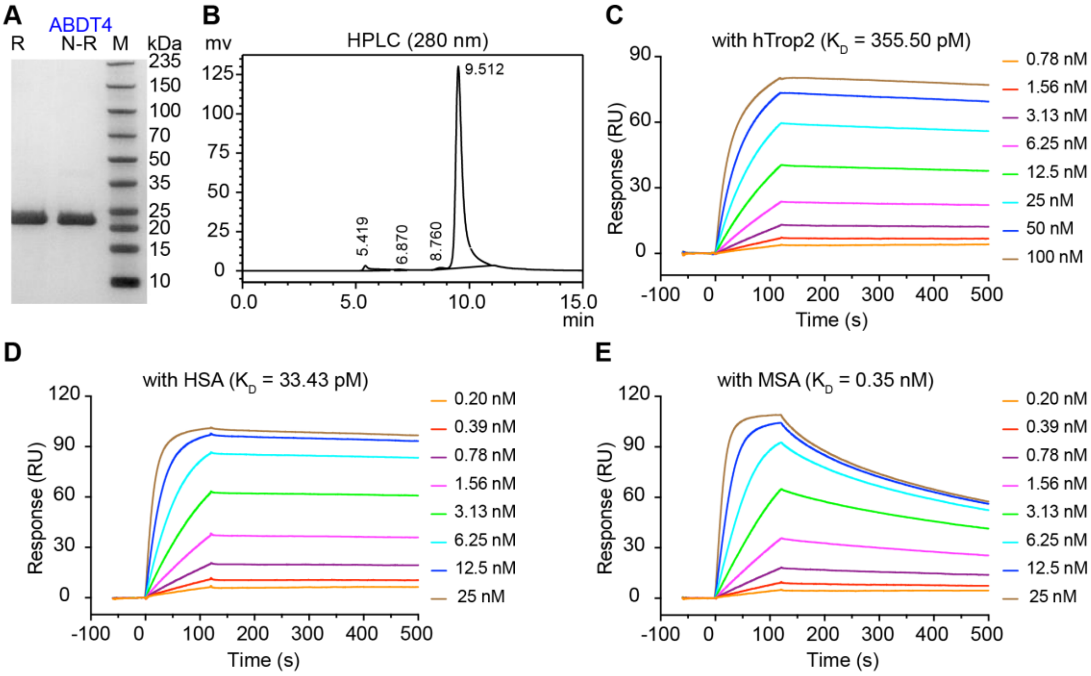

Appendix Figure S2. Expression and Characterization of ABDT4. (A) SDS-PAGE tested the purity of ABDT4. R: reducing conditions; N-R: non-reducing conditions. (B) Characterisation of ABDT4 by HPLC. (C–E) The binding affinities of ABDT4 to immobilised recombinant human Trop2 protein (C), human serum albumin (D), and murine serum albumin (E) were assayed by SPR, respectively. Source data are available online for Figure S2A.

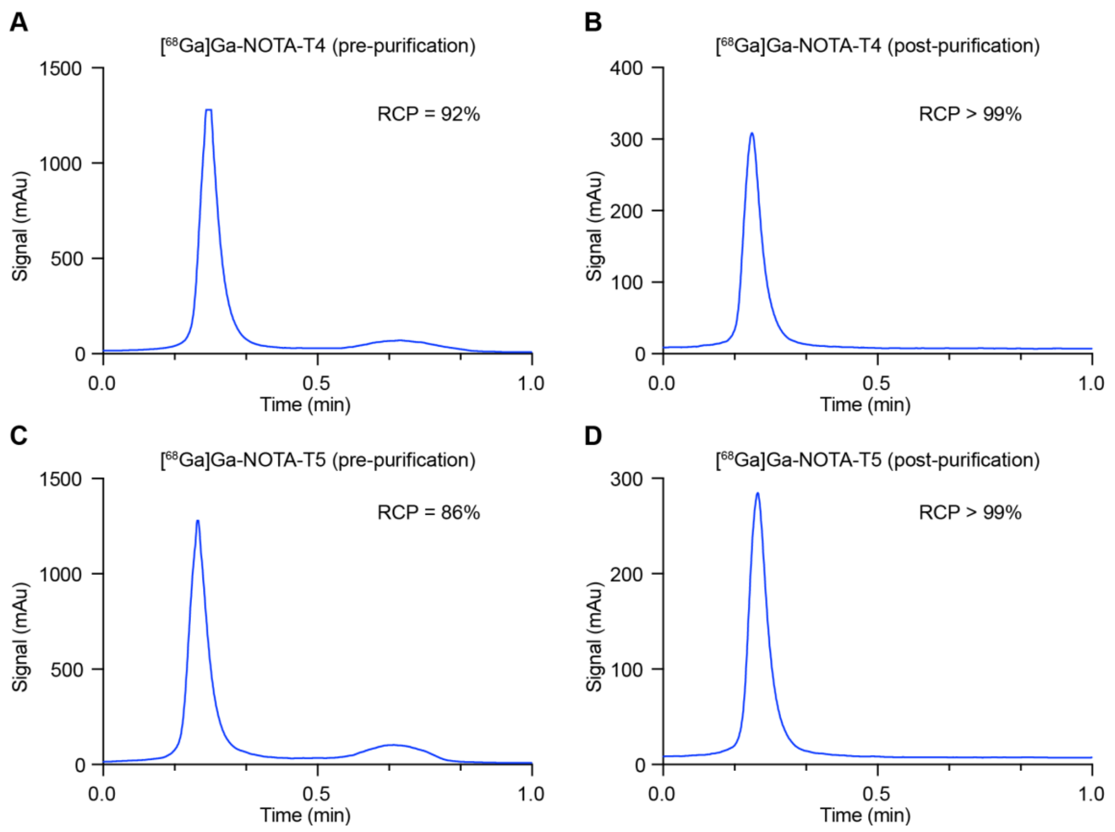

Appendix Figure S3. Assessment of radiochemical purity (RCP) of the tracers. The pre-purification (A) and post-purification (B) RCP of [^{68}Ga]Ga-NOTA-T4 were 92% and >99%, respectively. The pre-purification (C) and post-purification (D) RCP of [^{68}Ga]Ga-NOTA-T5 were 86% and >99%, respectively.

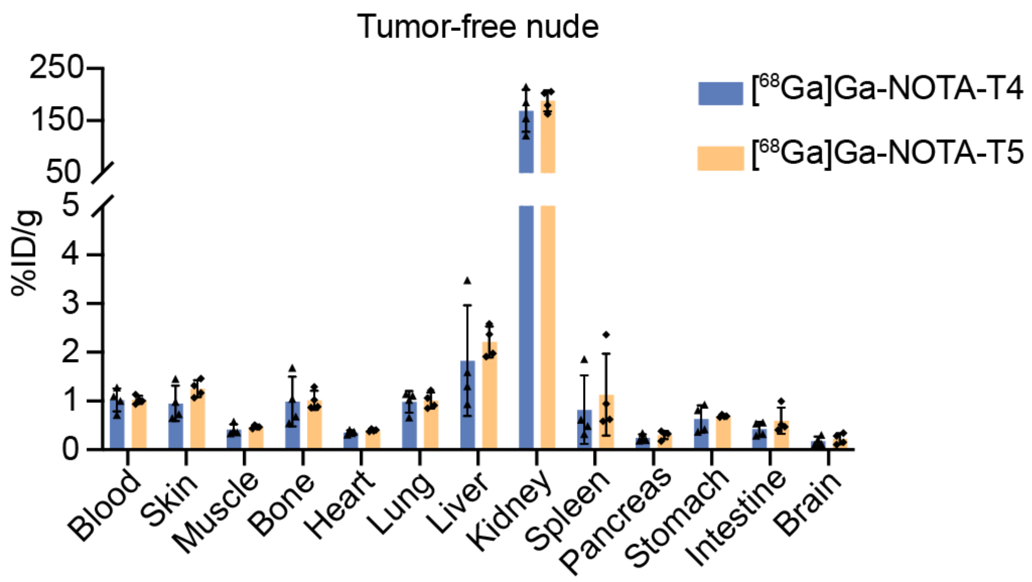

Appendix Figure S4. Distribution of radioactivity in each organ/tissue of tumour-free mice models after [^{68}Ga]Ga-NOTA-T4 (n = 4) and [^{68}Ga]Ga-NOTA-T5 (n = 4) immunoPET imaging.

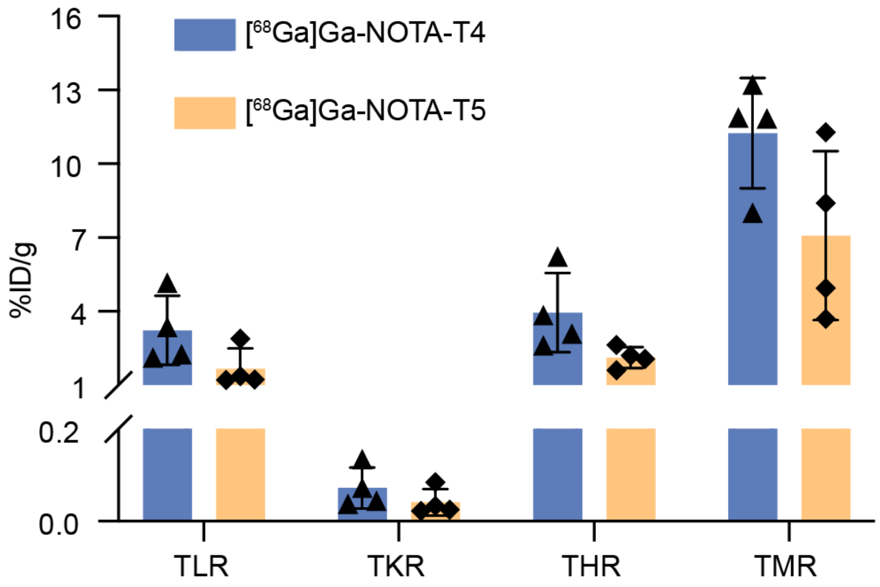

Appendix Figure S5. Comparison of tumor-to-organ ratios at [^{68}Ga]Ga-NOTA-T4 and [^{68}Ga]Ga-NOTA-T5. TLR: tumor-to-liver; TKR: tumor-to-kidney; THR: tumor-to-heart; TMR: tumor-to-muscle. *T*-test (Mean ratio ± SD, n = 4 for each group).

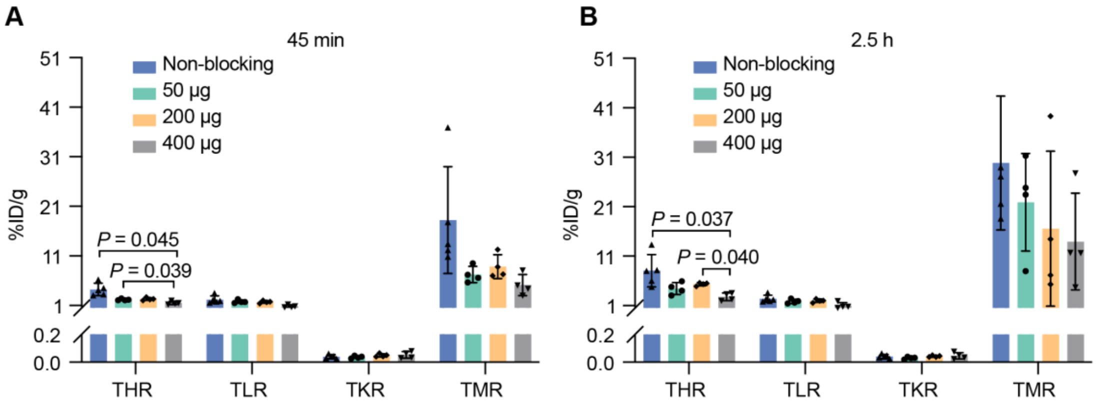

Appendix Figure S6. Comparison of tumour-to-organ ratios at two time points (45 min, A; 2.5 h, B) between the unblocking and the three blocking groups. THR: tumor-to-heart; TLR: tumor-to-liver; TKR: tumor-to-kidney; TMR: tumor-to-muscle. Two-way ANOVA.

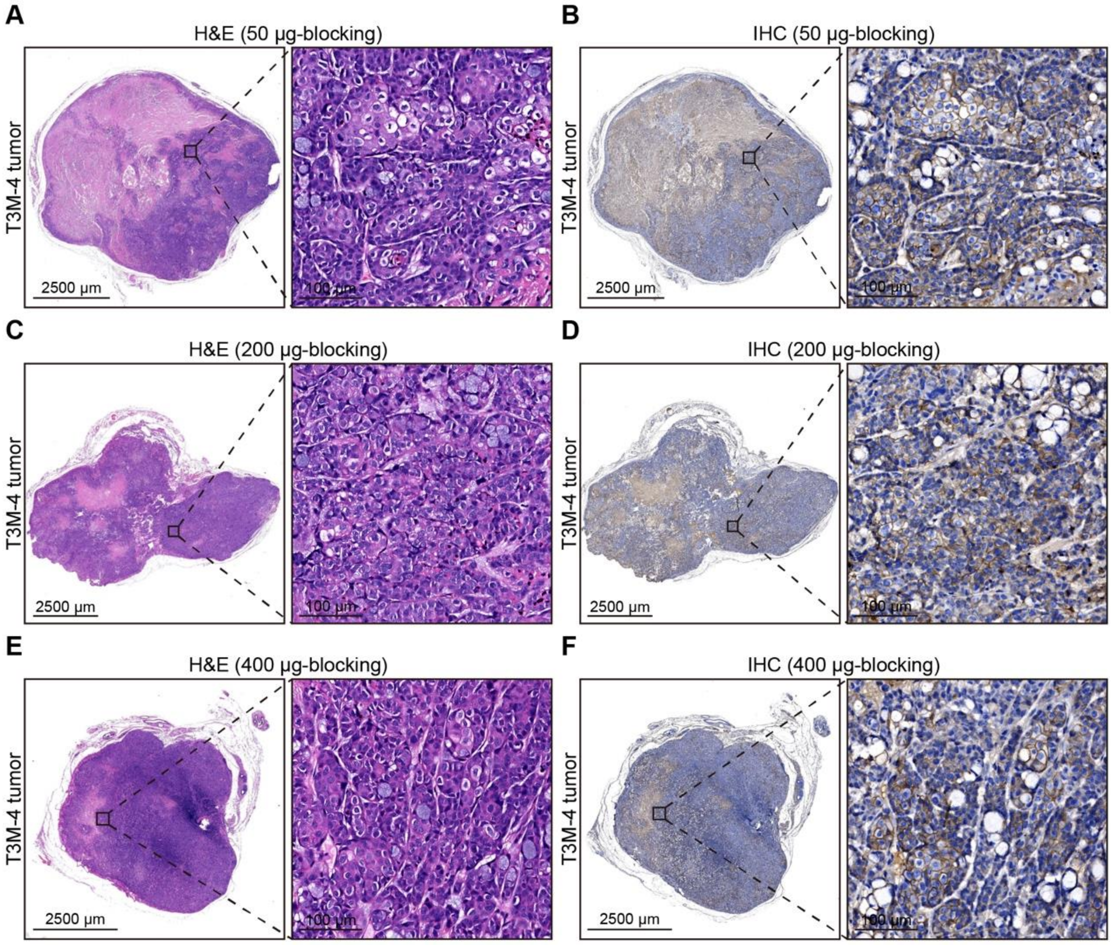

Appendix Figure S7. Hematoxylin-eosin (H&E) and immunohistochemistry (IHC) staining results of tumour tissues from different blocked groups. (A, C, and E) H&E staining of tumours from the blocking dose of 50 μg (A), 200 μg (C), and 400 μg (E). Scale bar: 2500 μm and 100 μm. (B, D, and F) IHC staining of Trop2 expression in the 50 μg (B), 200 μg (D), and 400 μg (F) blocking groups. Scale bar: 2500 μm and 100 μm.

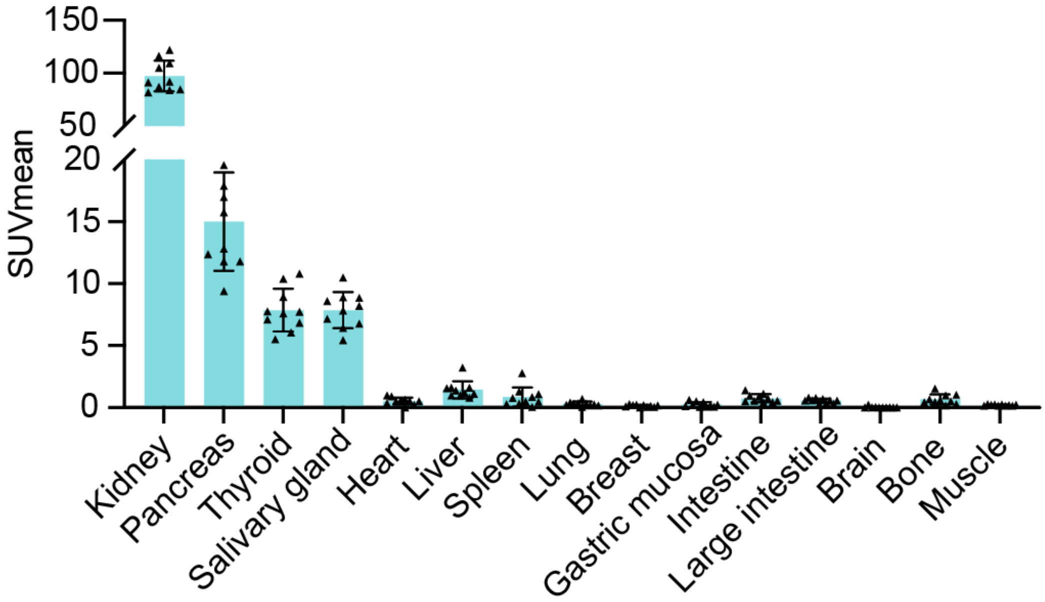

Appendix Figure S8. Radioactivity uptake values in normal tissues from 10 patients with malignant tumours regarding SUVmean.

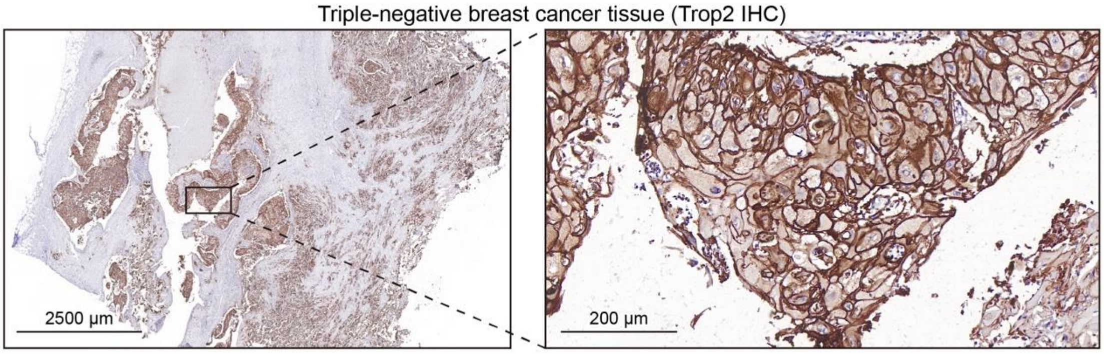

Appendix Figure S9. Trop2 IHC staining of triple-negative breast cancer tissue. IHC staining result showed Trop2 high expression. Scale bar: 2500 μm and 200 μm.
