# Supplementary figures and images for: ImmunoPET imaging of Trop2 in patients with solid tumours
Source: EMBO Mol Med. 2024 Apr 2;16(5):6. doi: 10.1038/s44321-024-00059-5 (PMC11099157; doi:10.1038/s44321-024-00059-5)

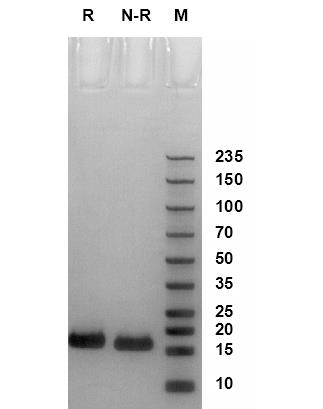

Supplement: Supplementary file 3 — Source data Fig. 1 [file 44321_2024_59_MOESM3_ESM.zip › Figure 1/1A/SDS-PAGE T4.jpg]

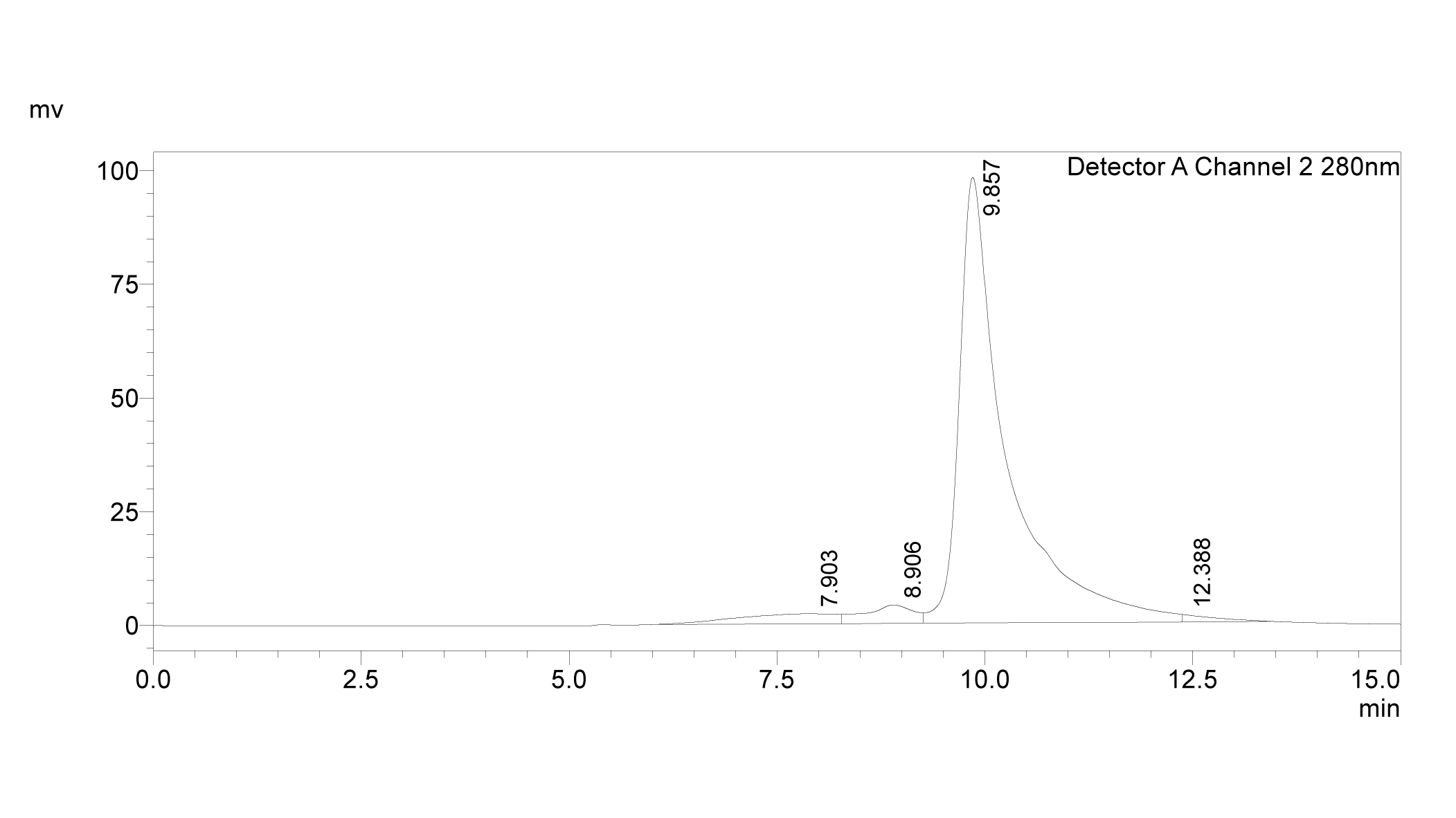

Supplement: Supplementary file 3 — Source data Fig. 1 [file 44321_2024_59_MOESM3_ESM.zip › Figure 1/1B/HPLC T4.tif]

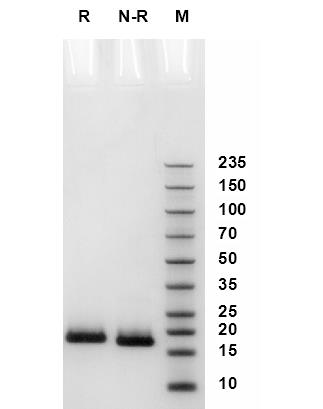

Supplement: Supplementary file 3 — Source data Fig. 1 [file 44321_2024_59_MOESM3_ESM.zip › Figure 1/1D/SDS-PAGE T5.jpg]

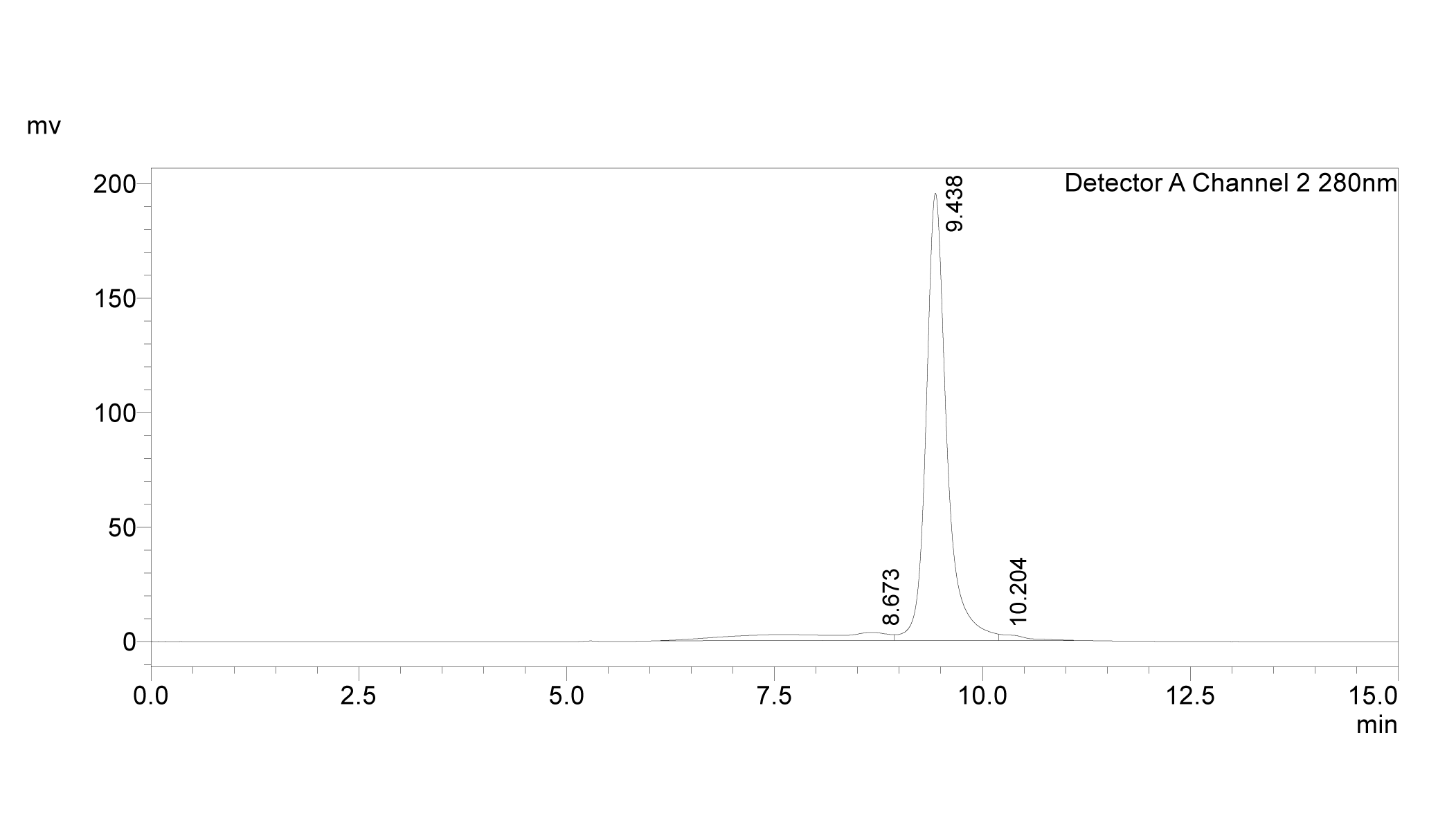

Supplement: Supplementary file 3 — Source data Fig. 1 [file 44321_2024_59_MOESM3_ESM.zip › Figure 1/1E/HPLC T5 .tif]

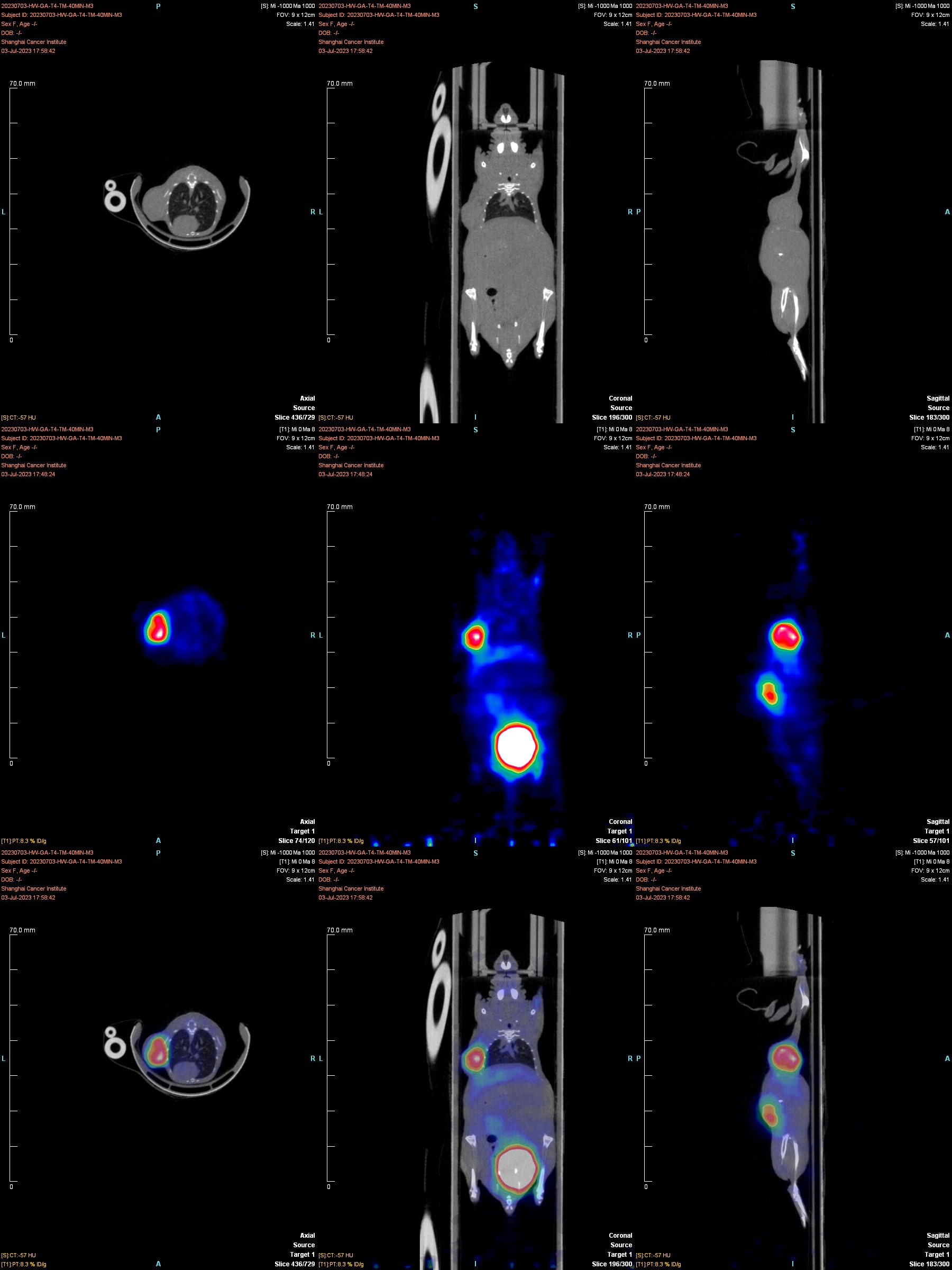

Supplement: Supplementary file 4 — Source data Fig. 2 [file 44321_2024_59_MOESM4_ESM.zip › Figure 2/2A/T4-0-8.jpg]

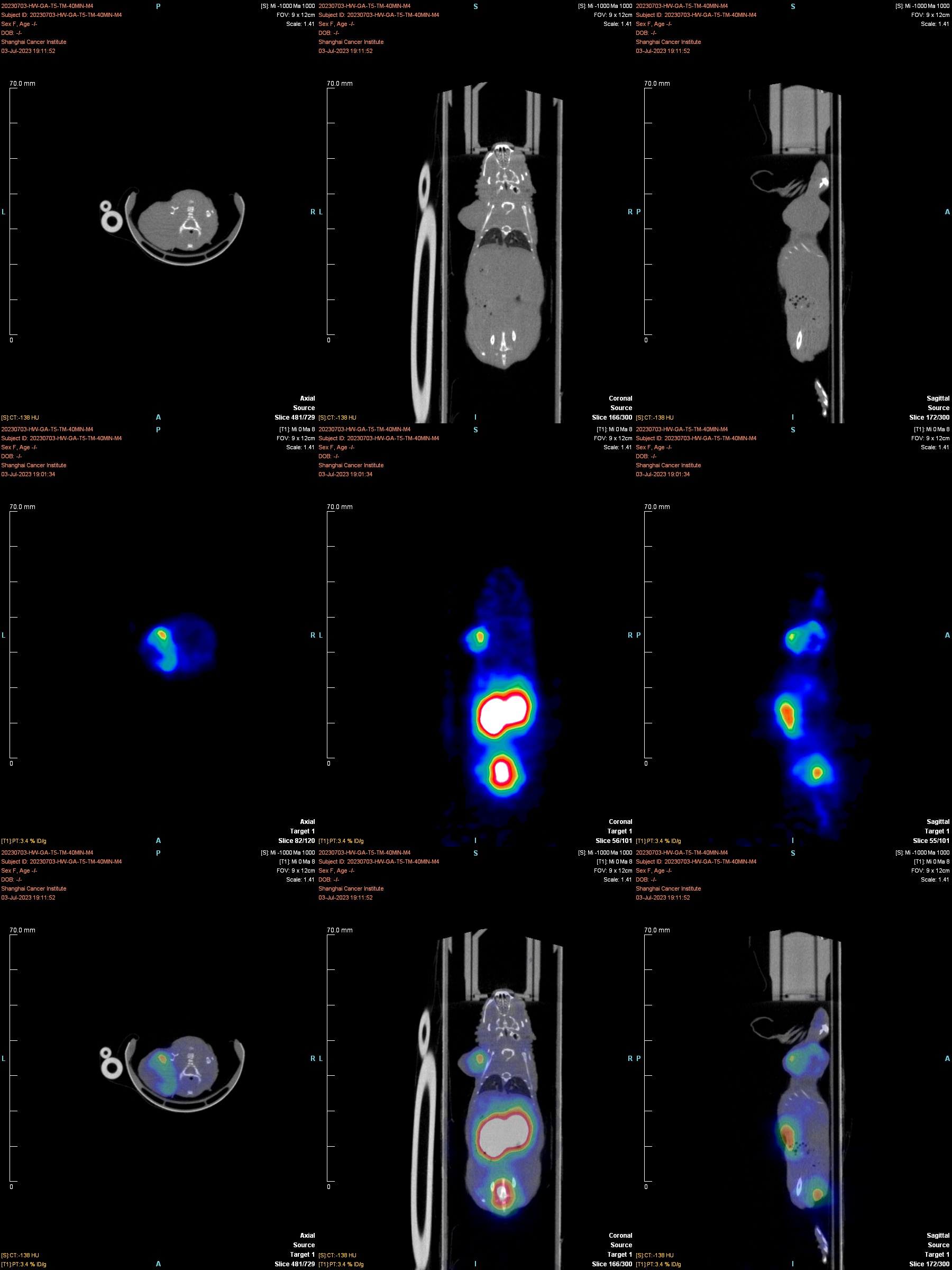

Supplement: Supplementary file 4 — Source data Fig. 2 [file 44321_2024_59_MOESM4_ESM.zip › Figure 2/2D/T5-0-8.jpg]

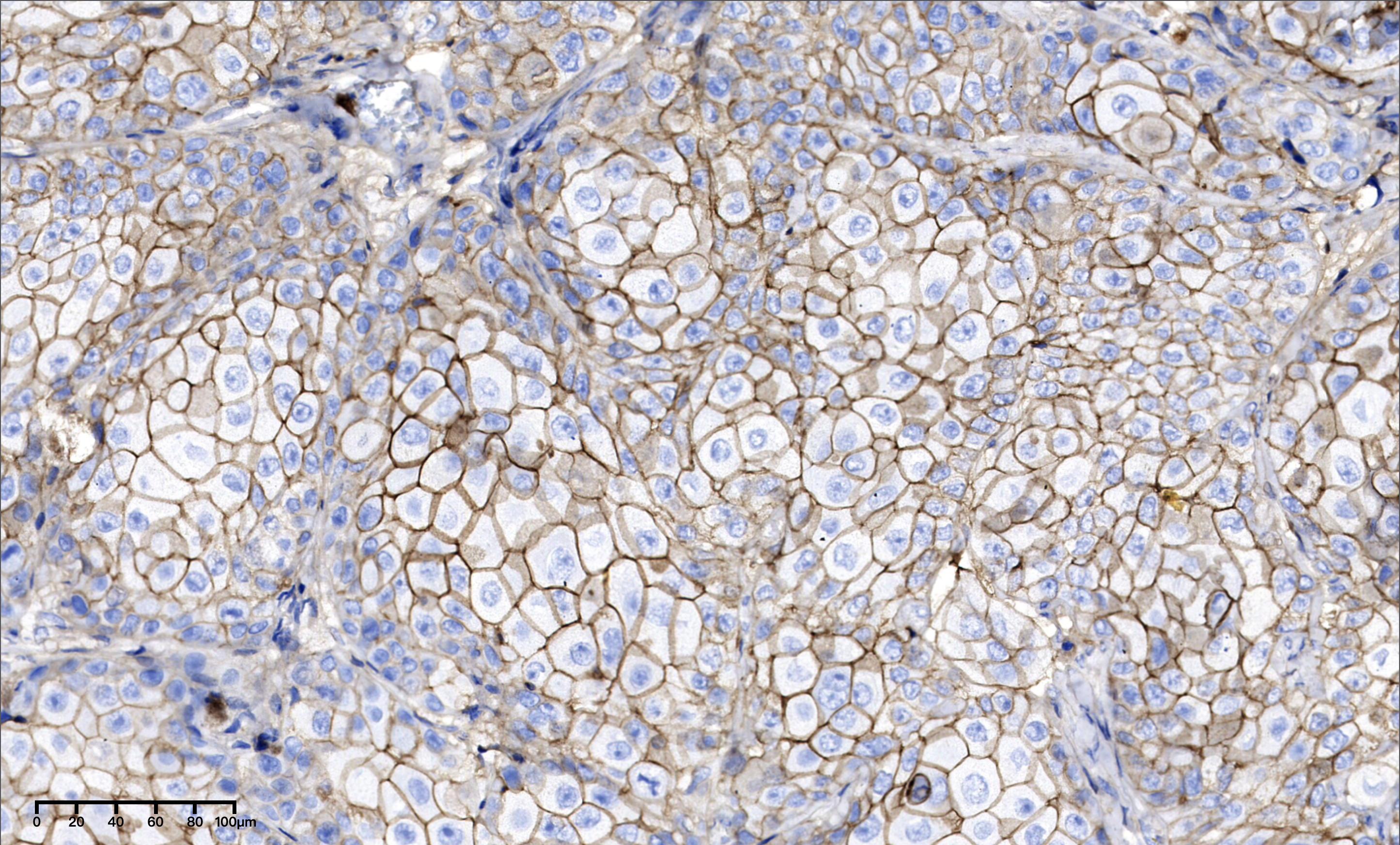

Supplement: Supplementary file 4 — Source data Fig. 2 [file 44321_2024_59_MOESM4_ESM.zip › Figure 2/2G/T4-T3M-4 TROP2 100.png]

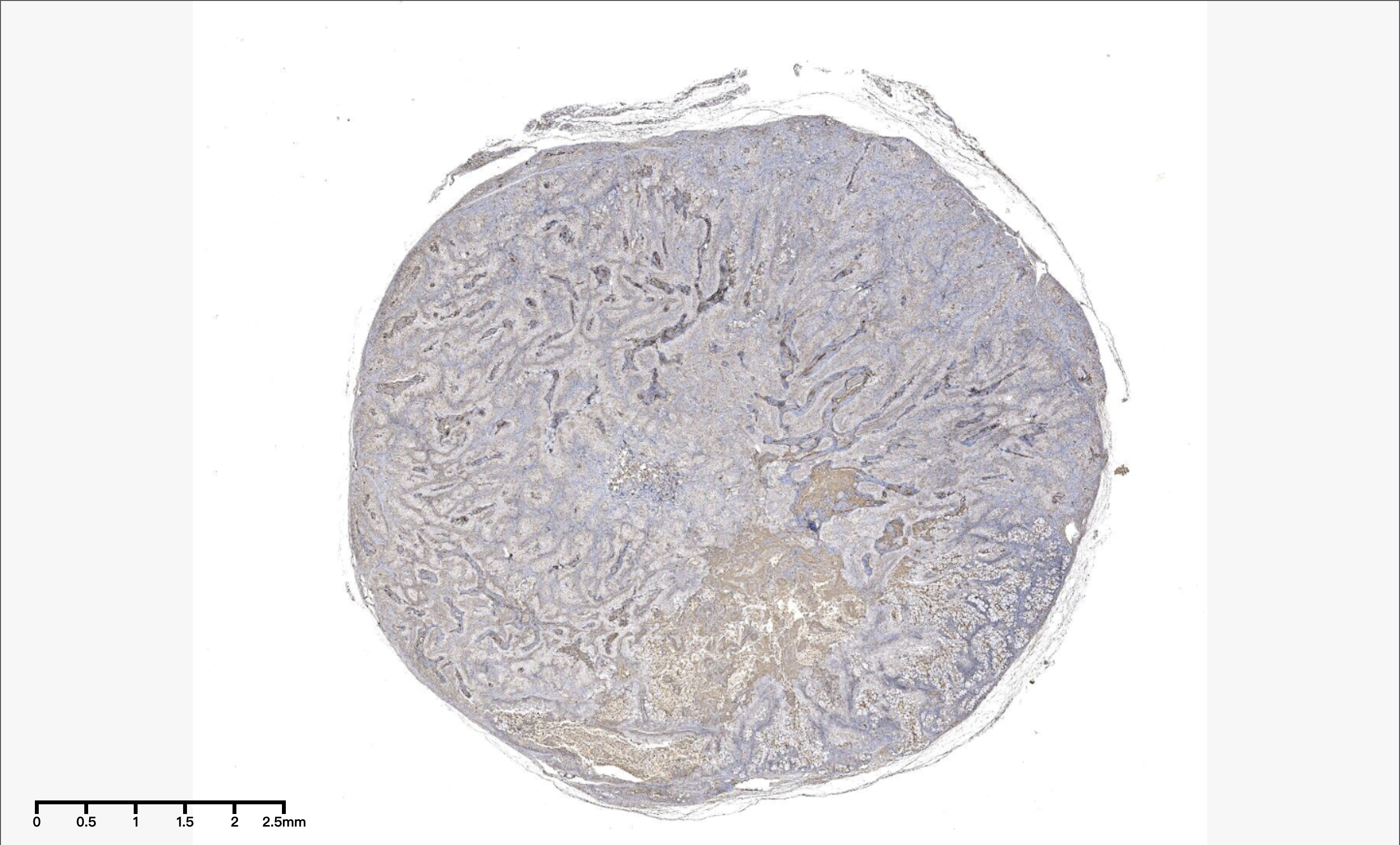

Supplement: Supplementary file 4 — Source data Fig. 2 [file 44321_2024_59_MOESM4_ESM.zip › Figure 2/2G/T4-T3M-4 TROP2 2500.png]

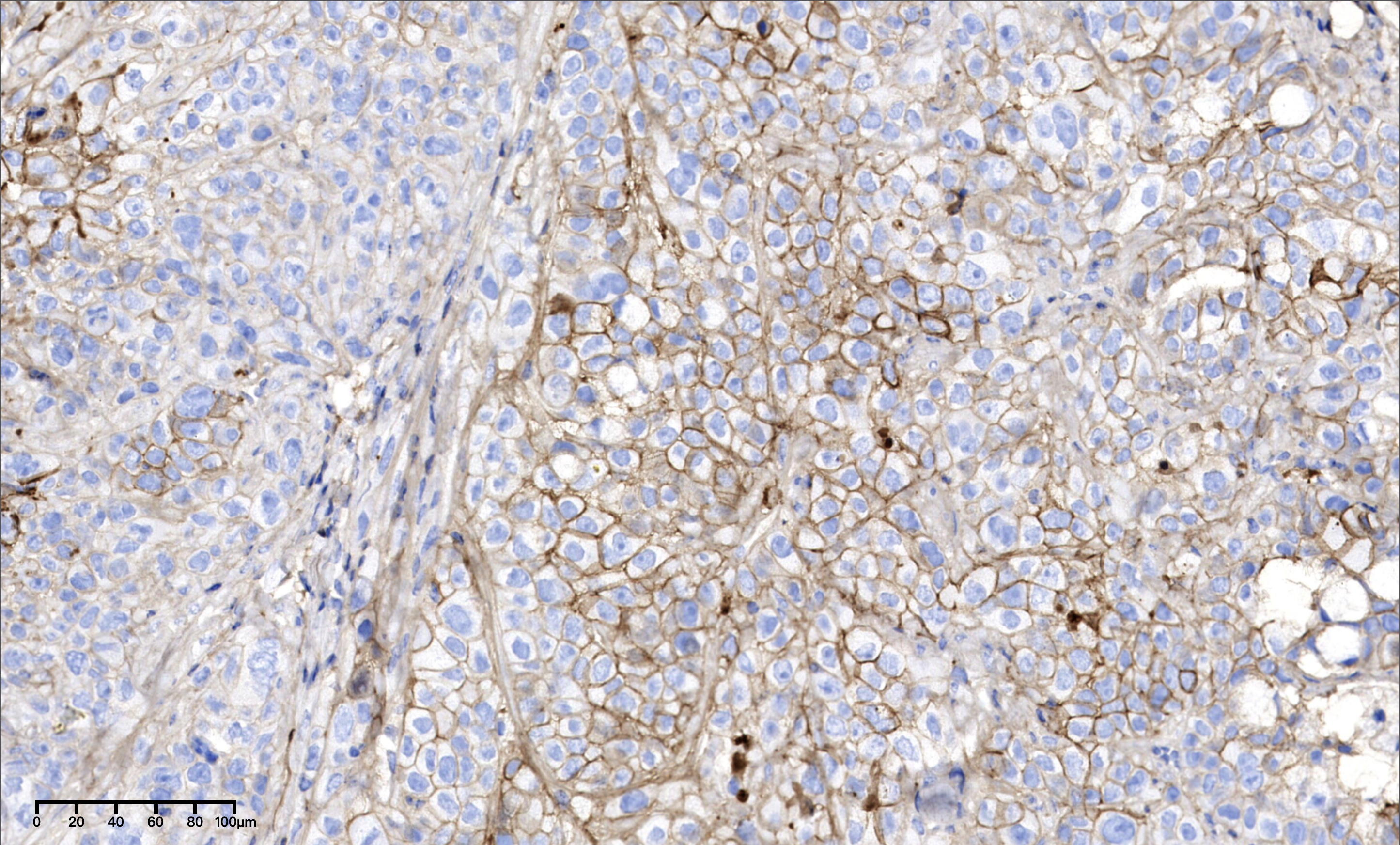

Supplement: Supplementary file 4 — Source data Fig. 2 [file 44321_2024_59_MOESM4_ESM.zip › Figure 2/2H/T5-T3M-4 TROP2 100.png]

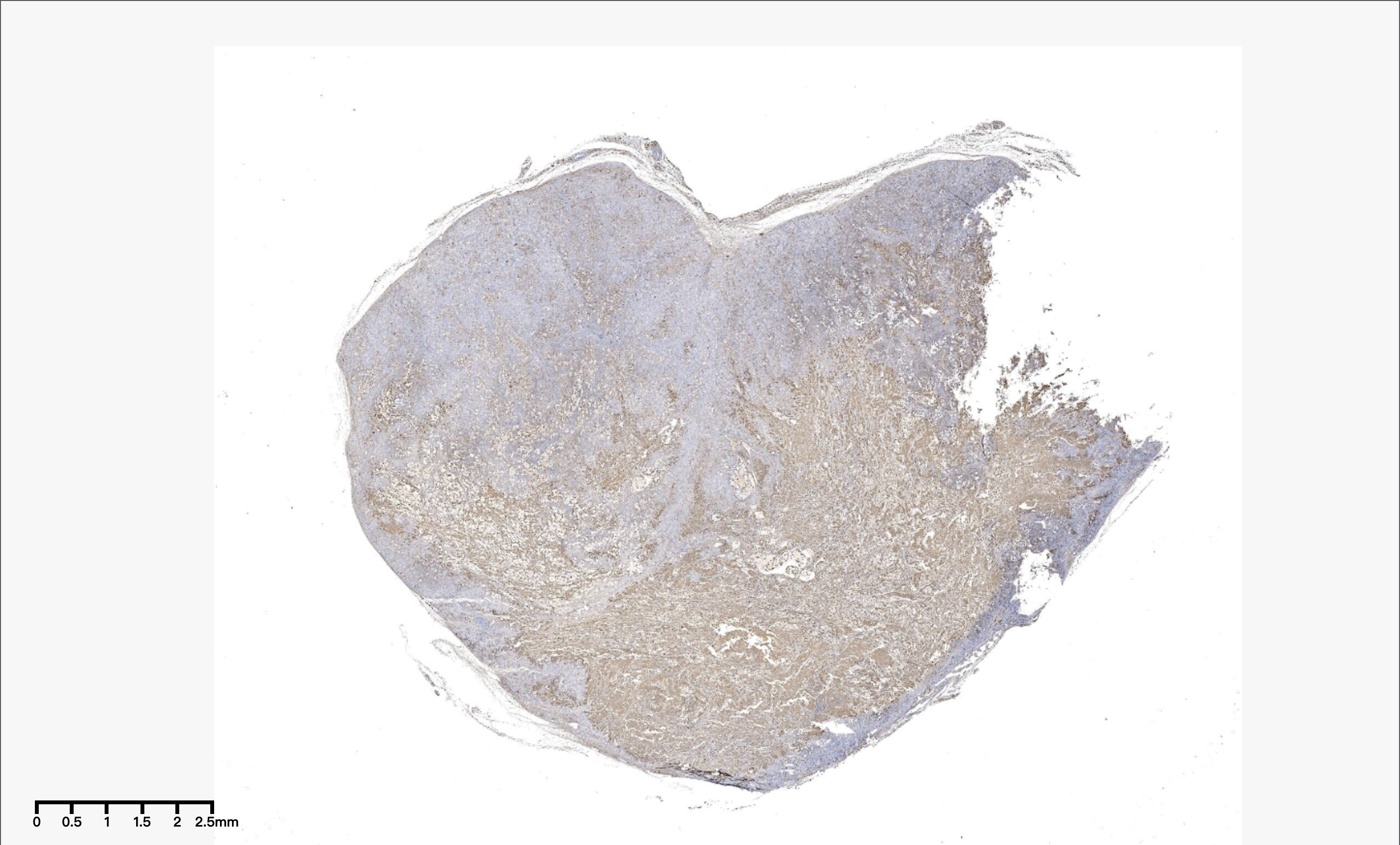

Supplement: Supplementary file 4 — Source data Fig. 2 [file 44321_2024_59_MOESM4_ESM.zip › Figure 2/2H/T5-T3M-4 TROP2 2500.png]

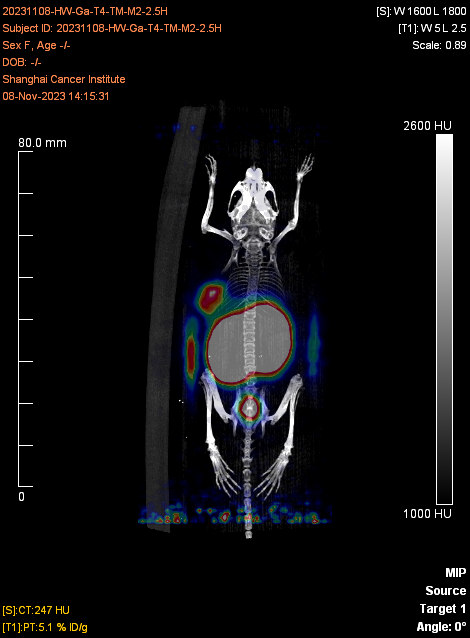

Supplement: Supplementary file 5 — Source data Fig. 3 [file 44321_2024_59_MOESM5_ESM.zip › Figure 3/3A/T4-2.5H-0-5-MIP.png]

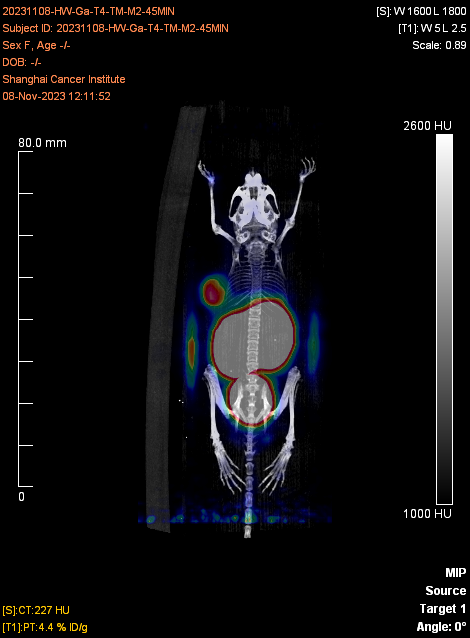

Supplement: Supplementary file 5 — Source data Fig. 3 [file 44321_2024_59_MOESM5_ESM.zip › Figure 3/3A/T4-45MIN-0-5-MIP .png]

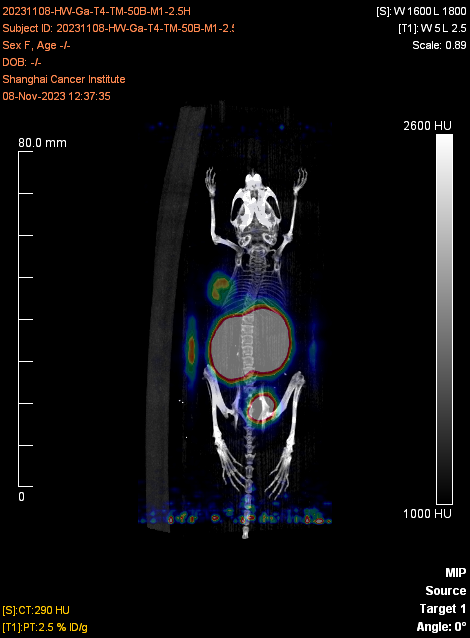

Supplement: Supplementary file 5 — Source data Fig. 3 [file 44321_2024_59_MOESM5_ESM.zip › Figure 3/3B/T4-50B-2.5H-0-5-MIP.png]

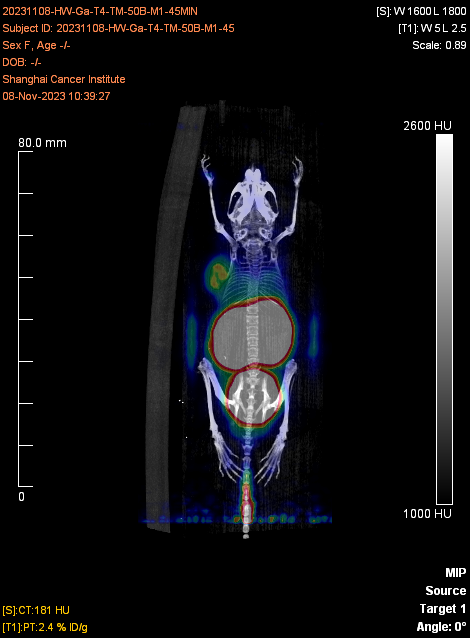

Supplement: Supplementary file 5 — Source data Fig. 3 [file 44321_2024_59_MOESM5_ESM.zip › Figure 3/3B/T4-50B-45MIN-0-5-MIP.png]

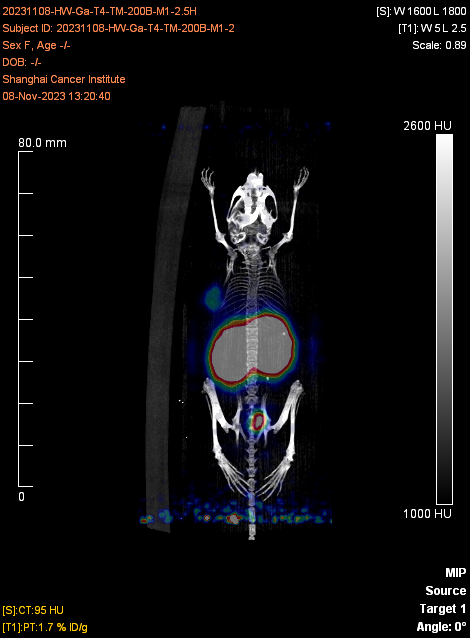

Supplement: Supplementary file 5 — Source data Fig. 3 [file 44321_2024_59_MOESM5_ESM.zip › Figure 3/3C/T4-200B-2.5H-0-5-MIP.png]

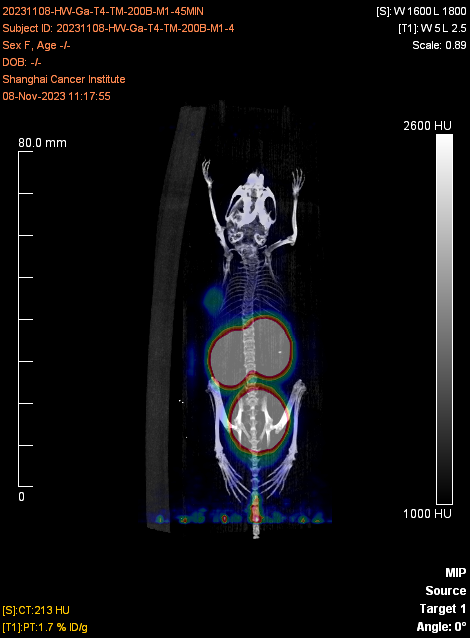

Supplement: Supplementary file 5 — Source data Fig. 3 [file 44321_2024_59_MOESM5_ESM.zip › Figure 3/3C/T4-200B-45MIN-0-5-MIP.png]

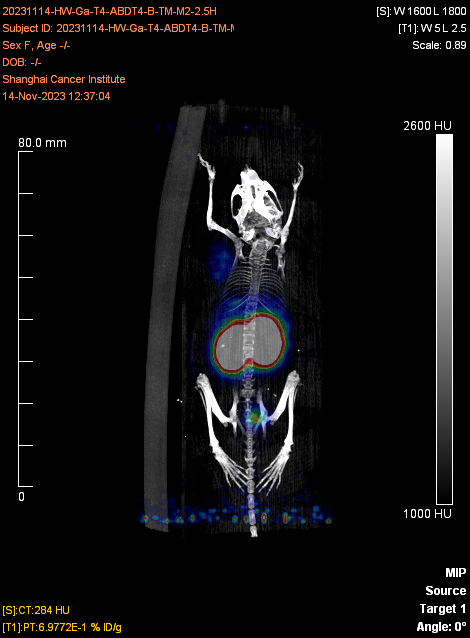

Supplement: Supplementary file 5 — Source data Fig. 3 [file 44321_2024_59_MOESM5_ESM.zip › Figure 3/3D/T4-400B-2.5H-0-5-MIP.png]

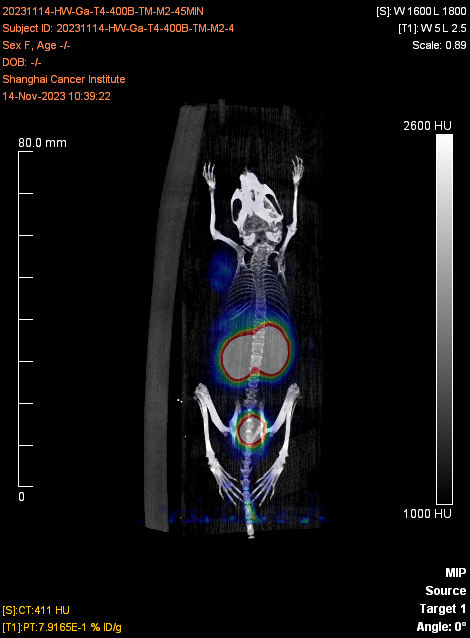

Supplement: Supplementary file 5 — Source data Fig. 3 [file 44321_2024_59_MOESM5_ESM.zip › Figure 3/3D/T4-400B-45MIN-0-5-MIP.png]

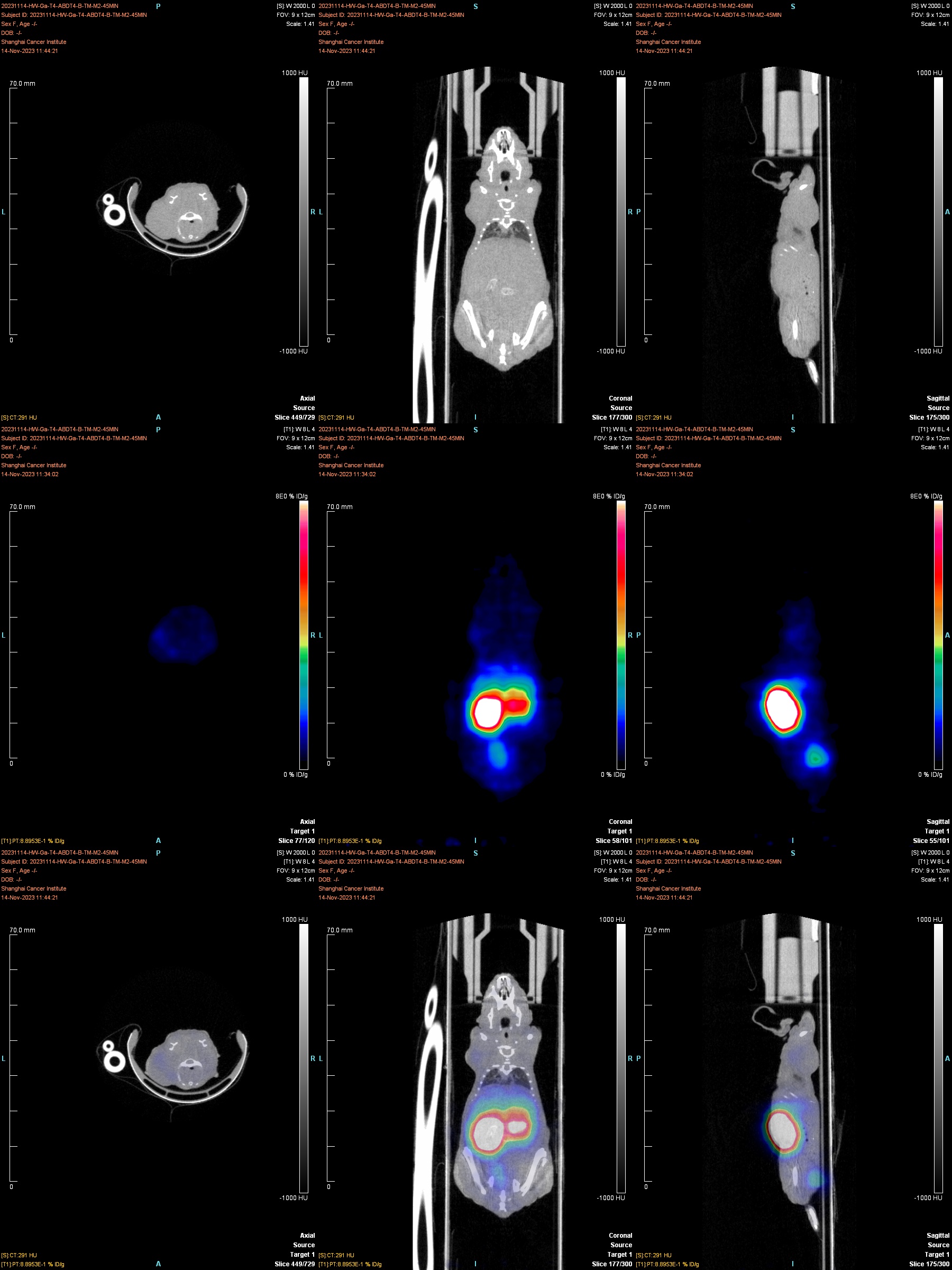

Supplement: Supplementary file 6 — Source data Fig. 4 [file 44321_2024_59_MOESM6_ESM.zip › Figure 4/4A/ABDT4-BLOCK-0-8.jpg]

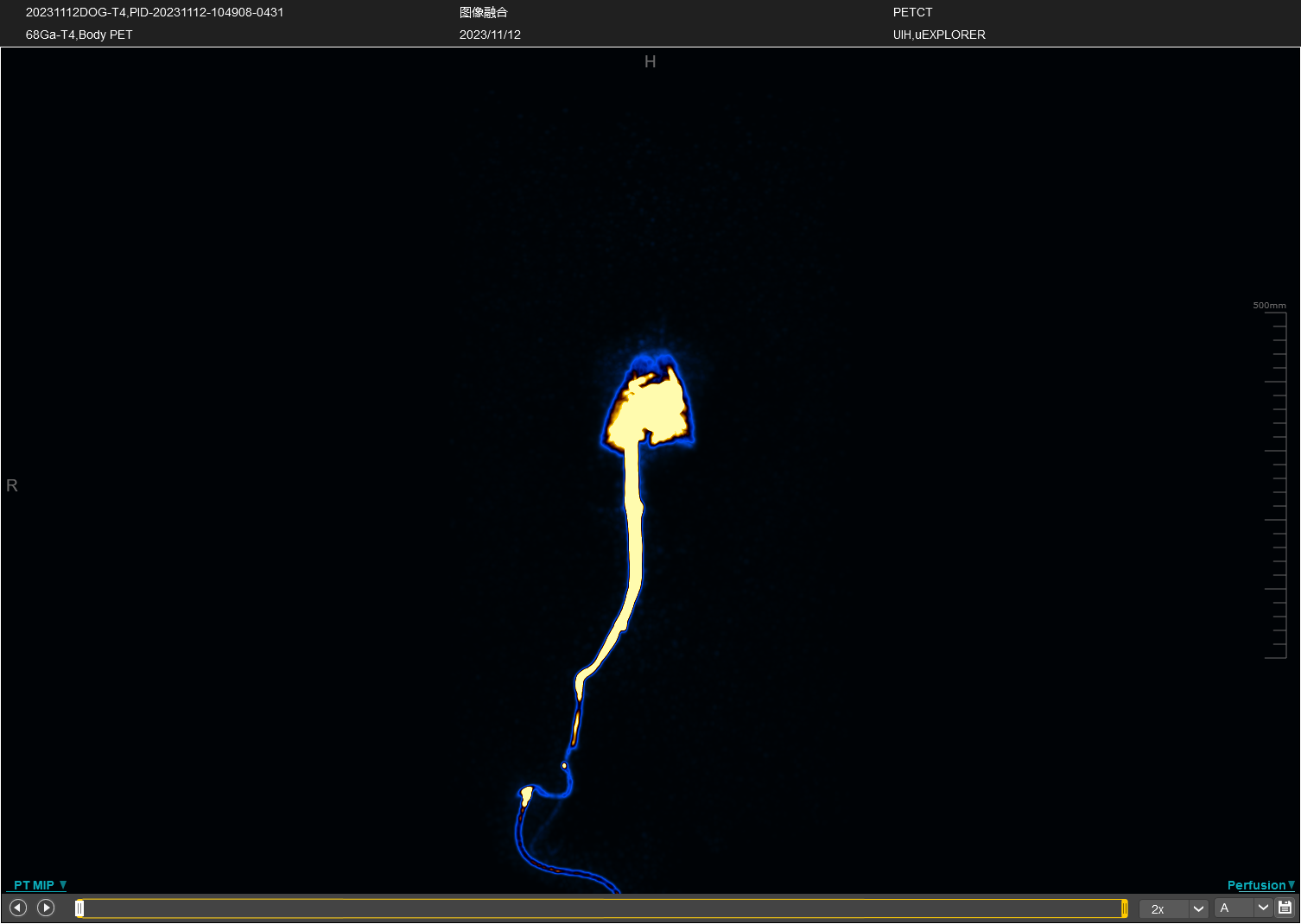

Supplement: Supplementary file 7 — Source data Fig. 5 [file 44321_2024_59_MOESM7_ESM.zip › Figure 5/5A/15 s.png]

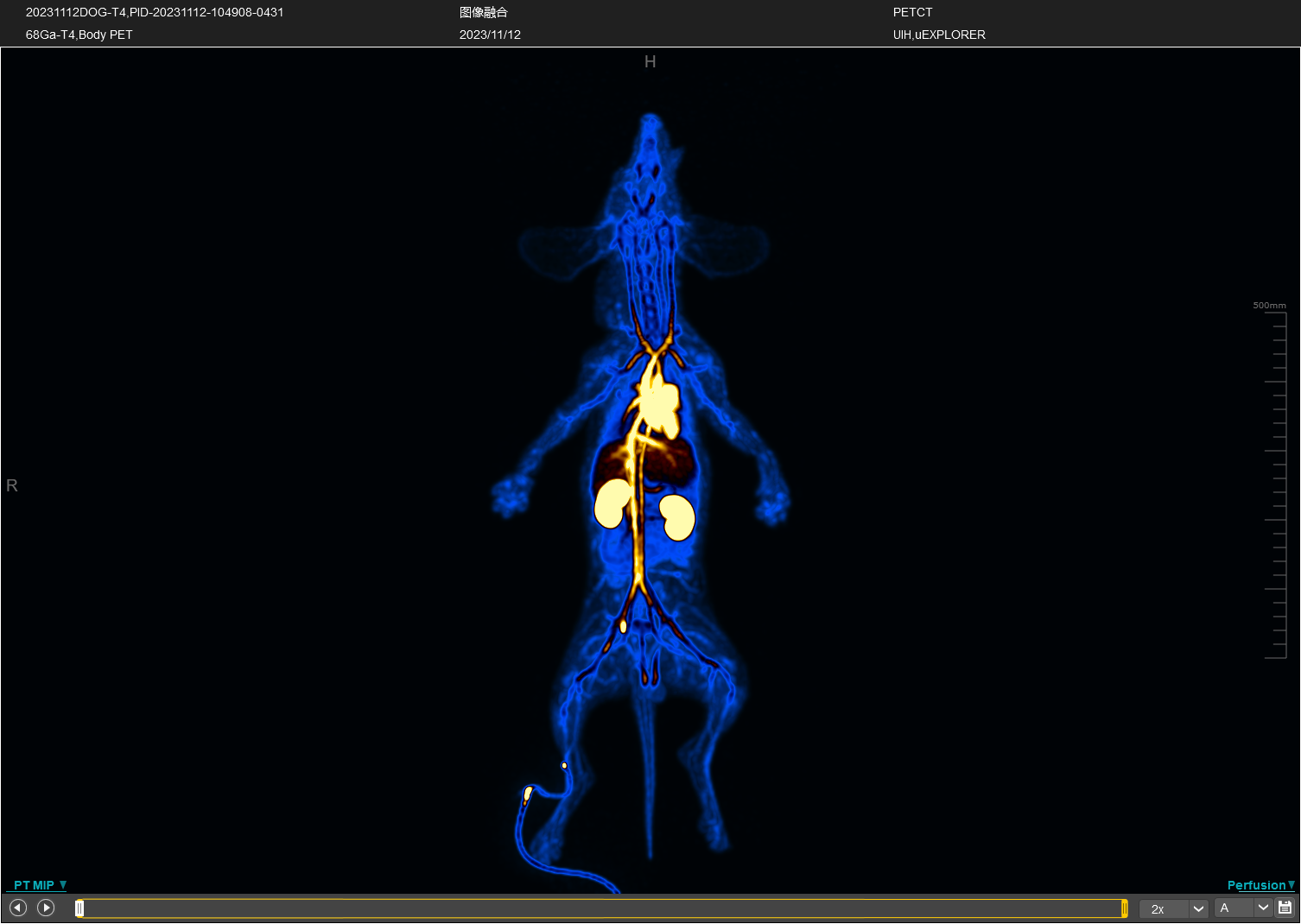

Supplement: Supplementary file 7 — Source data Fig. 5 [file 44321_2024_59_MOESM7_ESM.zip › Figure 5/5A/2 min.png]

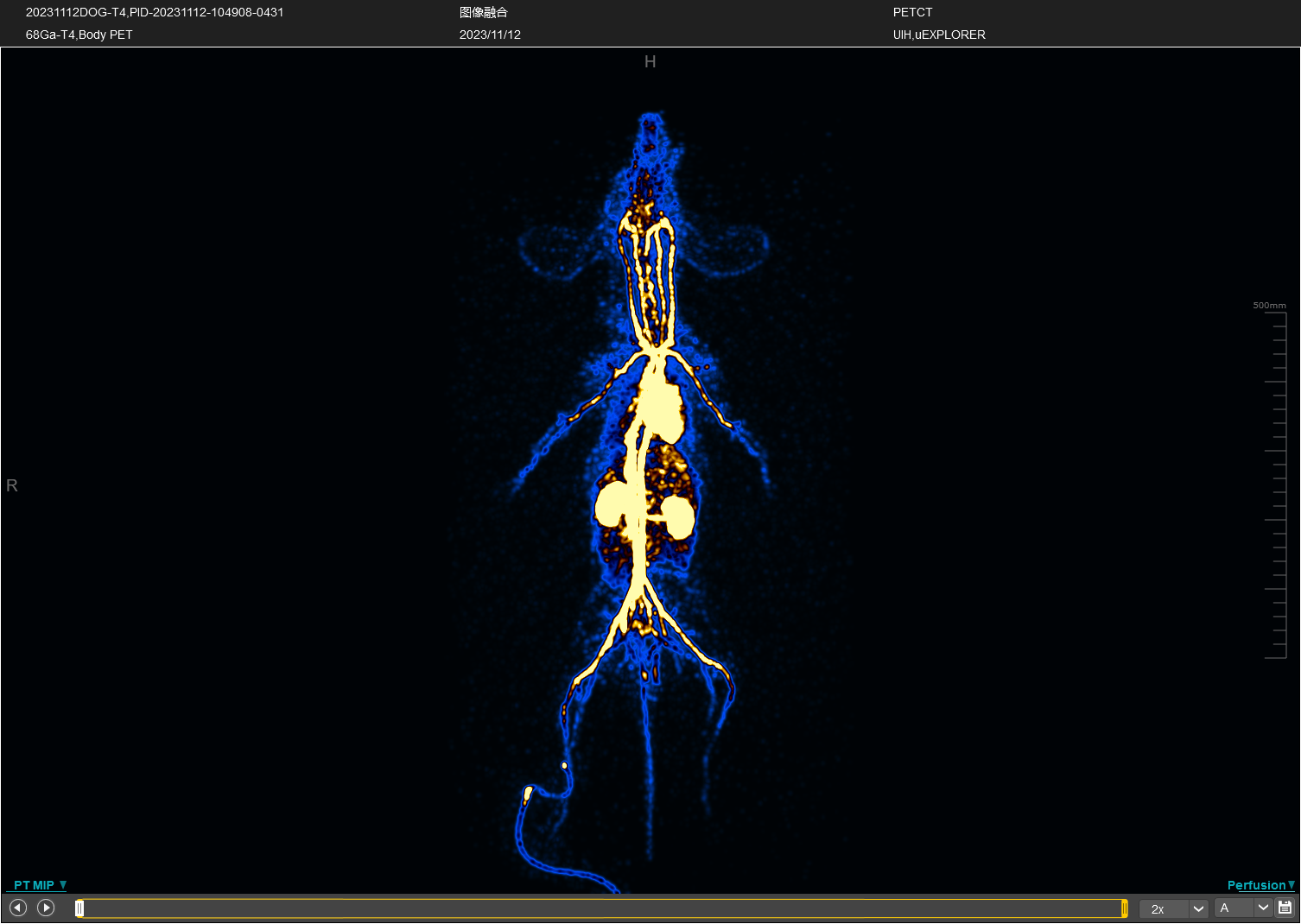

Supplement: Supplementary file 7 — Source data Fig. 5 [file 44321_2024_59_MOESM7_ESM.zip › Figure 5/5A/25 s.png]

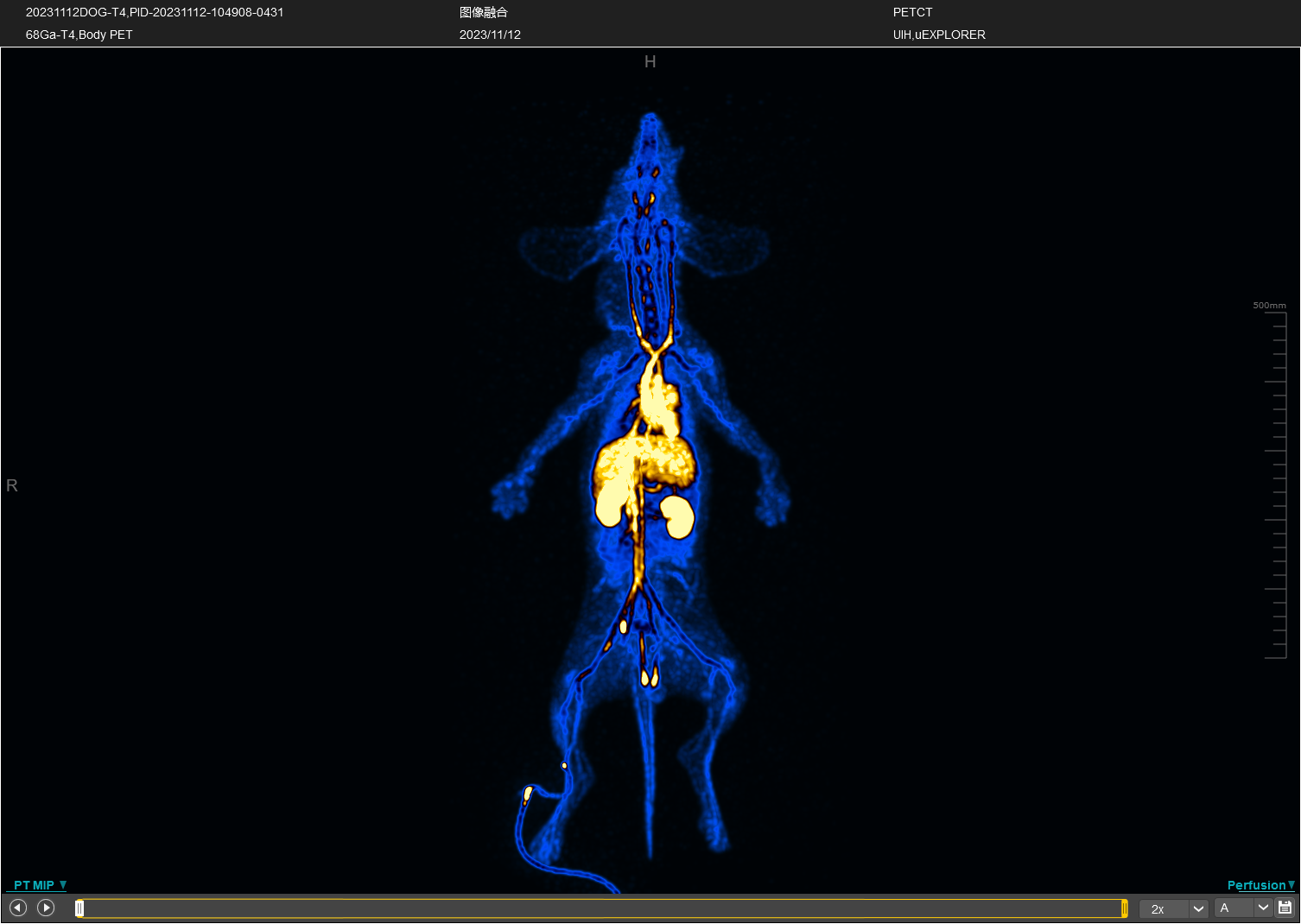

Supplement: Supplementary file 7 — Source data Fig. 5 [file 44321_2024_59_MOESM7_ESM.zip › Figure 5/5A/45 s.png]

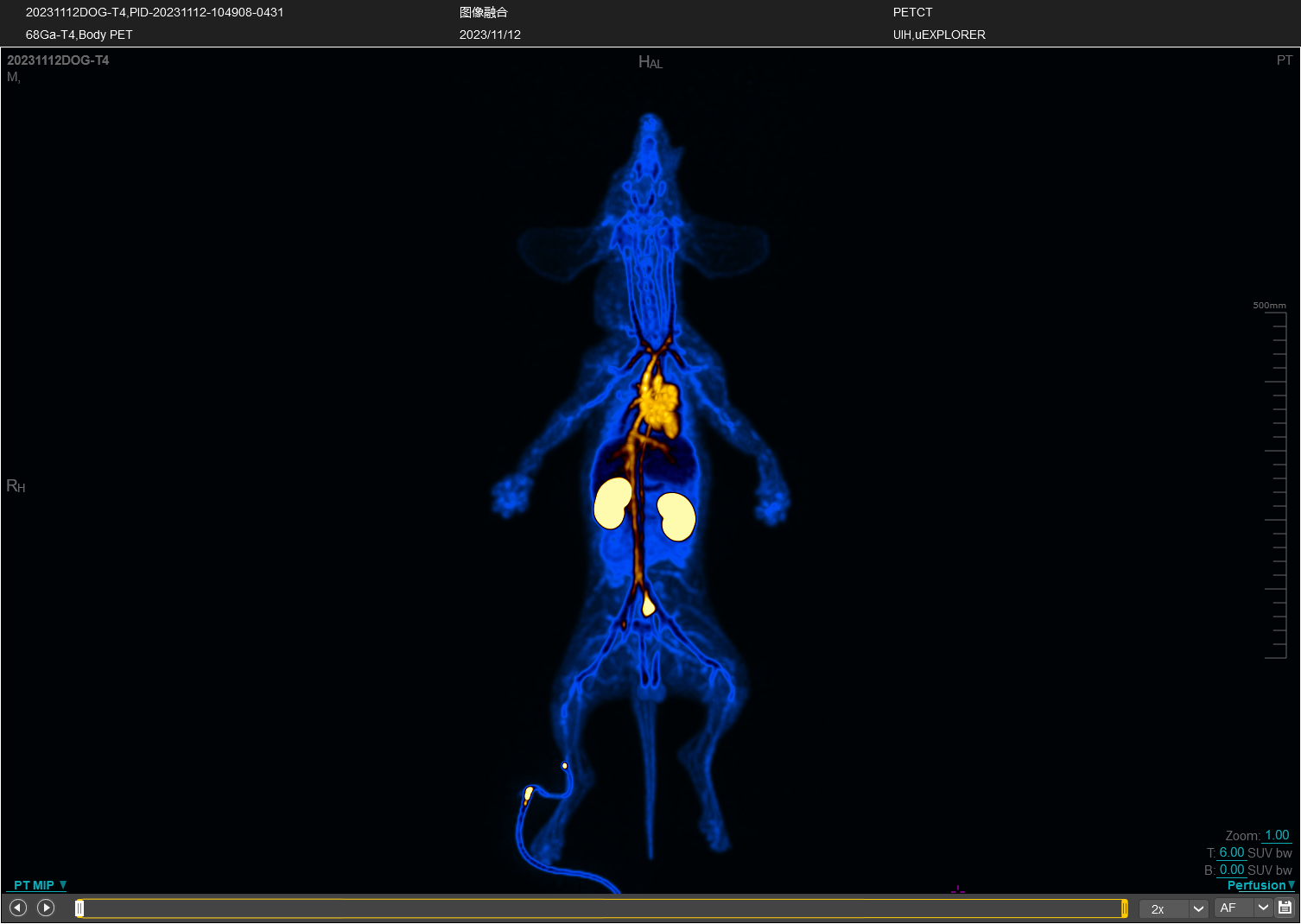

Supplement: Supplementary file 7 — Source data Fig. 5 [file 44321_2024_59_MOESM7_ESM.zip › Figure 5/5A/5 min.png]

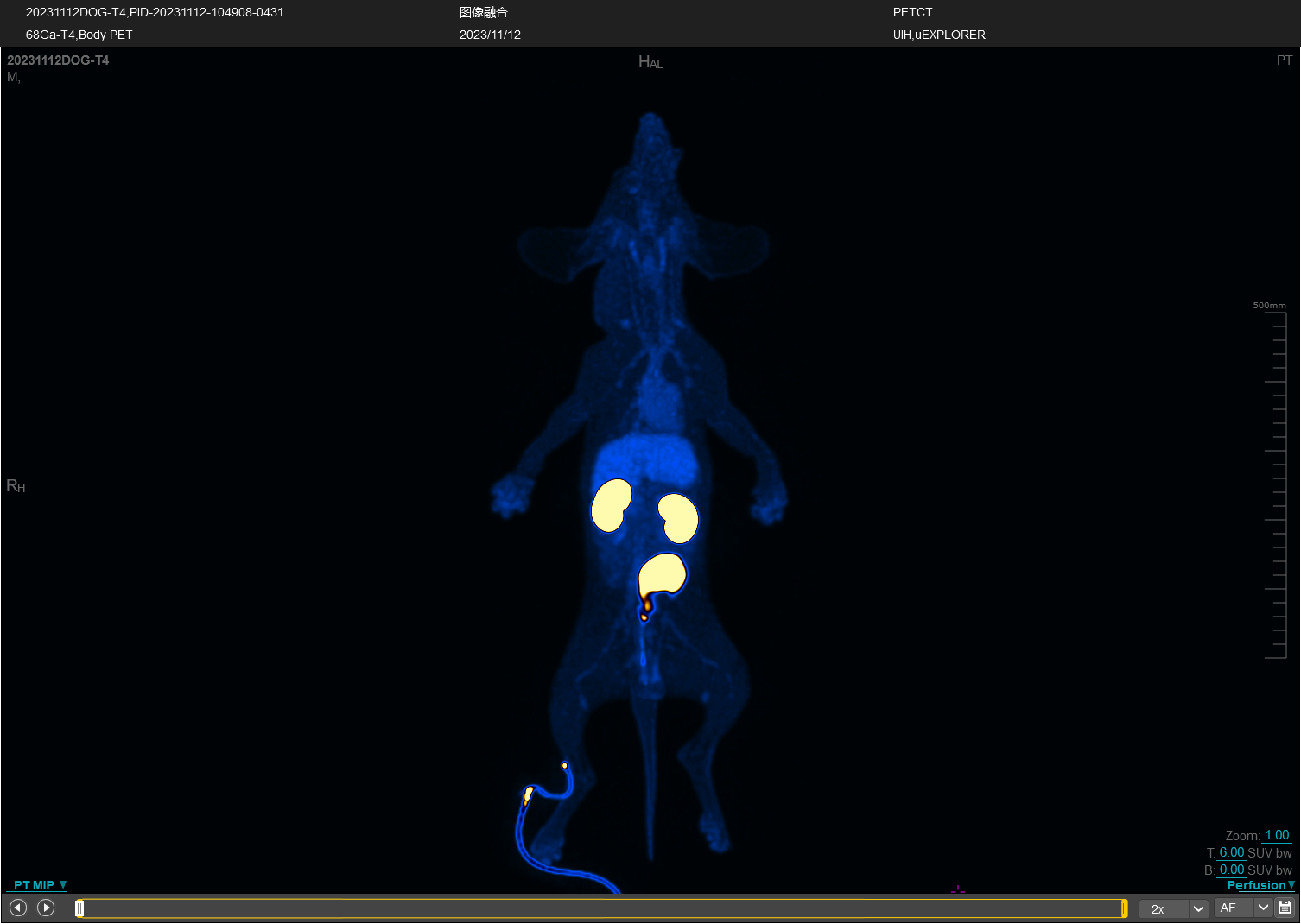

Supplement: Supplementary file 7 — Source data Fig. 5 [file 44321_2024_59_MOESM7_ESM.zip › Figure 5/5A/60 min.png]

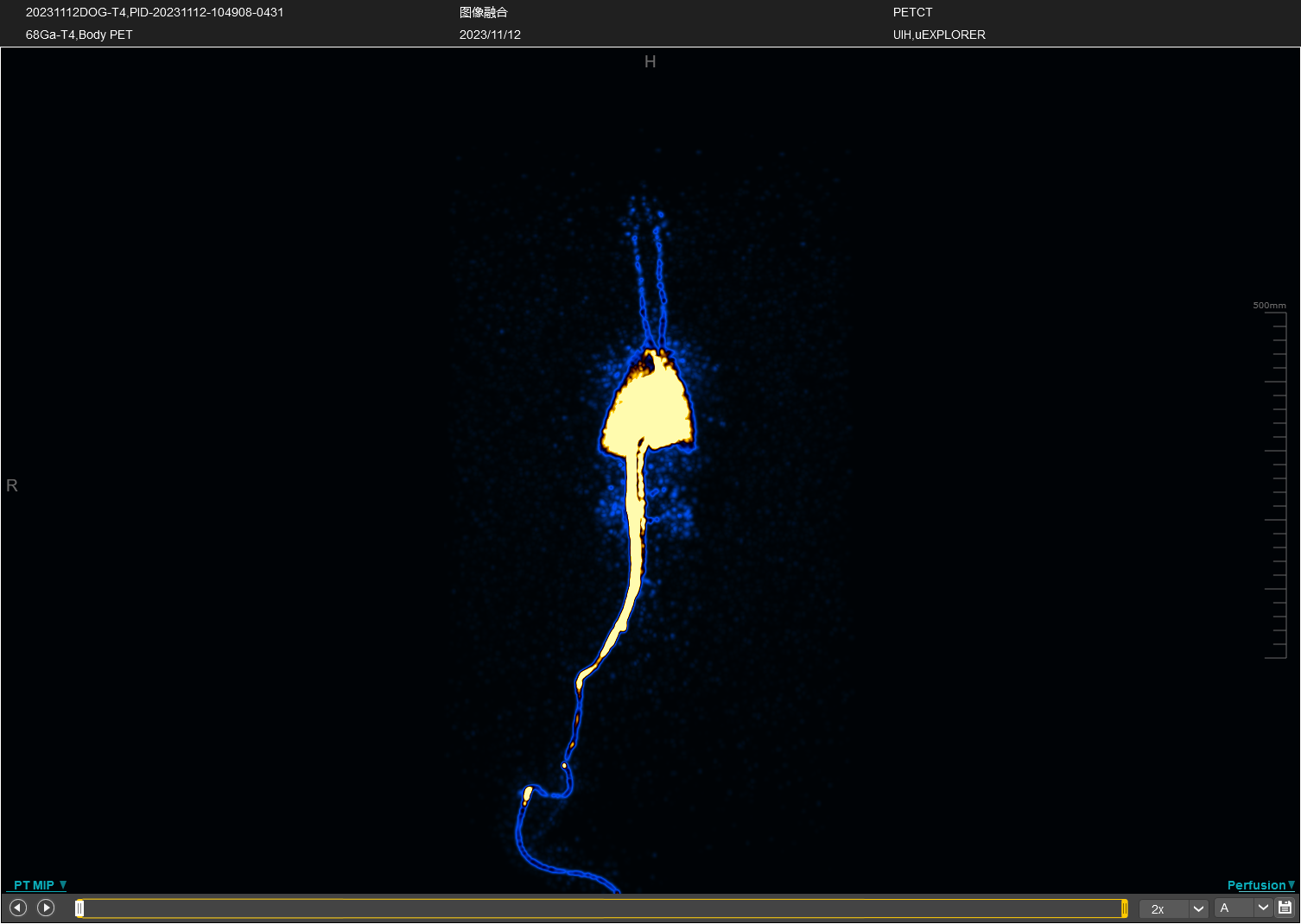

Supplement: Supplementary file 7 — Source data Fig. 5 [file 44321_2024_59_MOESM7_ESM.zip › Figure 5/5C/16 s.png]

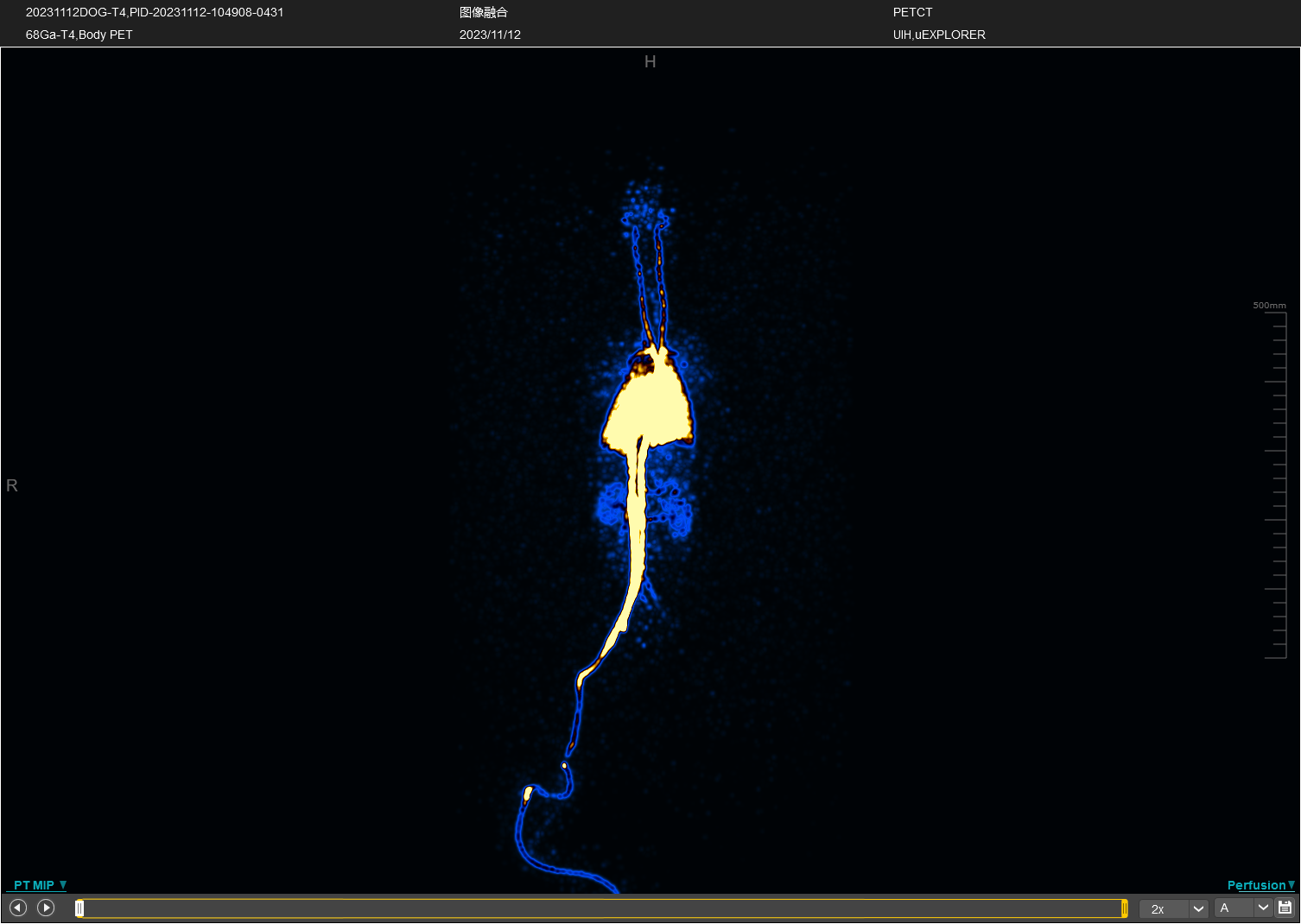

Supplement: Supplementary file 7 — Source data Fig. 5 [file 44321_2024_59_MOESM7_ESM.zip › Figure 5/5C/17 s.png]

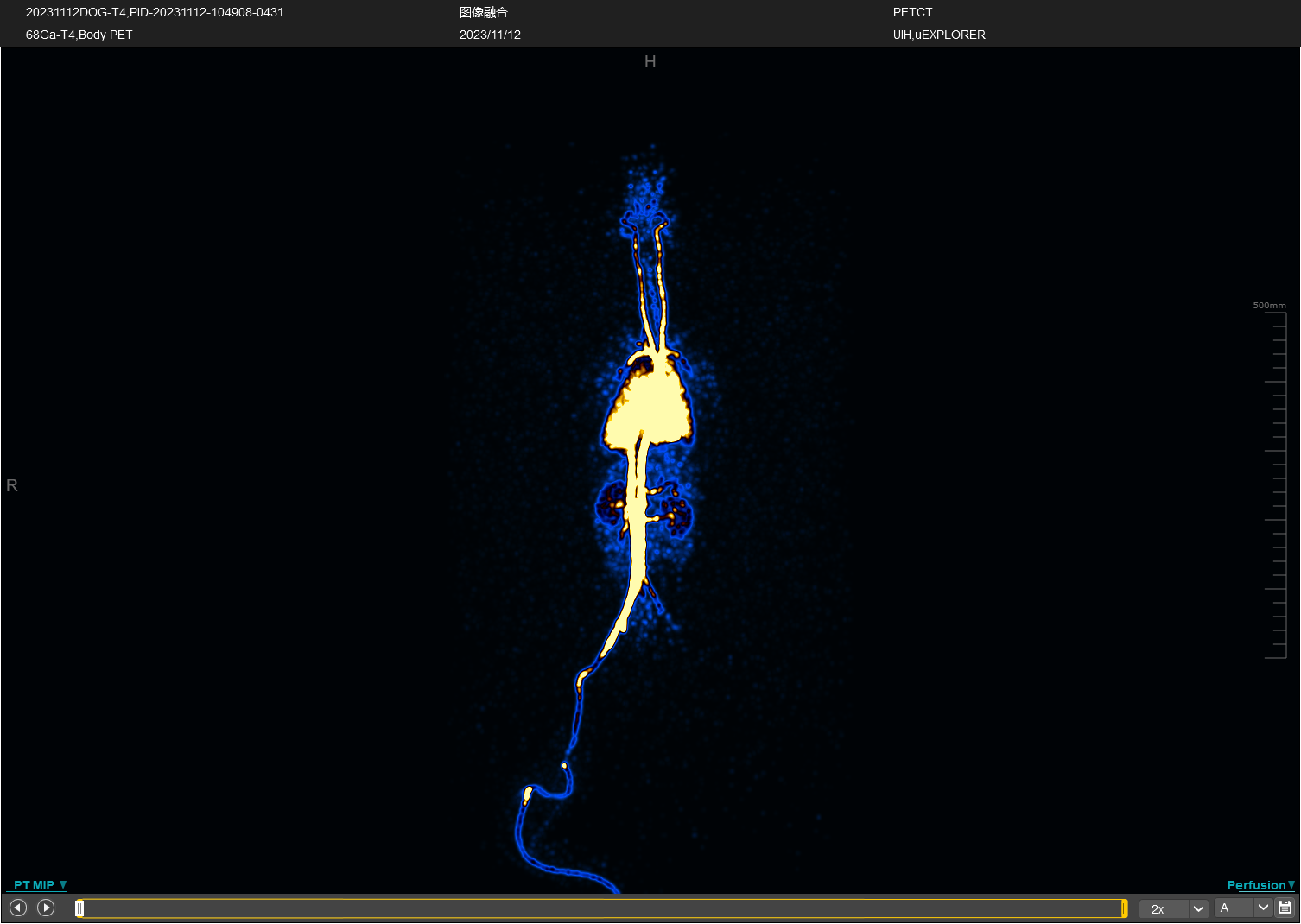

Supplement: Supplementary file 7 — Source data Fig. 5 [file 44321_2024_59_MOESM7_ESM.zip › Figure 5/5C/18 s.png]

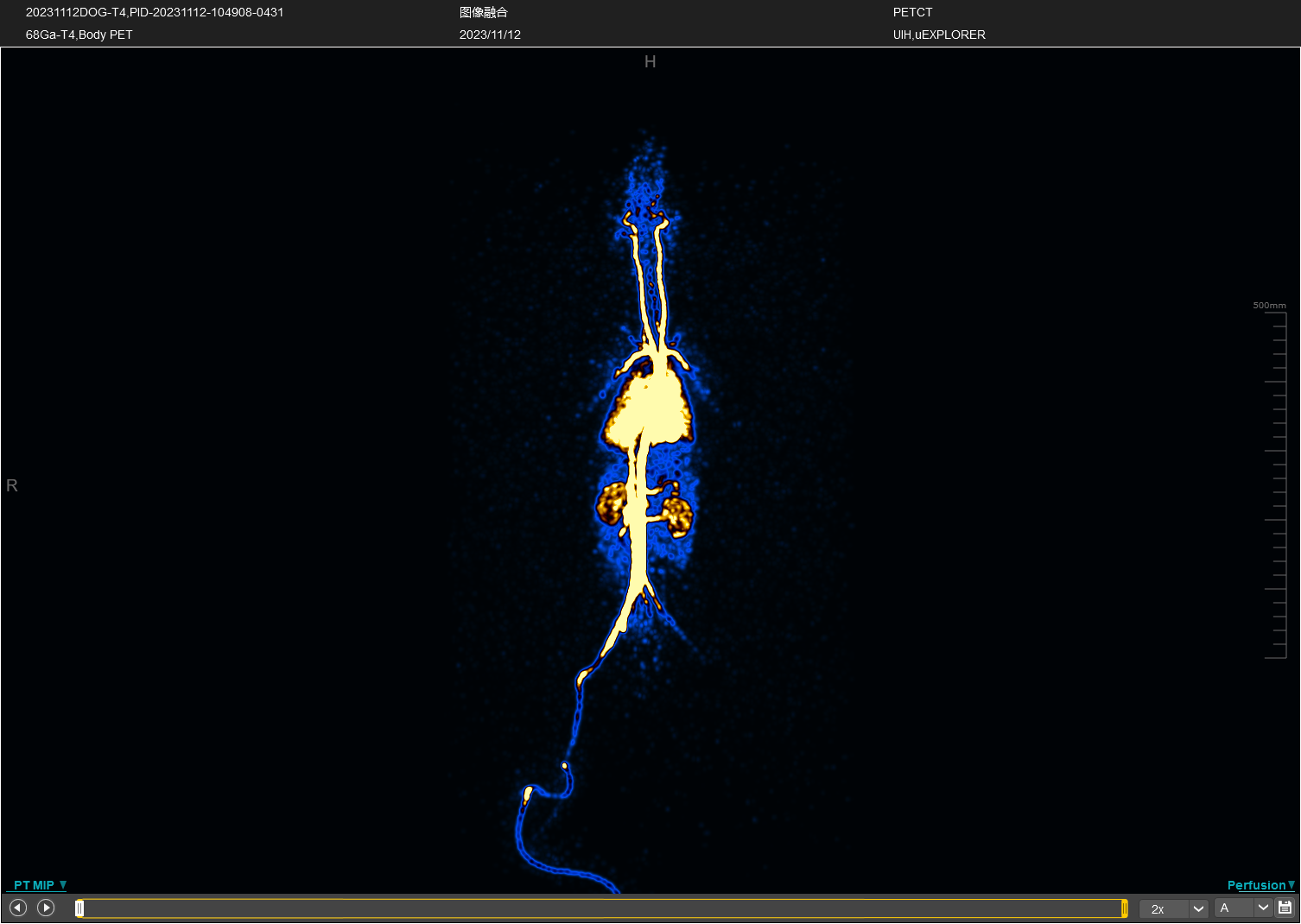

Supplement: Supplementary file 7 — Source data Fig. 5 [file 44321_2024_59_MOESM7_ESM.zip › Figure 5/5C/19 s.png]

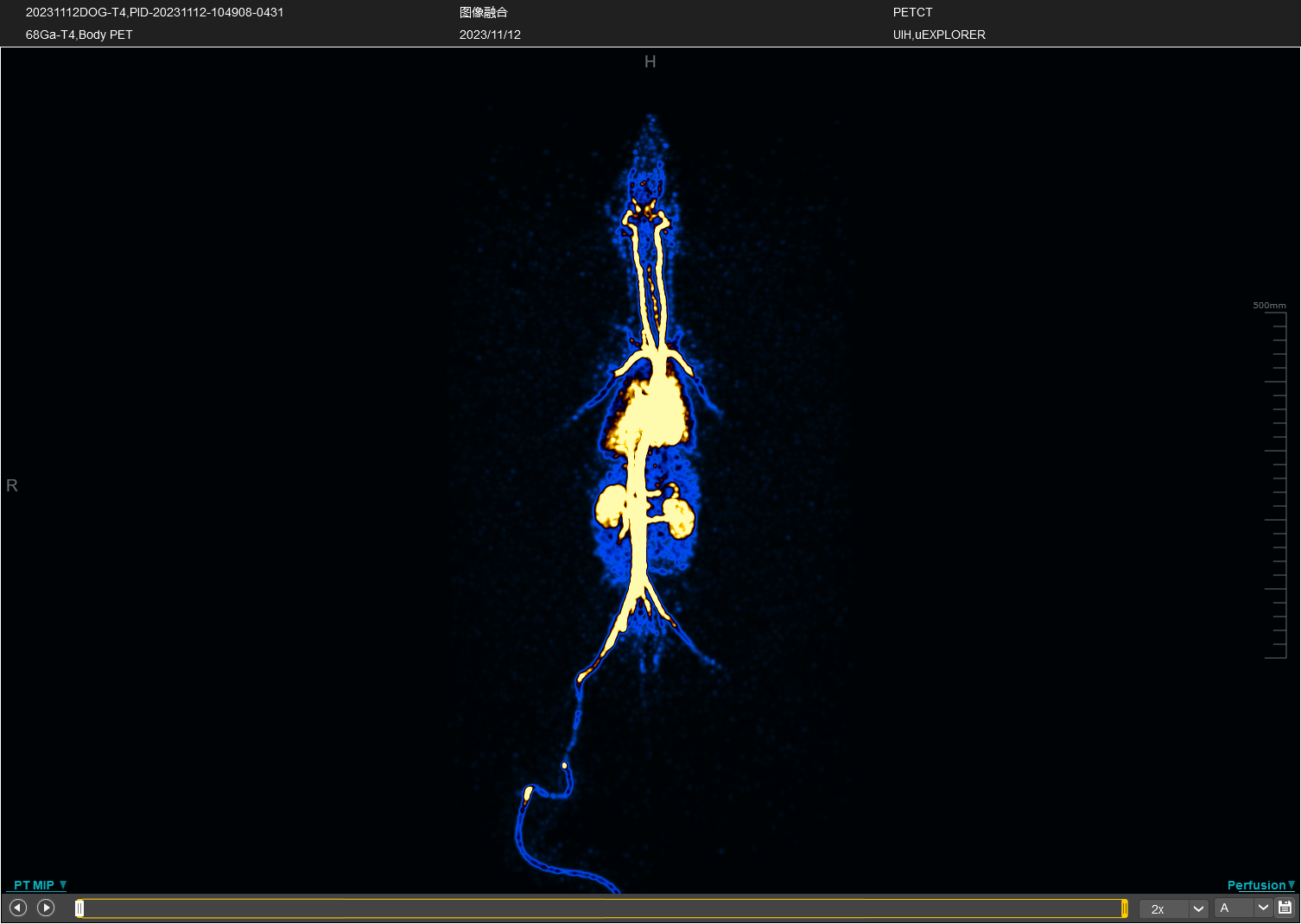

Supplement: Supplementary file 7 — Source data Fig. 5 [file 44321_2024_59_MOESM7_ESM.zip › Figure 5/5C/20 s.png]

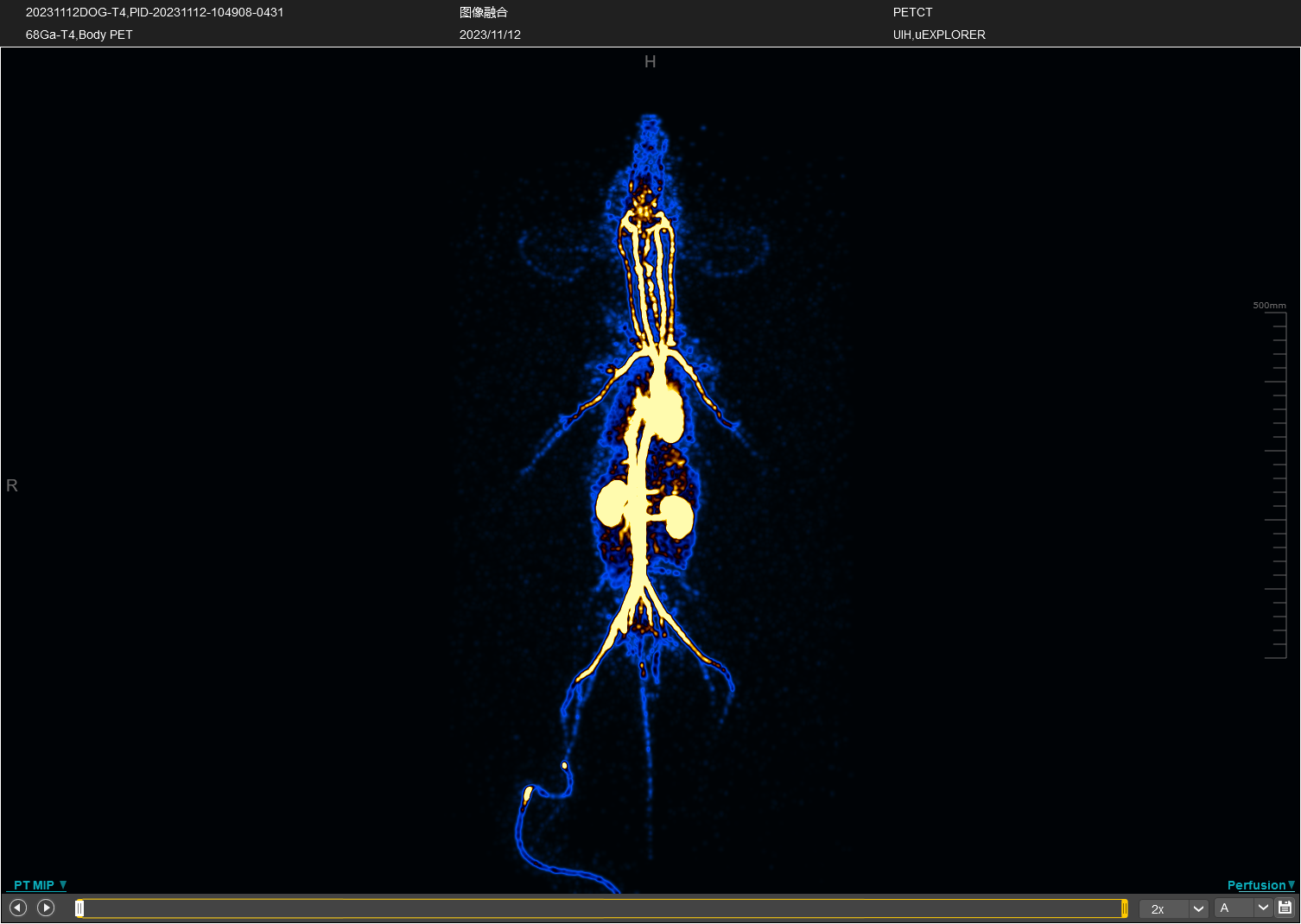

Supplement: Supplementary file 7 — Source data Fig. 5 [file 44321_2024_59_MOESM7_ESM.zip › Figure 5/5C/23 s.png]

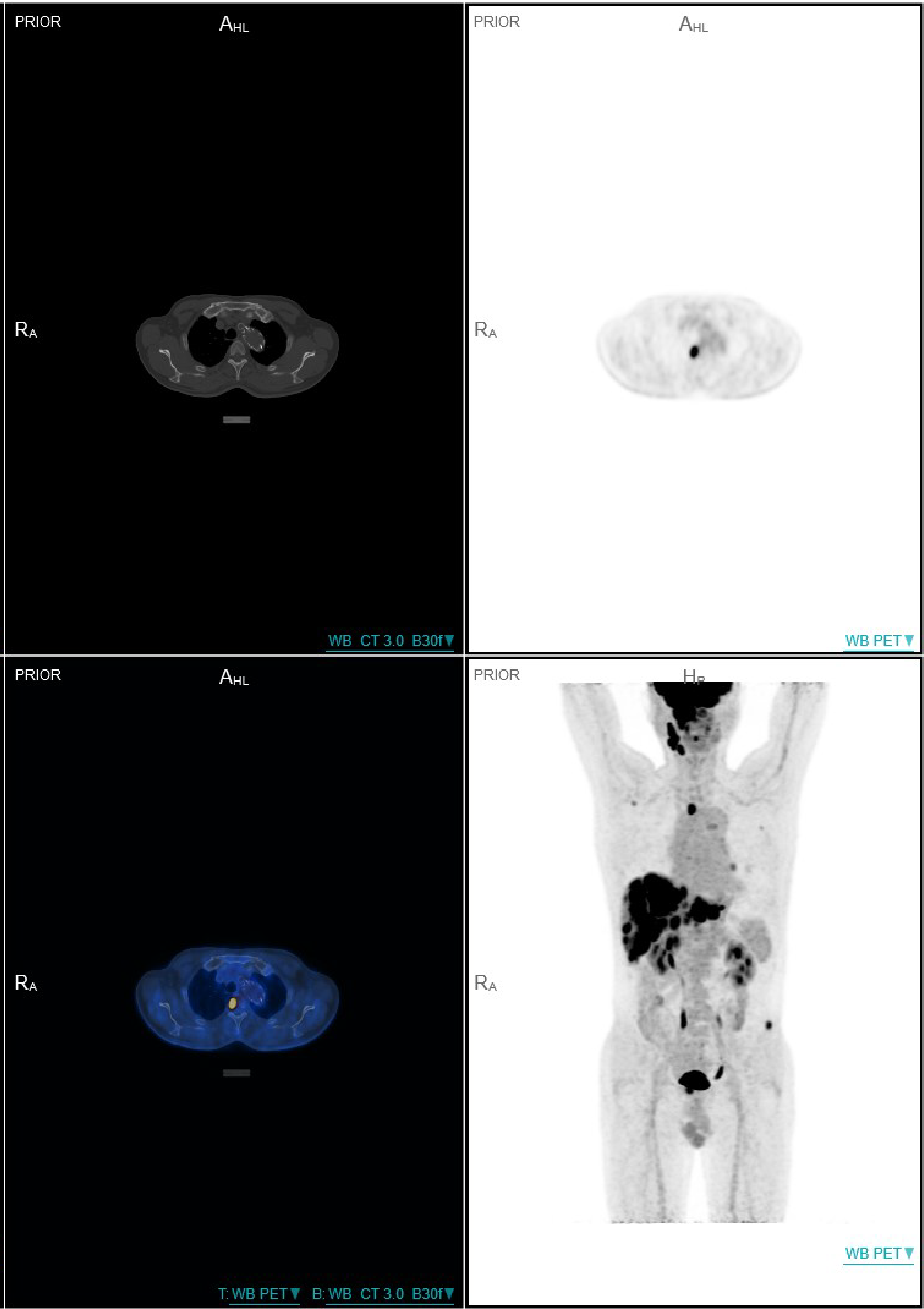

Supplement: Supplementary file 8 — Source data Fig. 6 [file 44321_2024_59_MOESM8_ESM.zip › Figure 6/6A–C/6A.png]

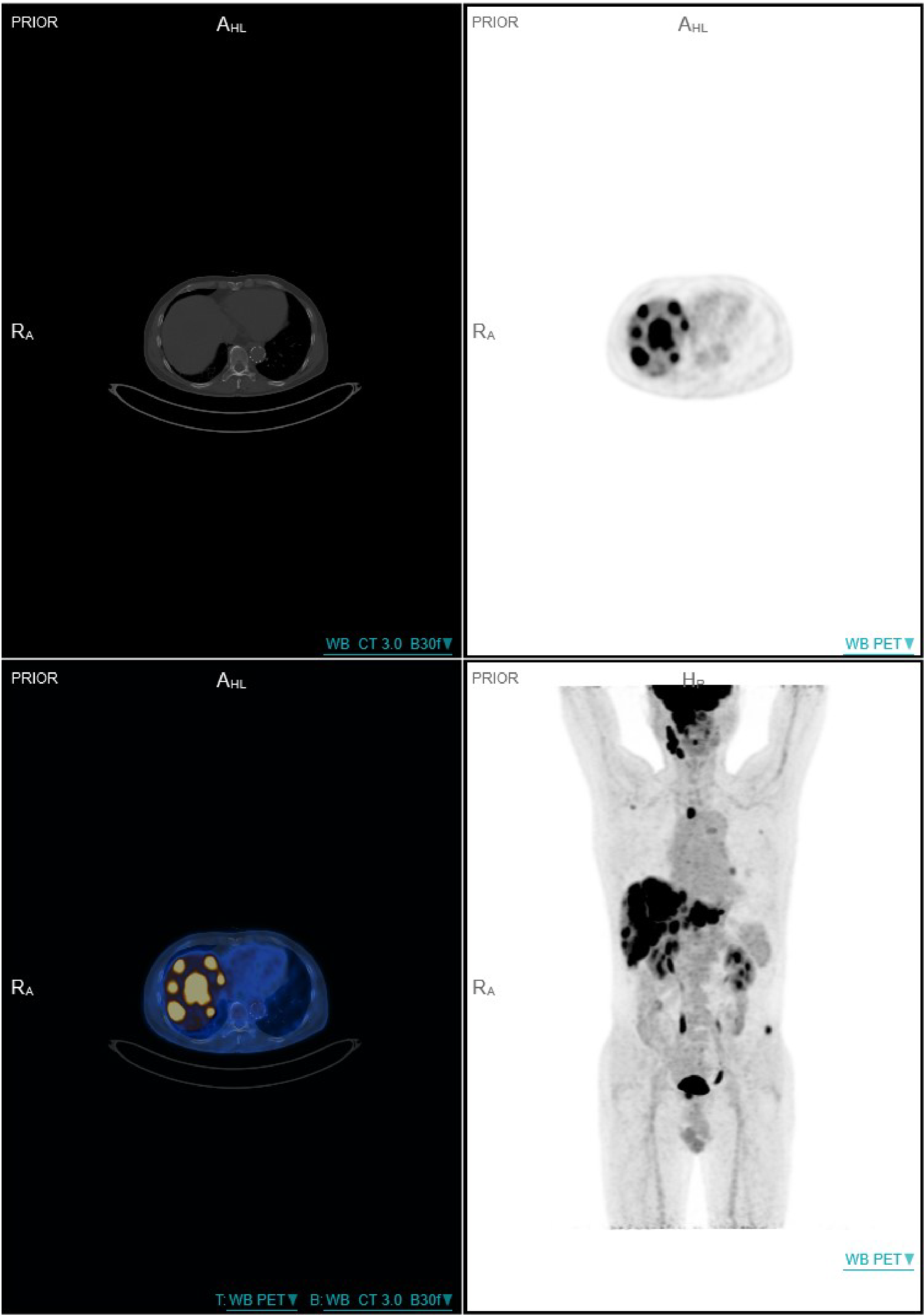

Supplement: Supplementary file 8 — Source data Fig. 6 [file 44321_2024_59_MOESM8_ESM.zip › Figure 6/6A–C/6B.png]

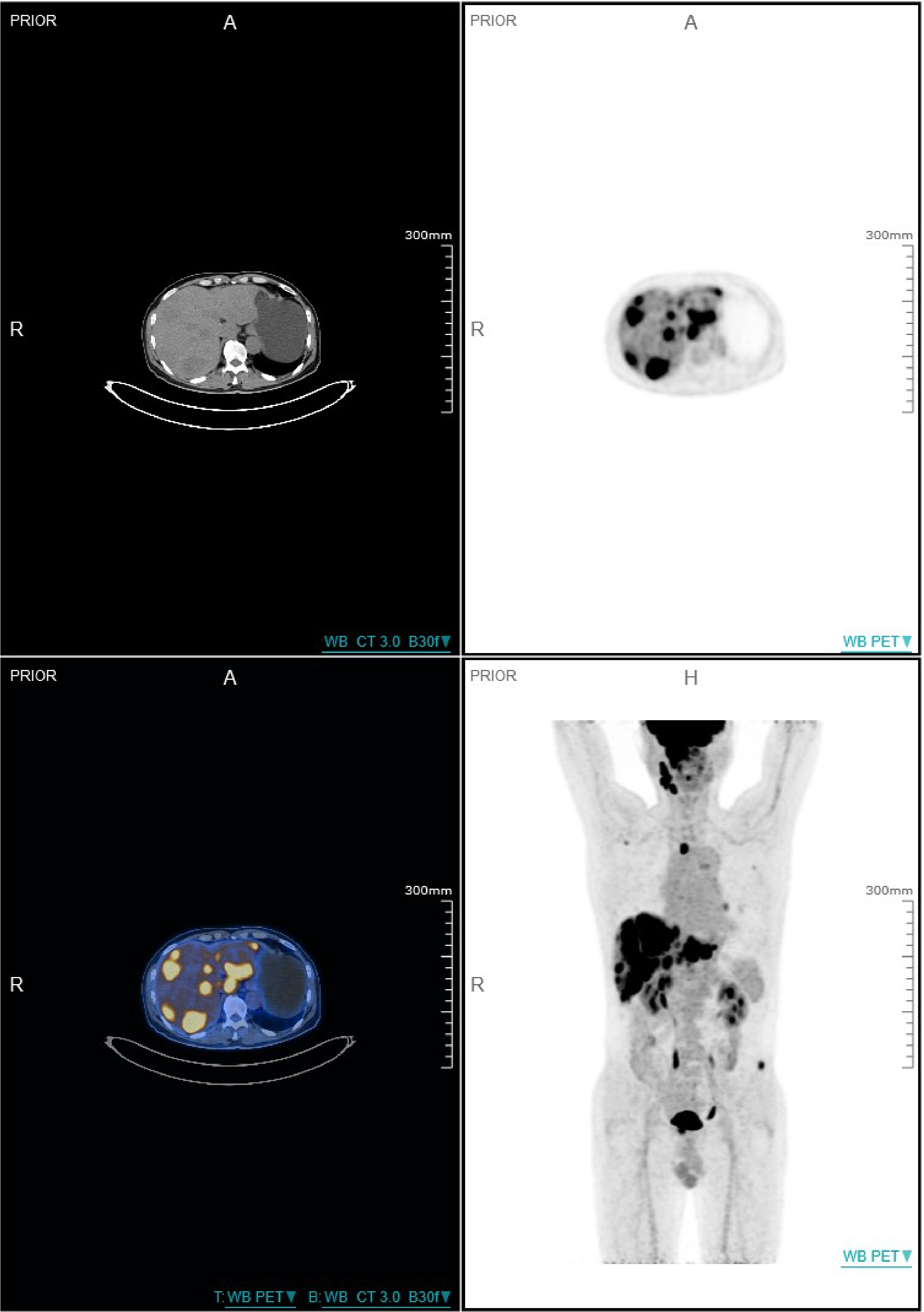

Supplement: Supplementary file 8 — Source data Fig. 6 [file 44321_2024_59_MOESM8_ESM.zip › Figure 6/6A–C/6C.png]

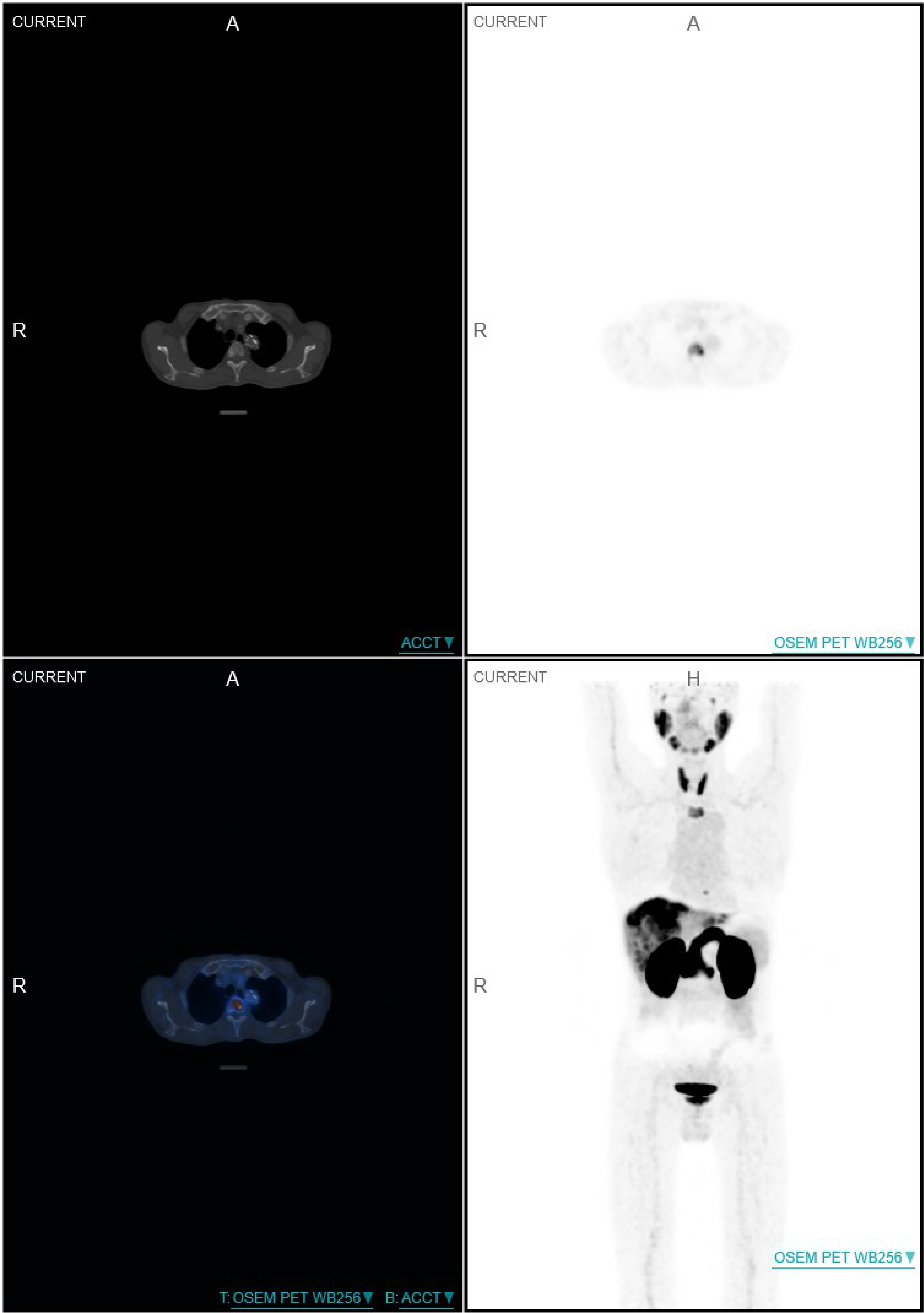

Supplement: Supplementary file 8 — Source data Fig. 6 [file 44321_2024_59_MOESM8_ESM.zip › Figure 6/6D–F/6D.png]

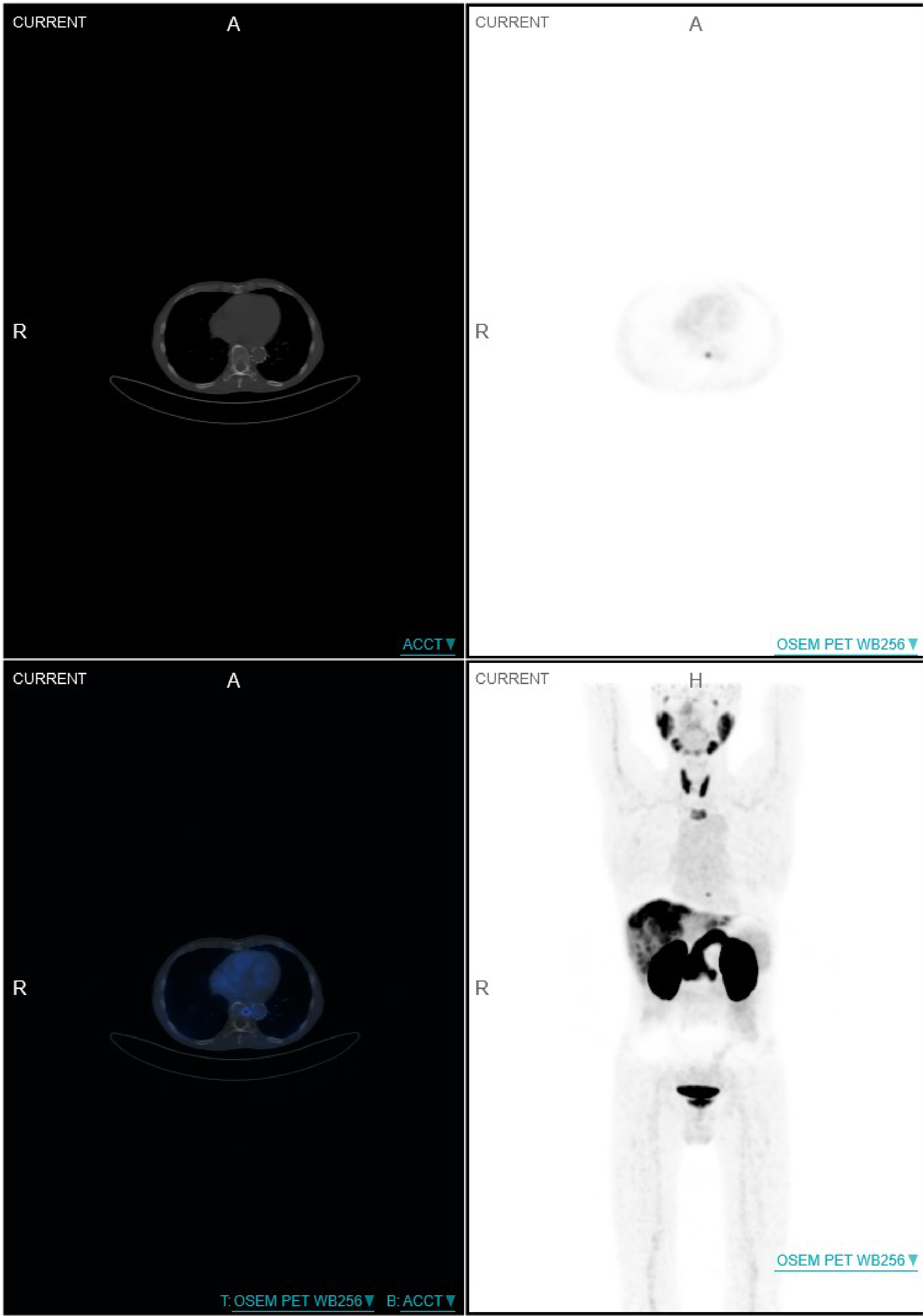

Supplement: Supplementary file 8 — Source data Fig. 6 [file 44321_2024_59_MOESM8_ESM.zip › Figure 6/6D–F/6E.png]

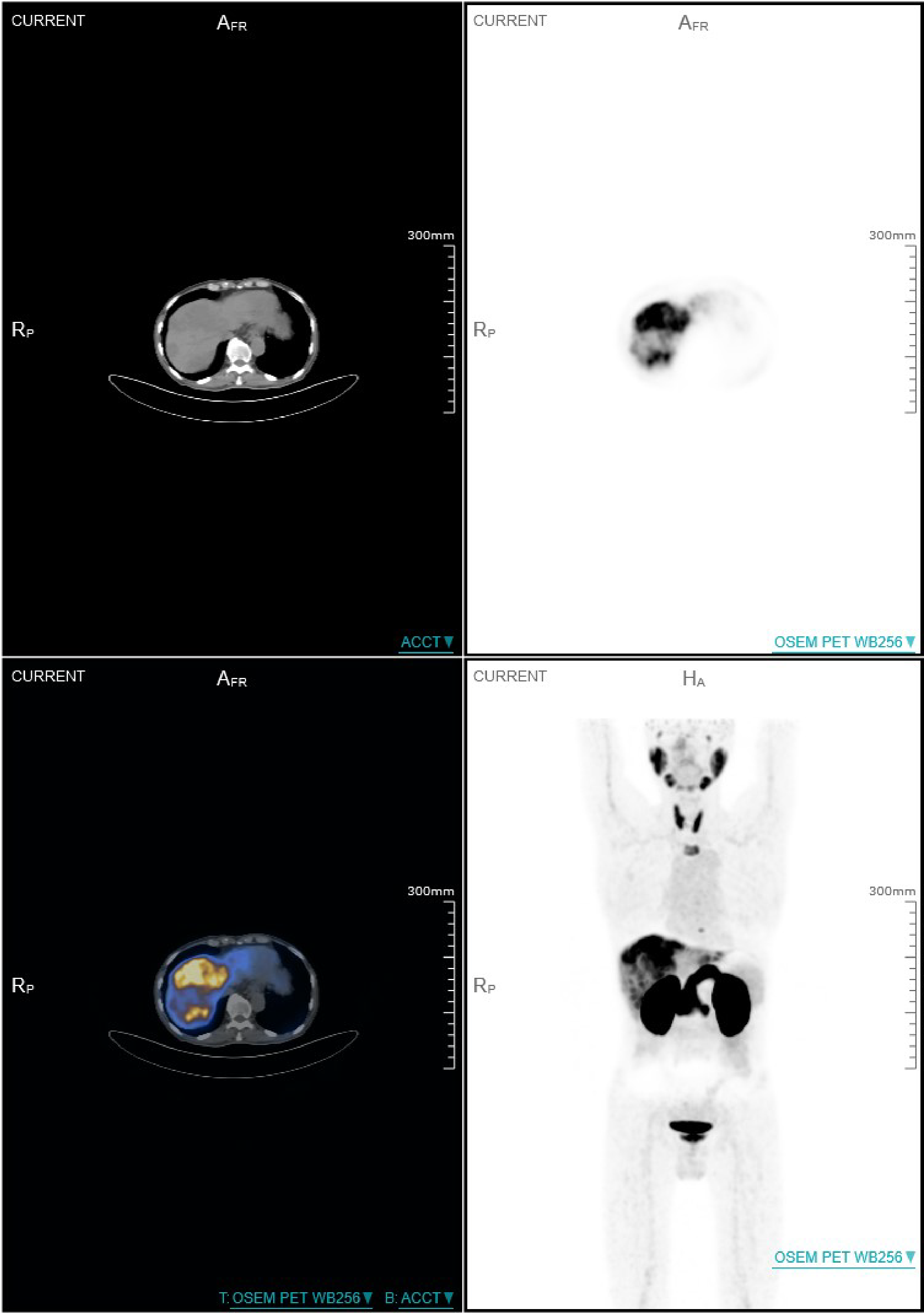

Supplement: Supplementary file 8 — Source data Fig. 6 [file 44321_2024_59_MOESM8_ESM.zip › Figure 6/6D–F/6F.png]

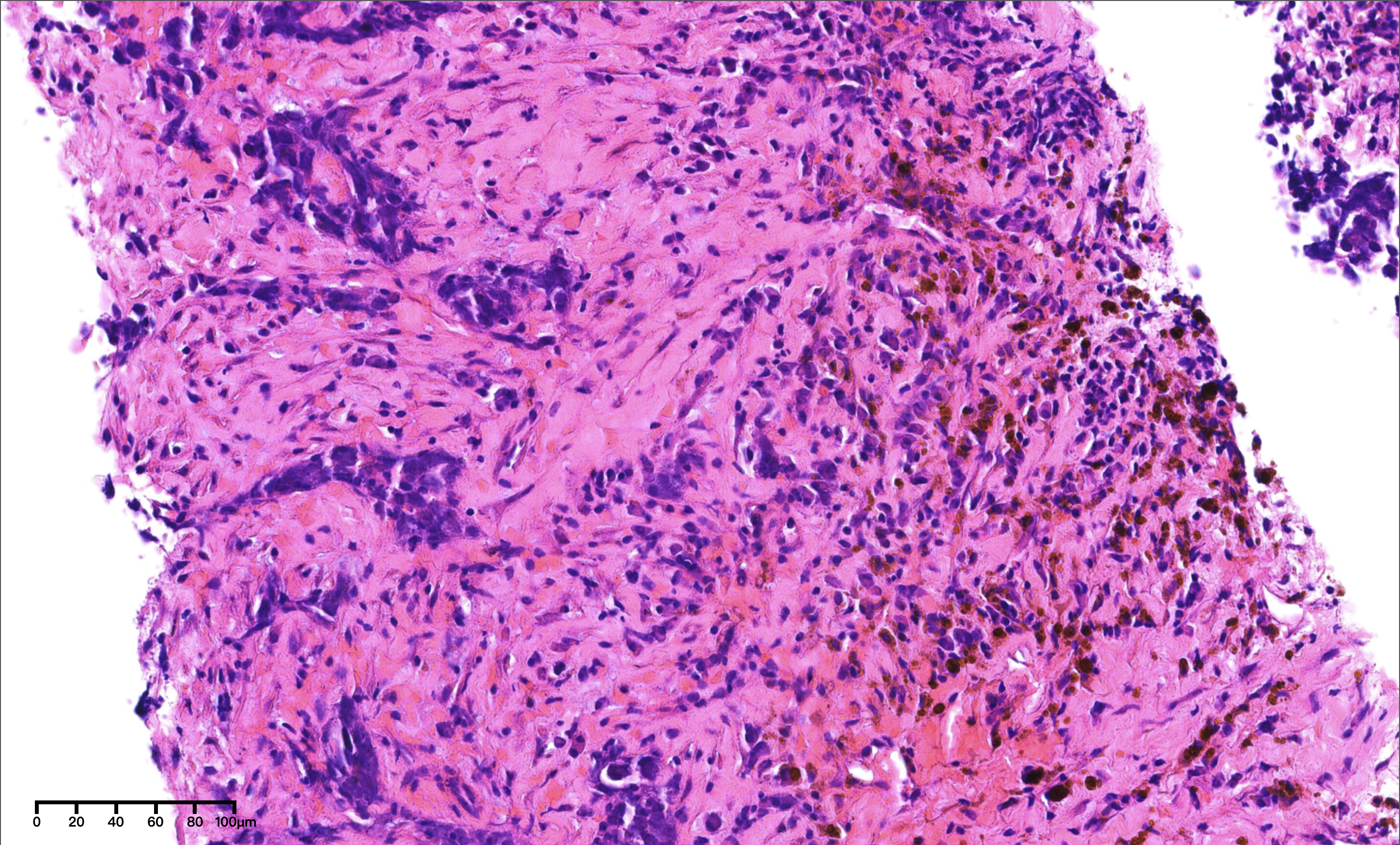

Supplement: Supplementary file 8 — Source data Fig. 6 [file 44321_2024_59_MOESM8_ESM.zip › Figure 6/6G/H&E 100.png]

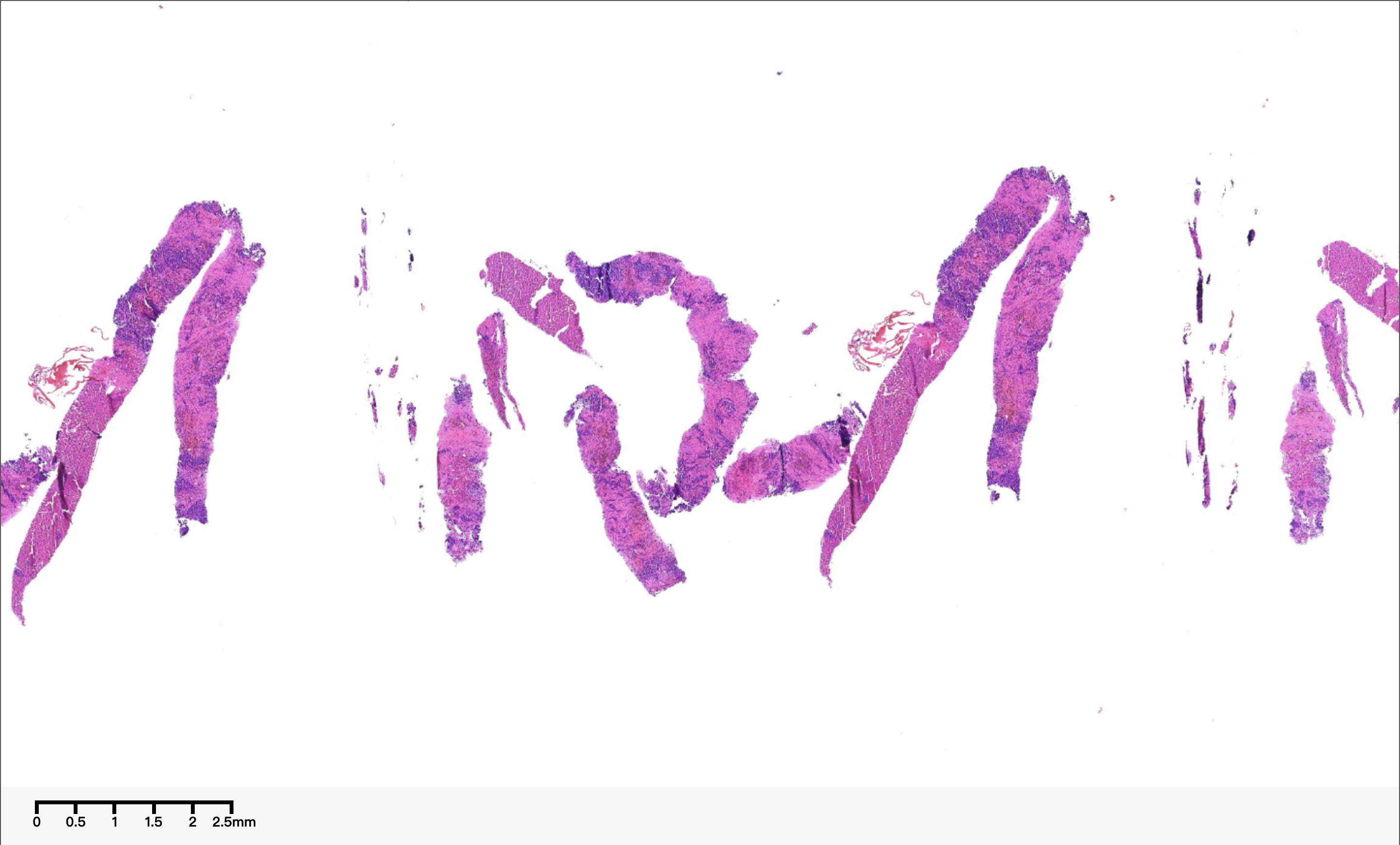

Supplement: Supplementary file 8 — Source data Fig. 6 [file 44321_2024_59_MOESM8_ESM.zip › Figure 6/6G/H&E 2500.png]

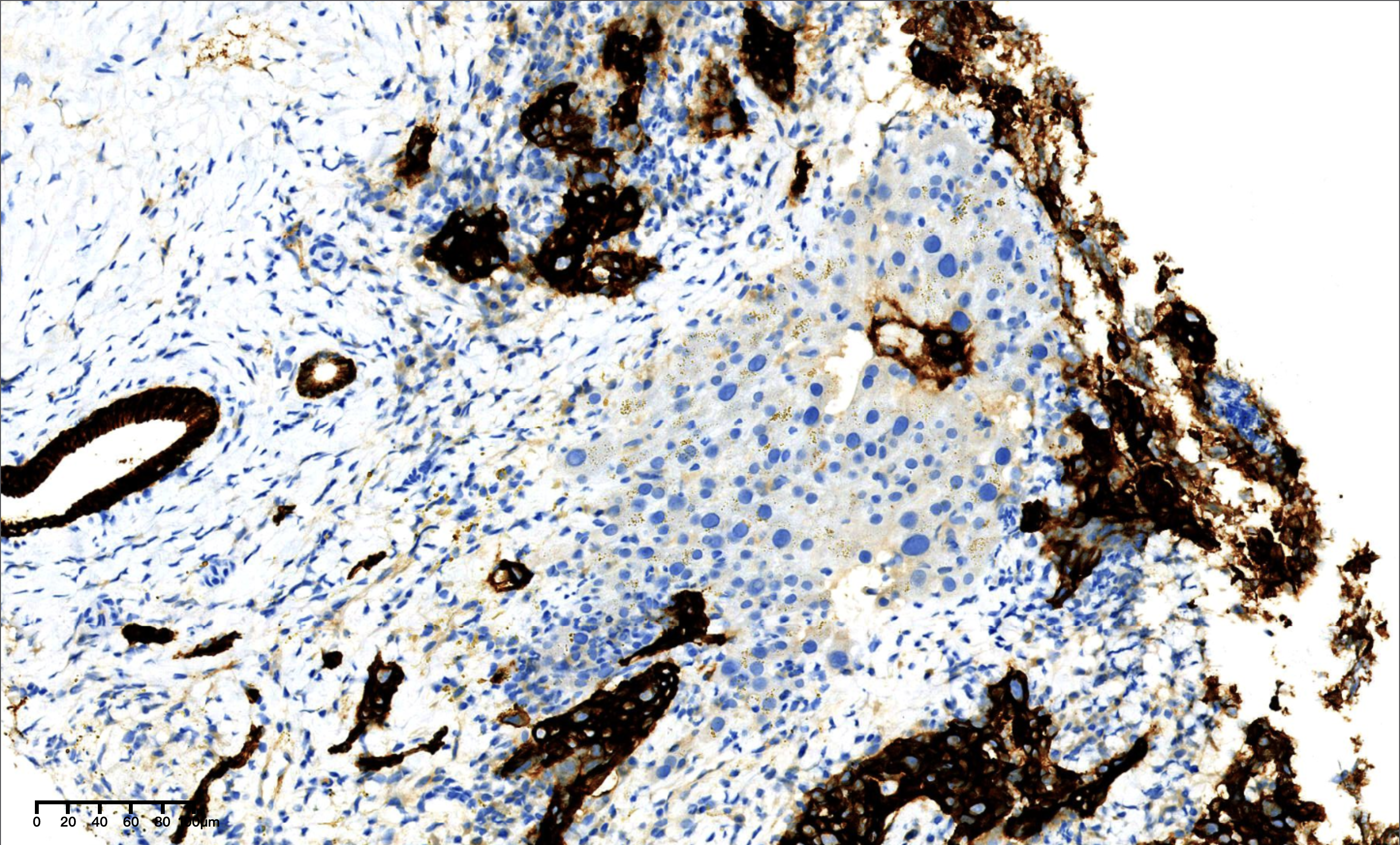

Supplement: Supplementary file 8 — Source data Fig. 6 [file 44321_2024_59_MOESM8_ESM.zip › Figure 6/6H/IHC 100.png]

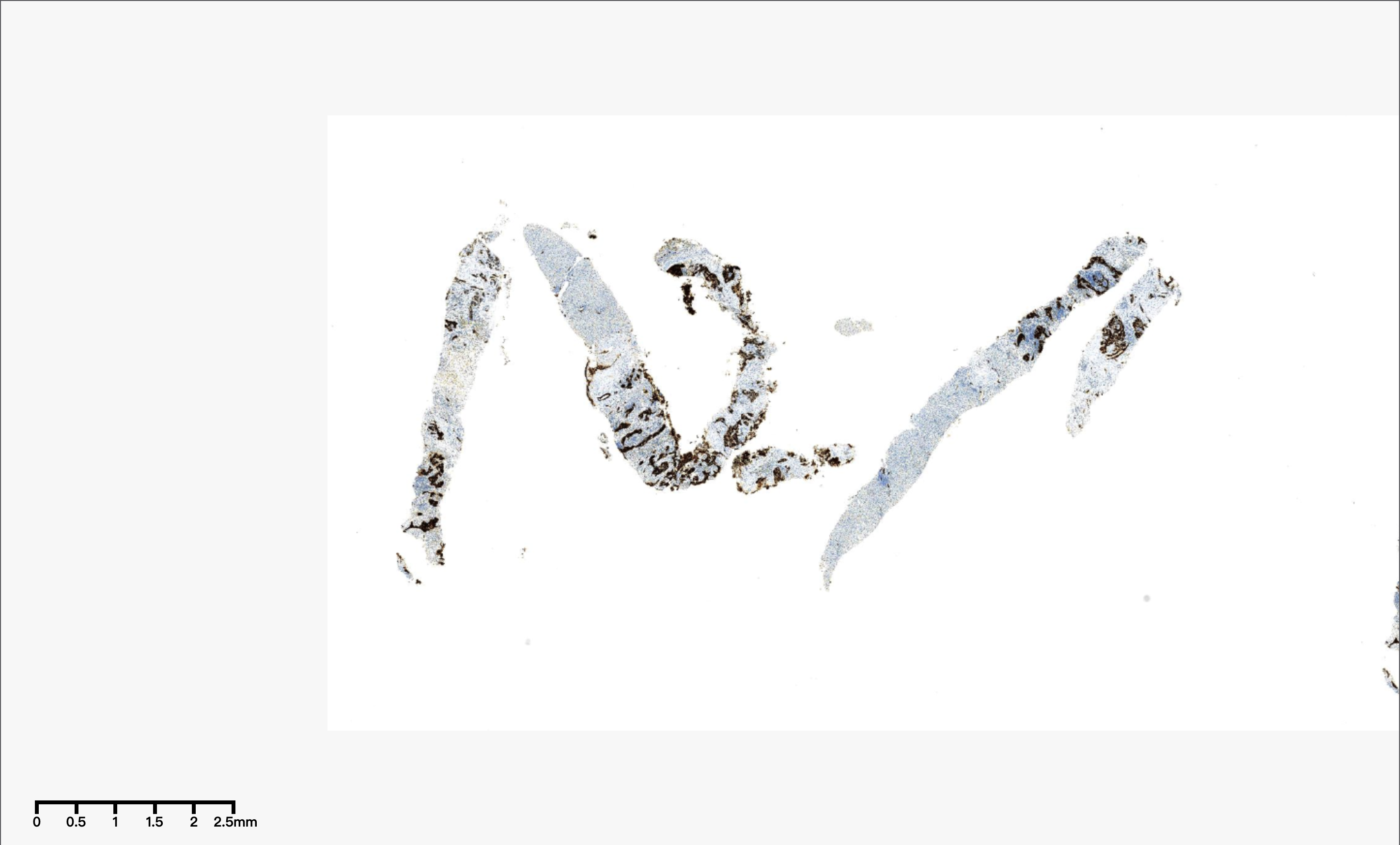

Supplement: Supplementary file 8 — Source data Fig. 6 [file 44321_2024_59_MOESM8_ESM.zip › Figure 6/6H/IHC 2500.png]

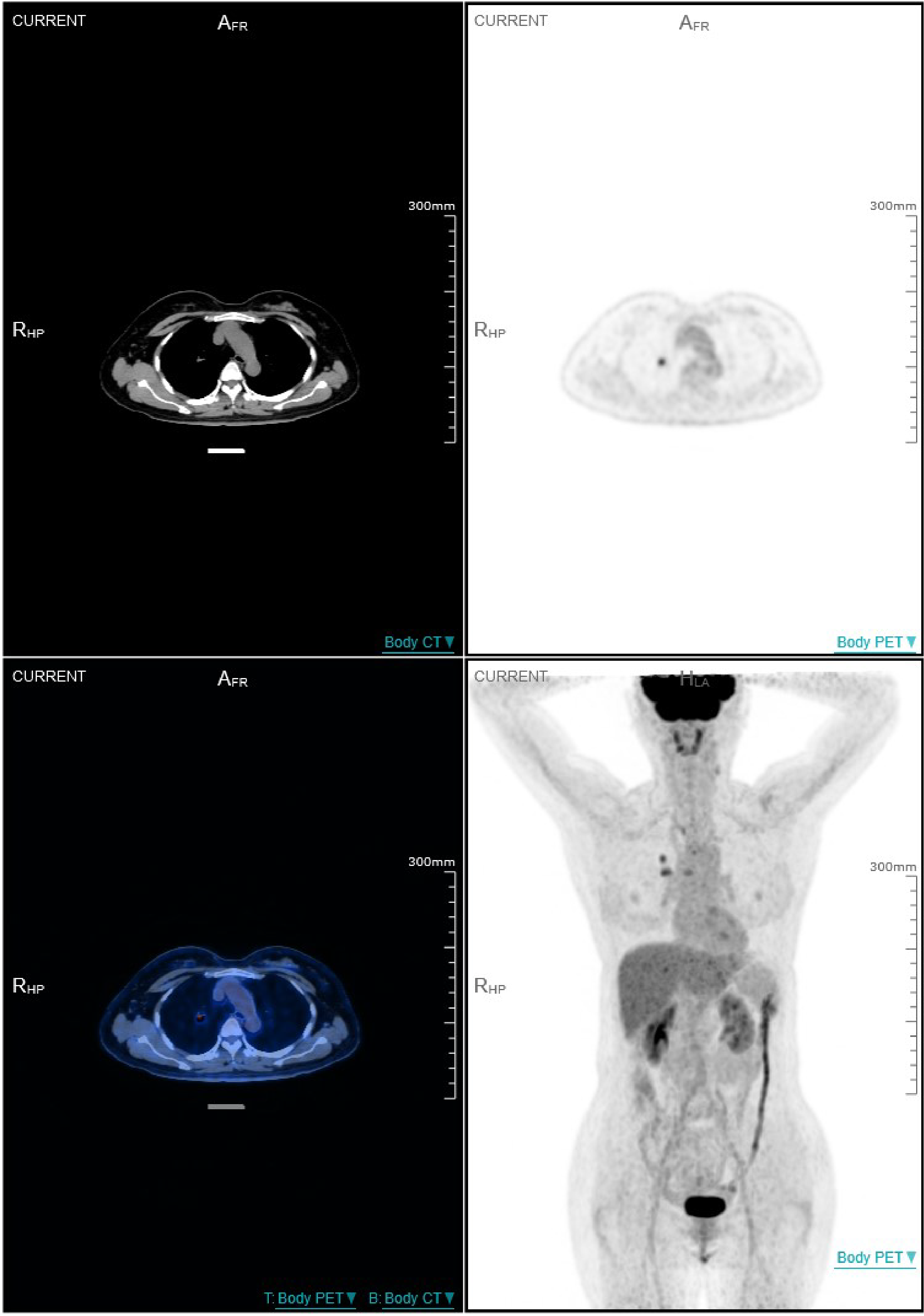

Supplement: Supplementary file 9 — Source data Fig. 7 [file 44321_2024_59_MOESM9_ESM.zip › Figure 7/7A–C/7A.png]

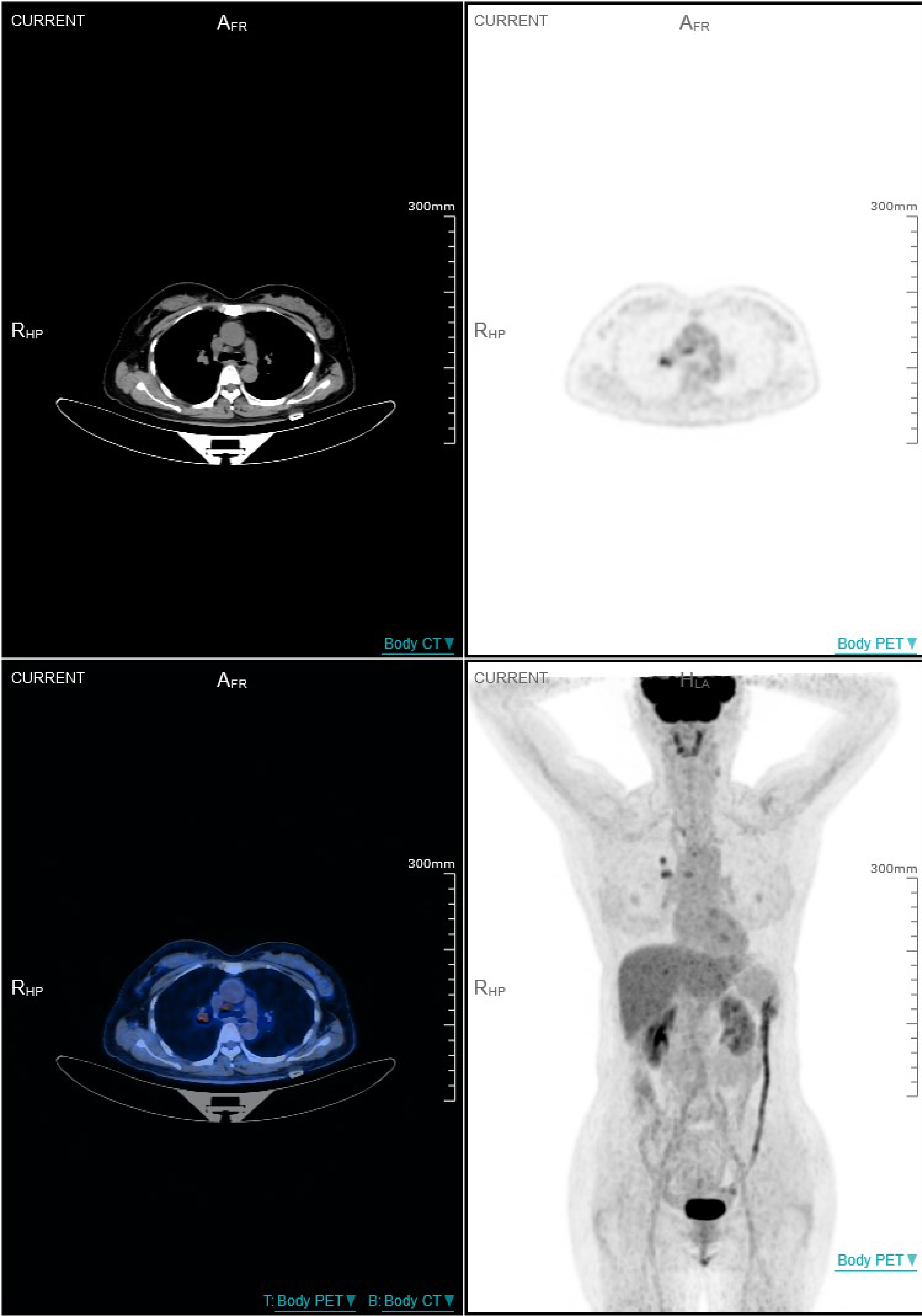

Supplement: Supplementary file 9 — Source data Fig. 7 [file 44321_2024_59_MOESM9_ESM.zip › Figure 7/7A–C/7B.png]

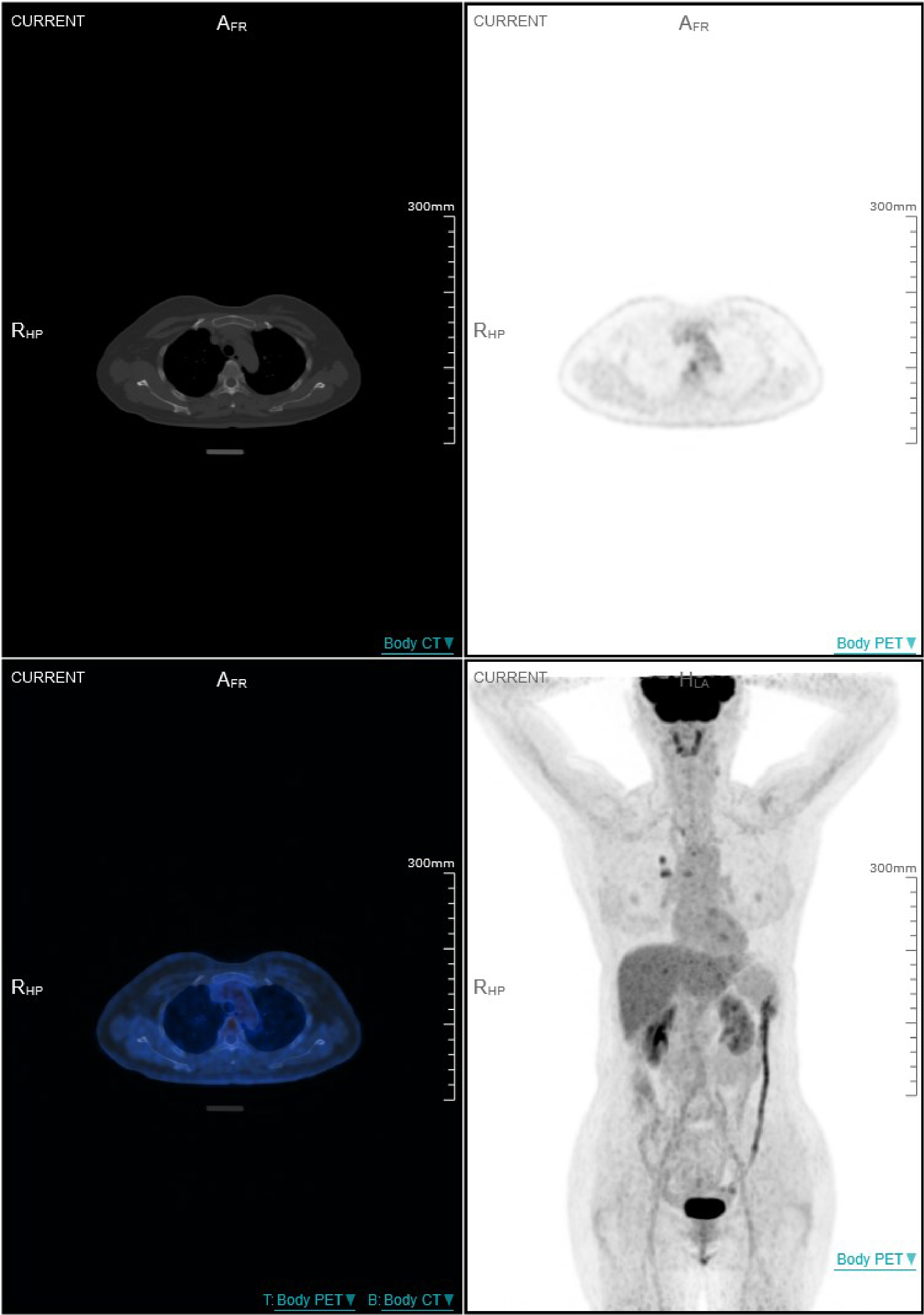

Supplement: Supplementary file 9 — Source data Fig. 7 [file 44321_2024_59_MOESM9_ESM.zip › Figure 7/7A–C/7C.png]

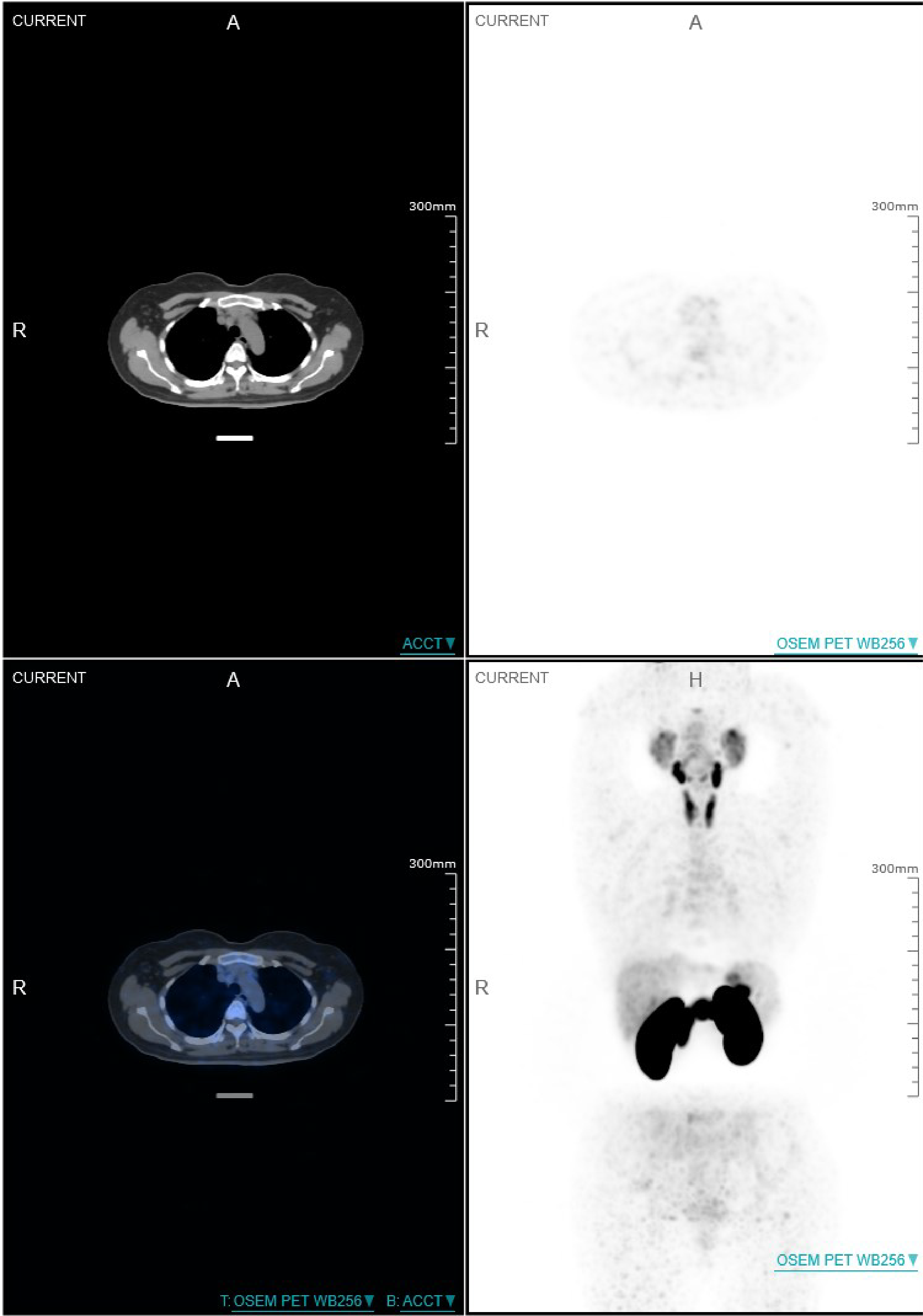

Supplement: Supplementary file 9 — Source data Fig. 7 [file 44321_2024_59_MOESM9_ESM.zip › Figure 7/7D–F/7D.png]

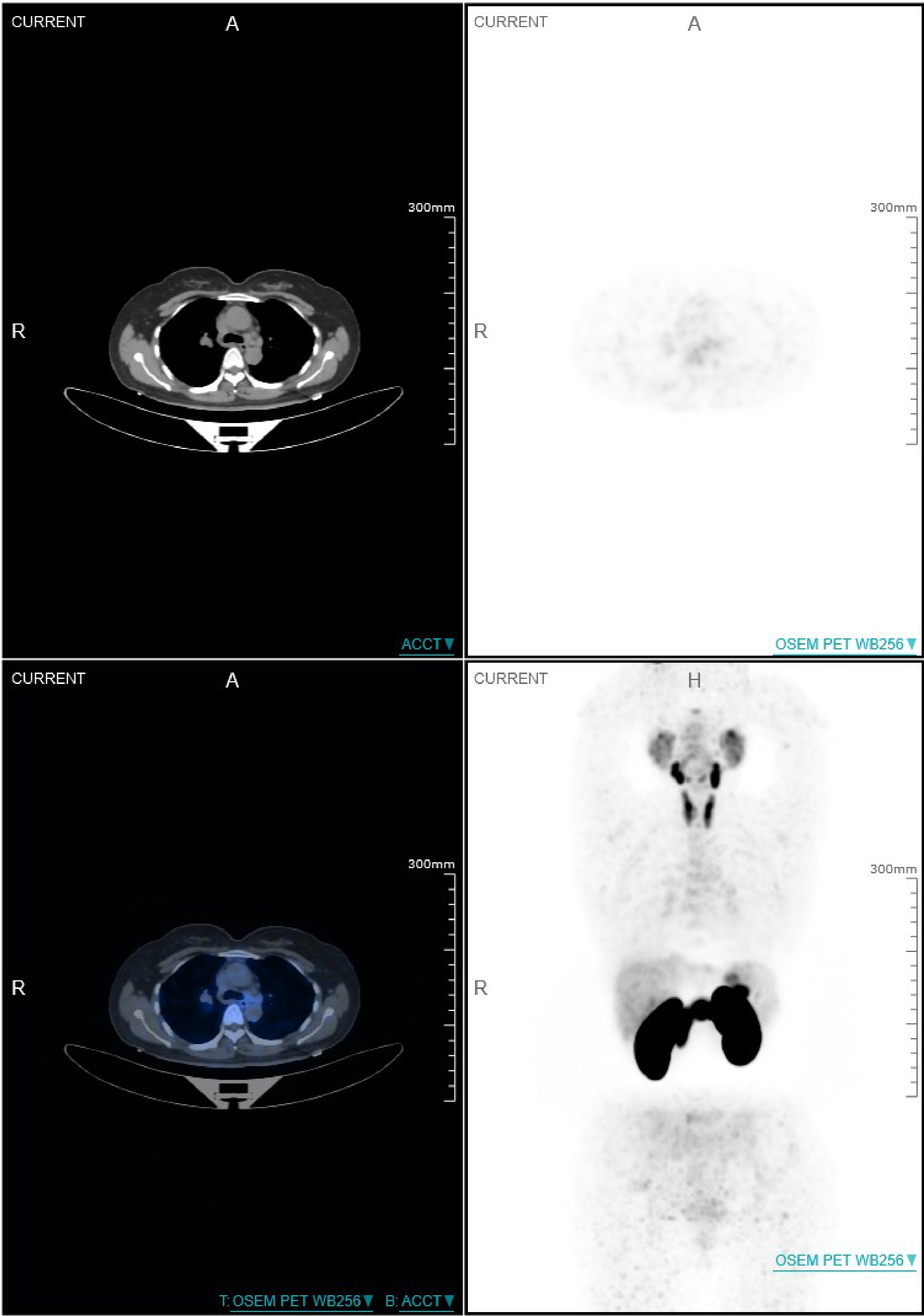

Supplement: Supplementary file 9 — Source data Fig. 7 [file 44321_2024_59_MOESM9_ESM.zip › Figure 7/7D–F/7E.png]

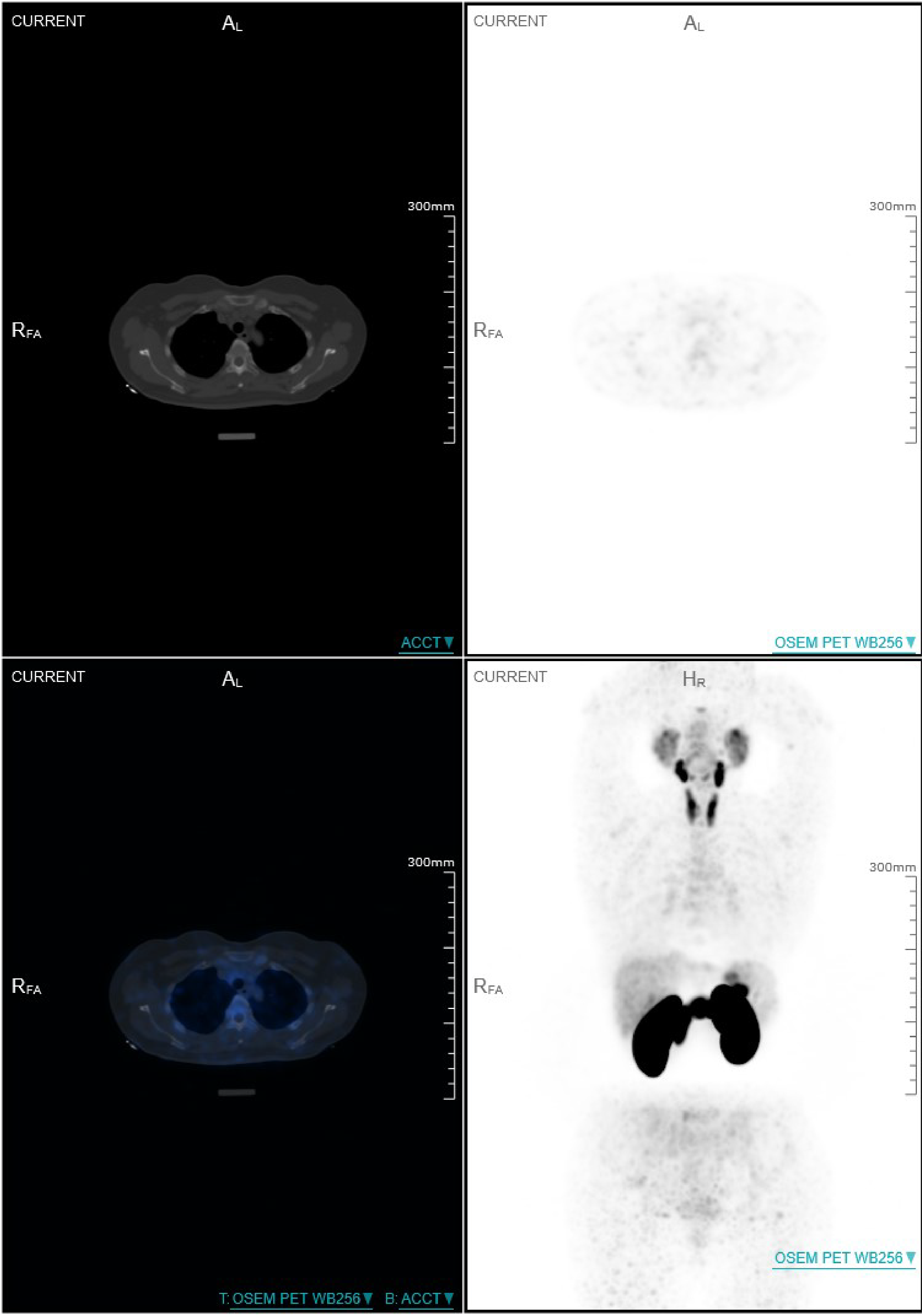

Supplement: Supplementary file 9 — Source data Fig. 7 [file 44321_2024_59_MOESM9_ESM.zip › Figure 7/7D–F/7F.png]

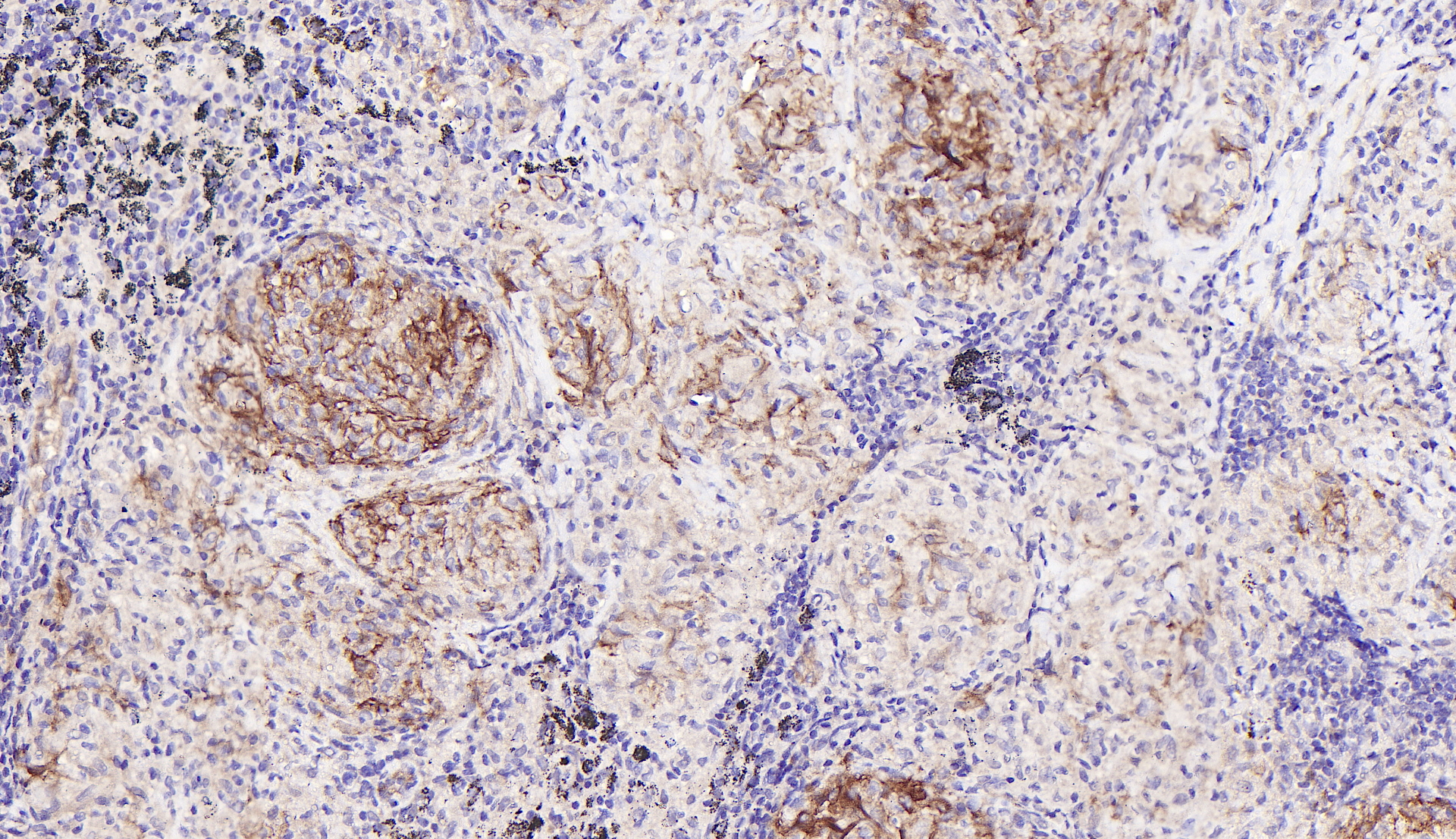

Supplement: Supplementary file 9 — Source data Fig. 7 [file 44321_2024_59_MOESM9_ESM.zip › Figure 7/7G H/7G.jpg]

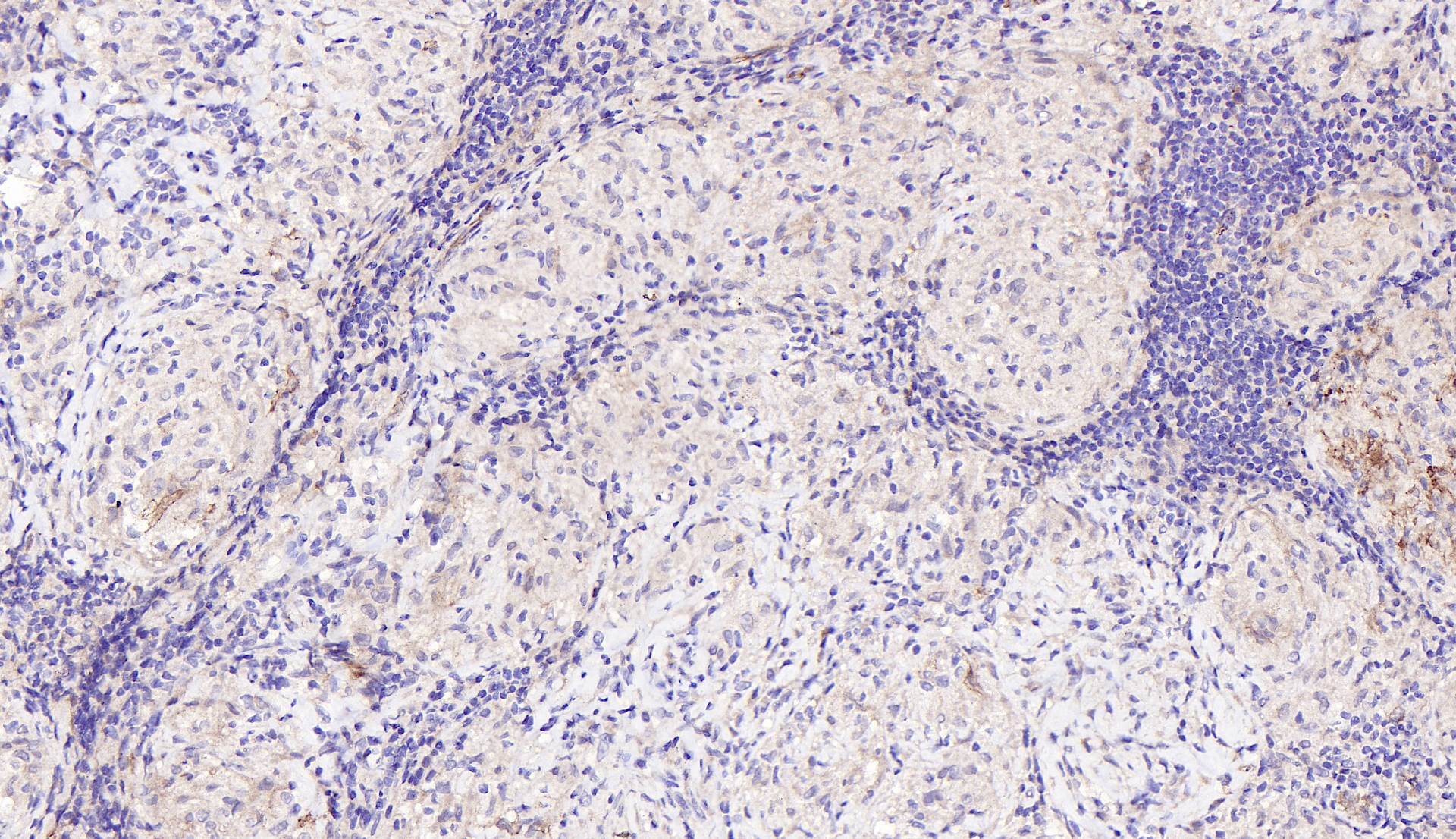

Supplement: Supplementary file 9 — Source data Fig. 7 [file 44321_2024_59_MOESM9_ESM.zip › Figure 7/7G H/7H.jpg]

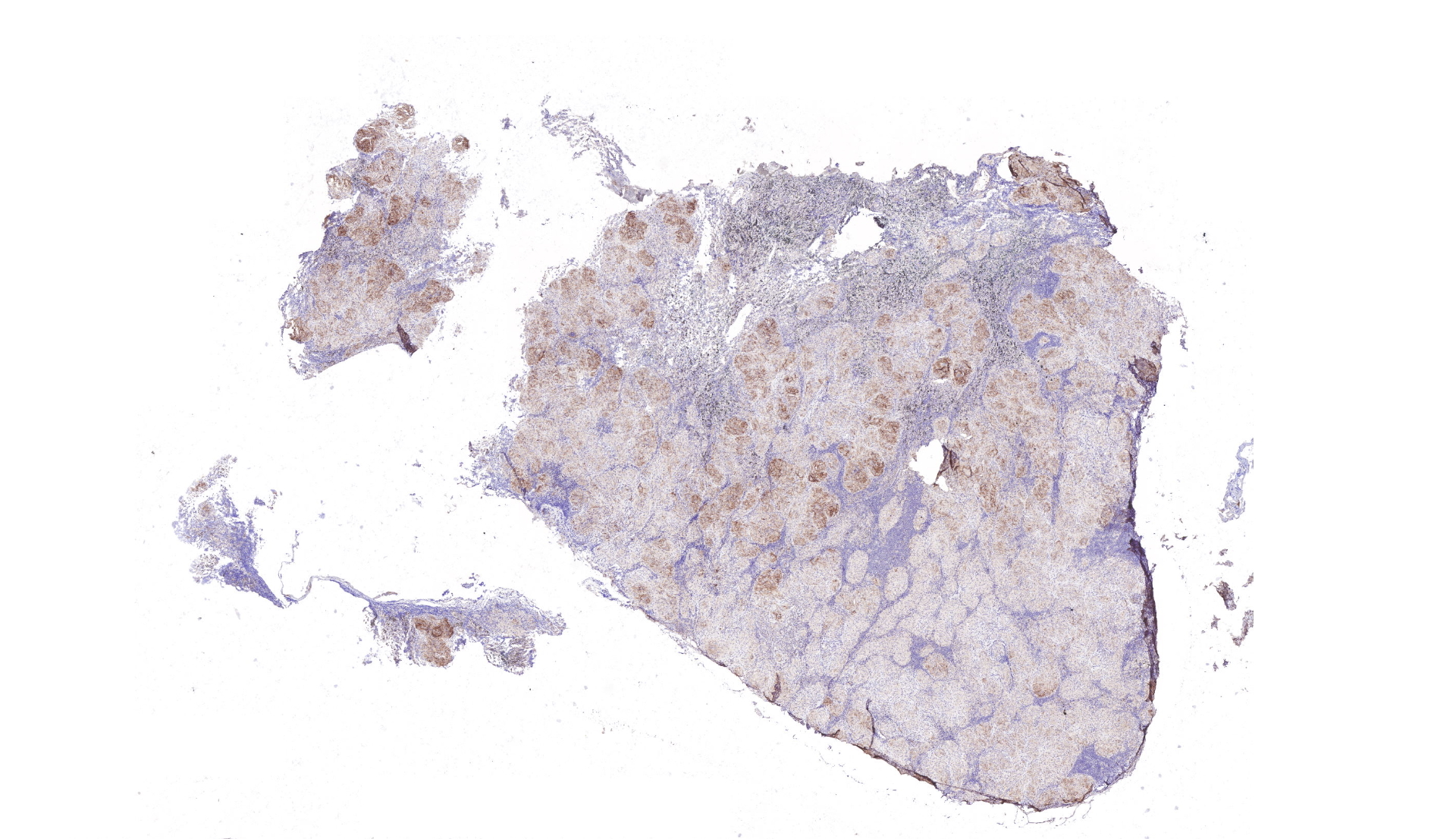

Supplement: Supplementary file 9 — Source data Fig. 7 [file 44321_2024_59_MOESM9_ESM.zip › Figure 7/7G H/hilar lymph node tissue.jpg]

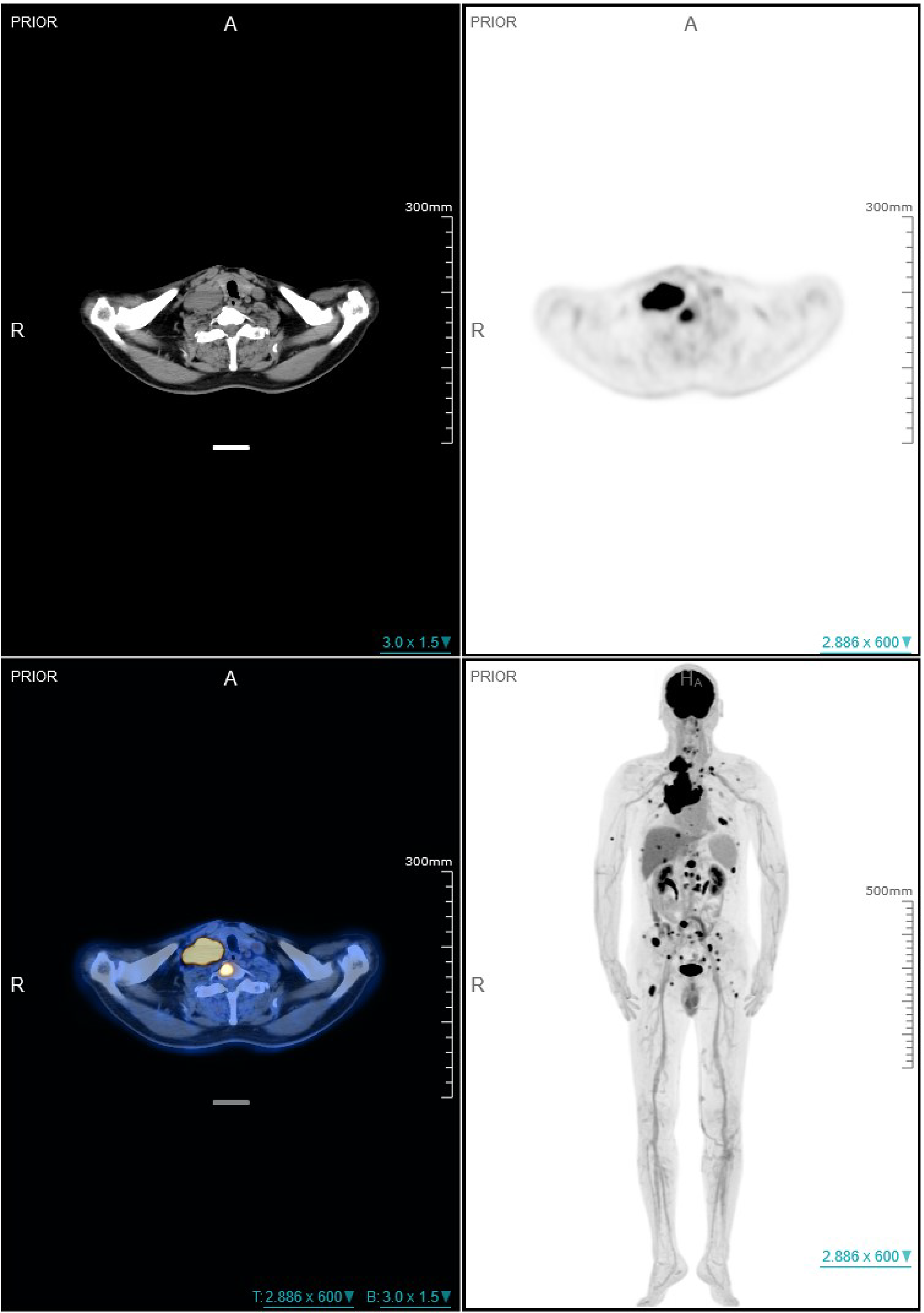

Supplement: Supplementary file 10 — Source data Fig. 8 [file 44321_2024_59_MOESM10_ESM.zip › Figure 8/8A–C/8A.png]

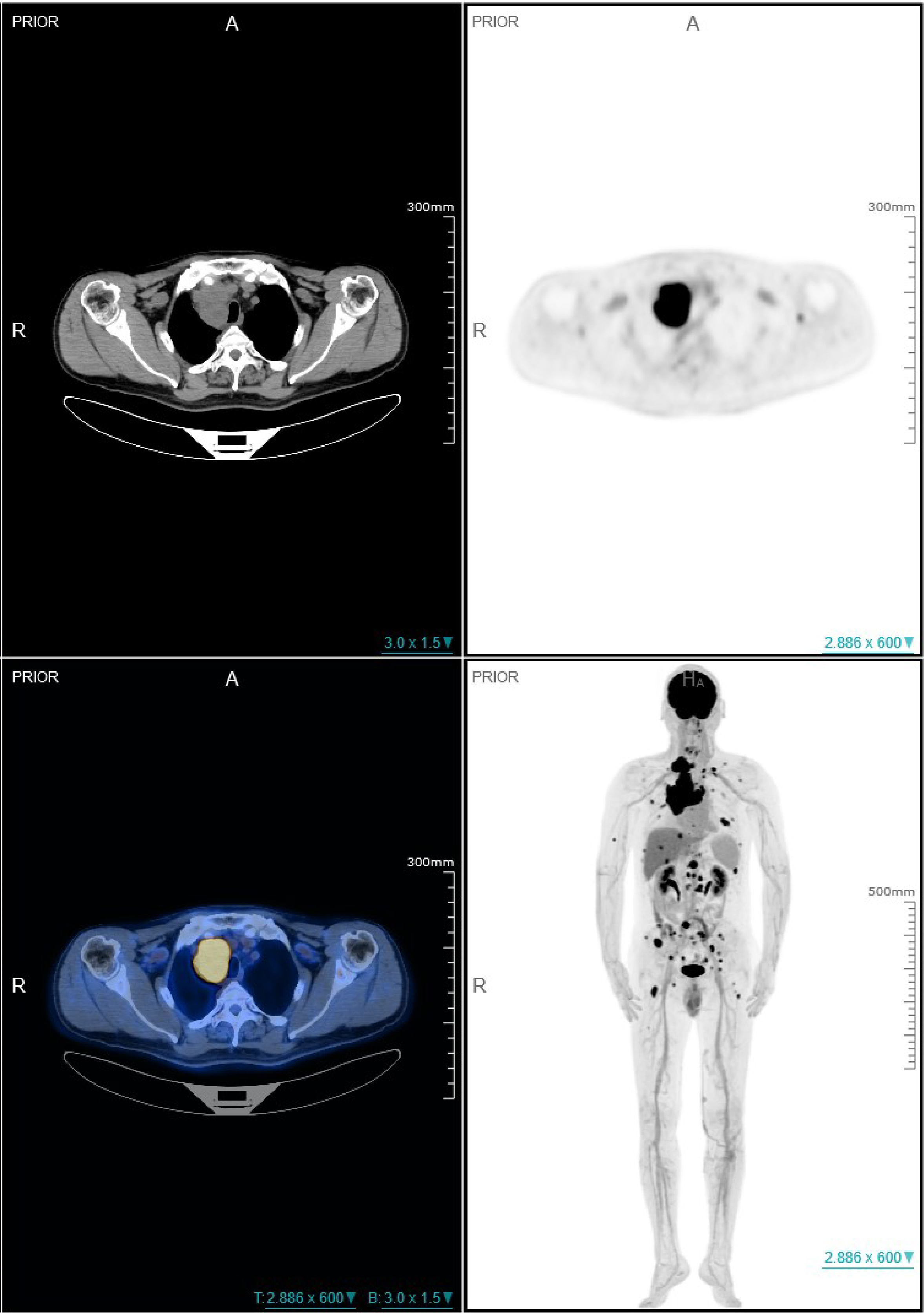

Supplement: Supplementary file 10 — Source data Fig. 8 [file 44321_2024_59_MOESM10_ESM.zip › Figure 8/8A–C/8B.png]

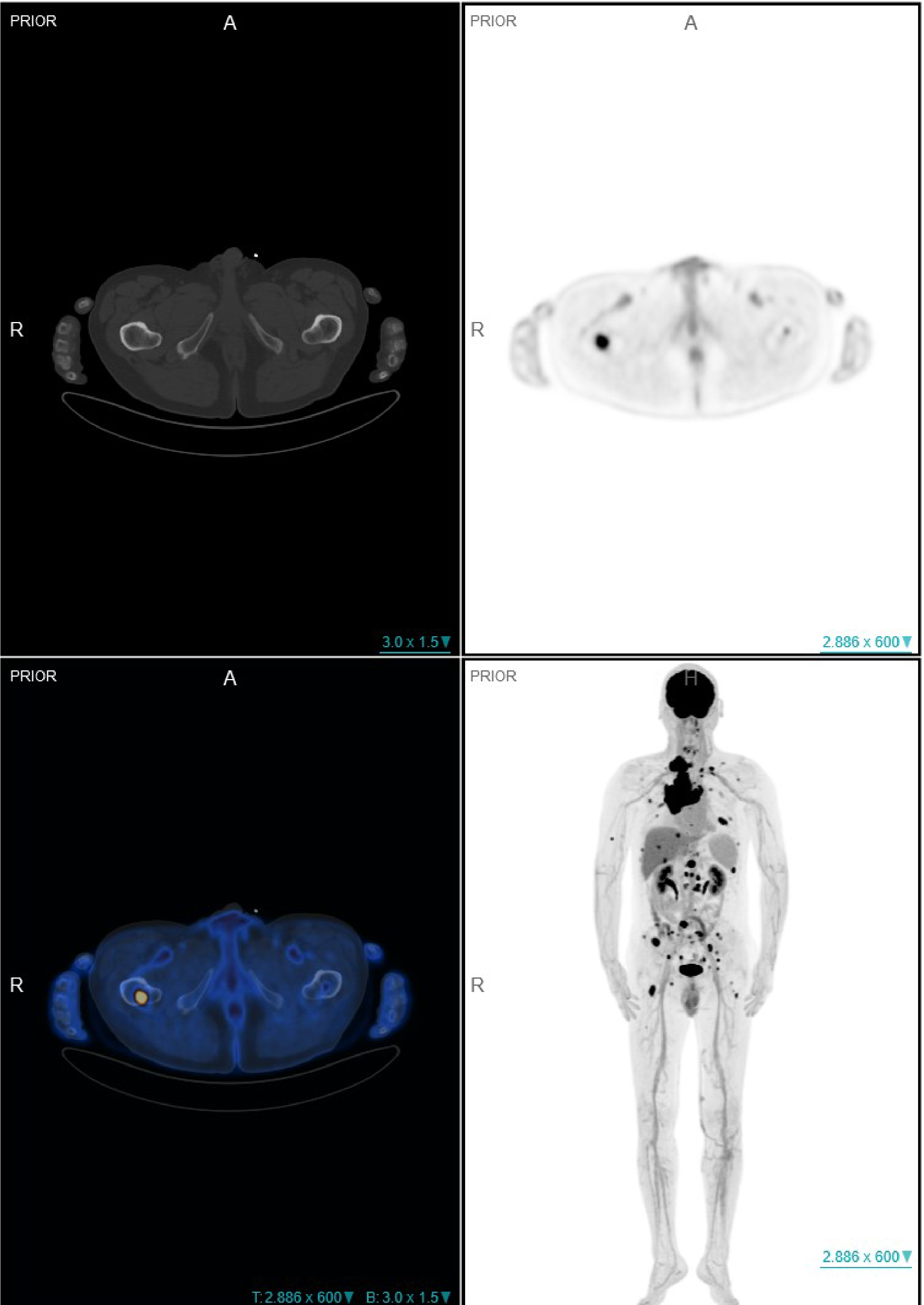

Supplement: Supplementary file 10 — Source data Fig. 8 [file 44321_2024_59_MOESM10_ESM.zip › Figure 8/8A–C/8C.png]

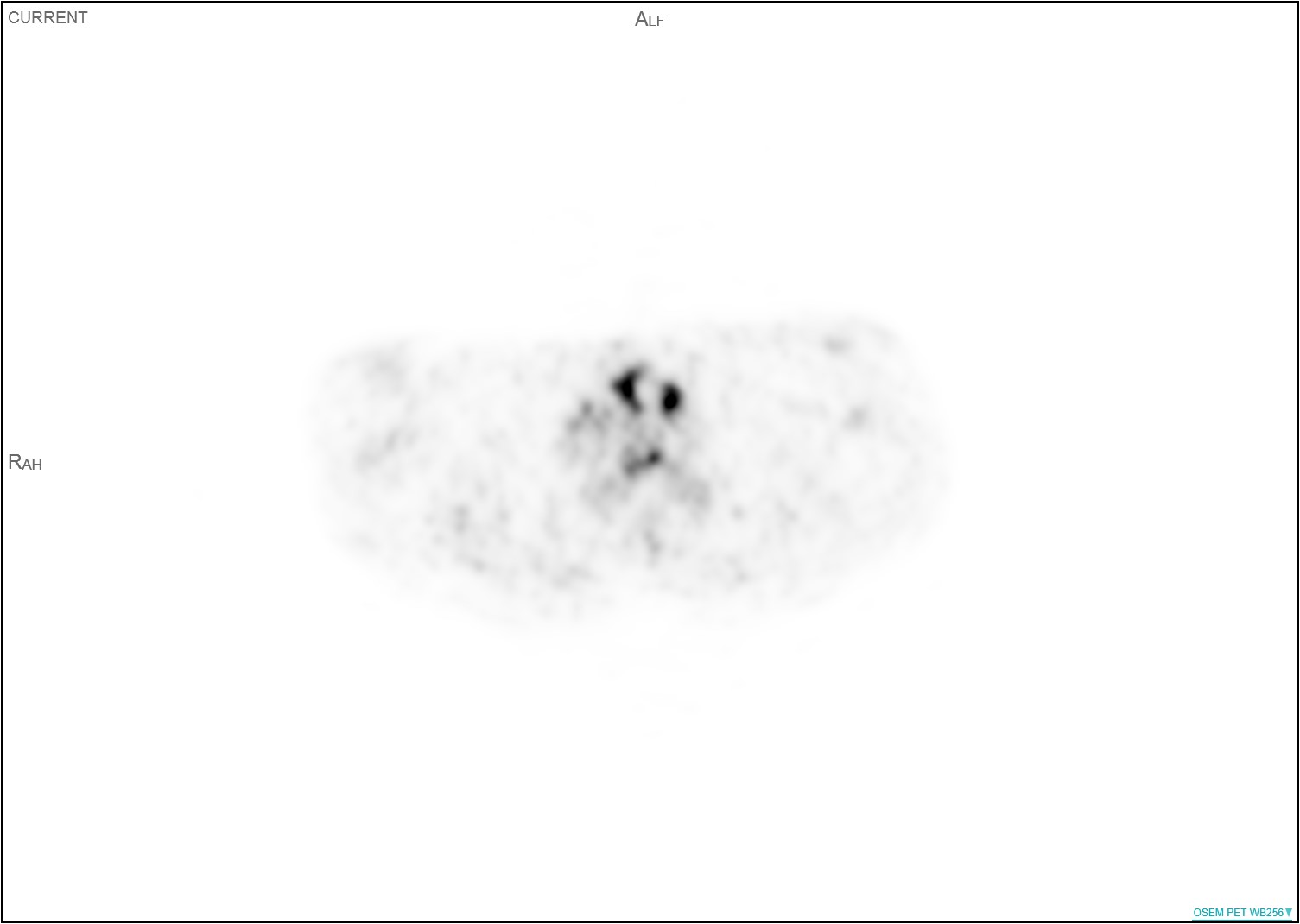

Supplement: Supplementary file 10 — Source data Fig. 8 [file 44321_2024_59_MOESM10_ESM.zip › Figure 8/8D–F/8D PET.jpg]

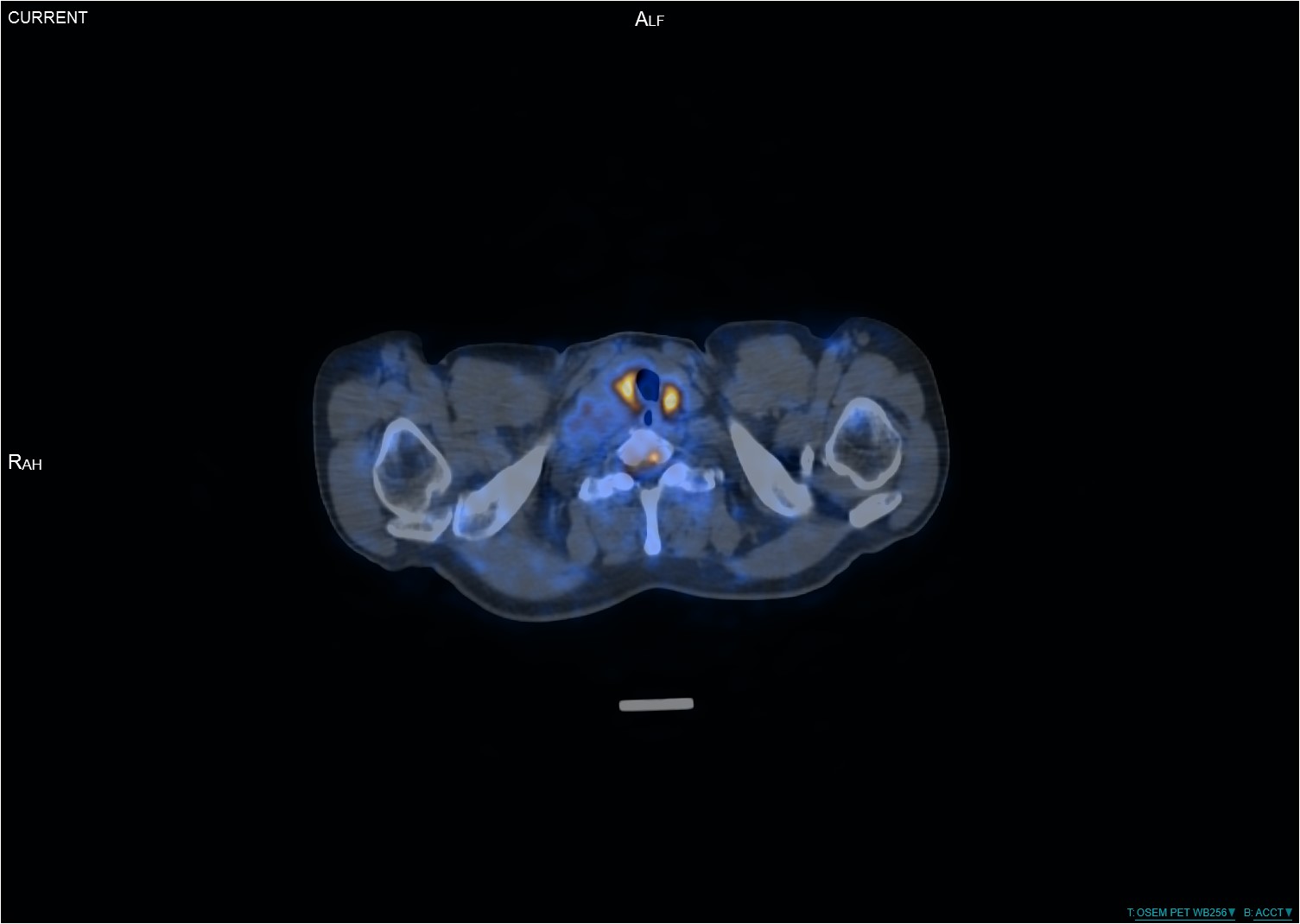

Supplement: Supplementary file 10 — Source data Fig. 8 [file 44321_2024_59_MOESM10_ESM.zip › Figure 8/8D–F/8D PET_CT.jpg]

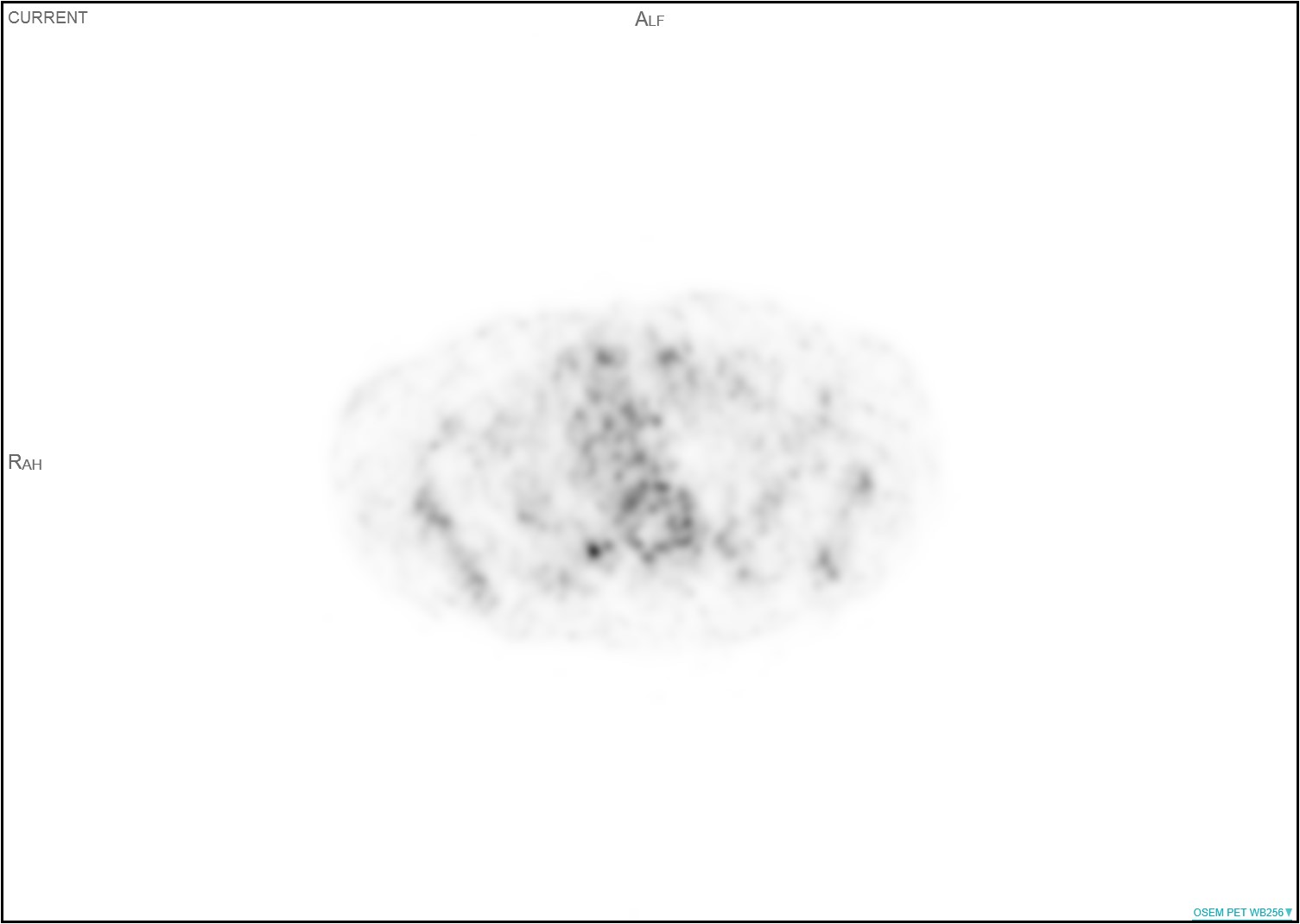

Supplement: Supplementary file 10 — Source data Fig. 8 [file 44321_2024_59_MOESM10_ESM.zip › Figure 8/8D–F/8E PET.jpg]

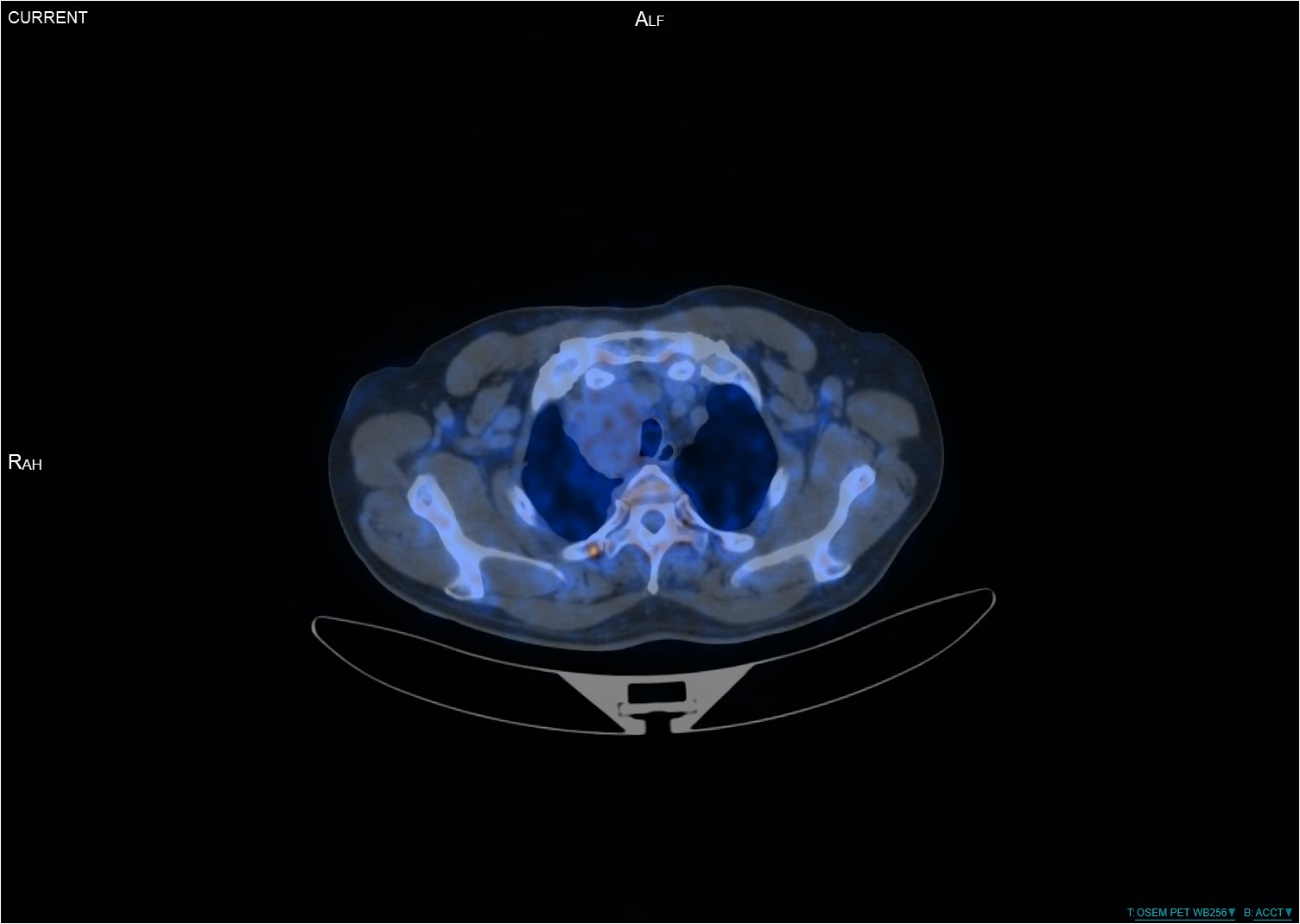

Supplement: Supplementary file 10 — Source data Fig. 8 [file 44321_2024_59_MOESM10_ESM.zip › Figure 8/8D–F/8E PET_CT.jpg]

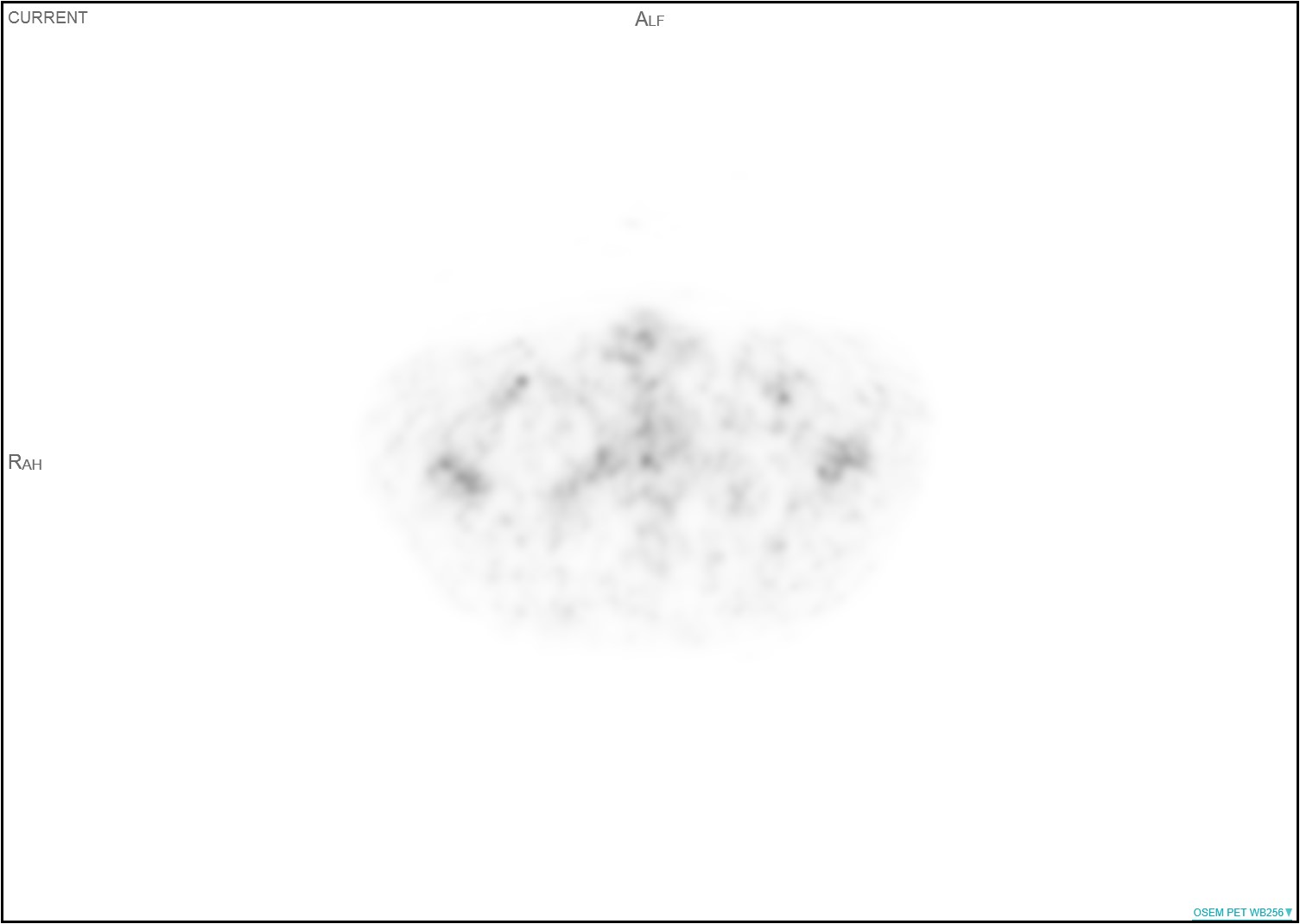

Supplement: Supplementary file 10 — Source data Fig. 8 [file 44321_2024_59_MOESM10_ESM.zip › Figure 8/8D–F/8F PET.jpg]

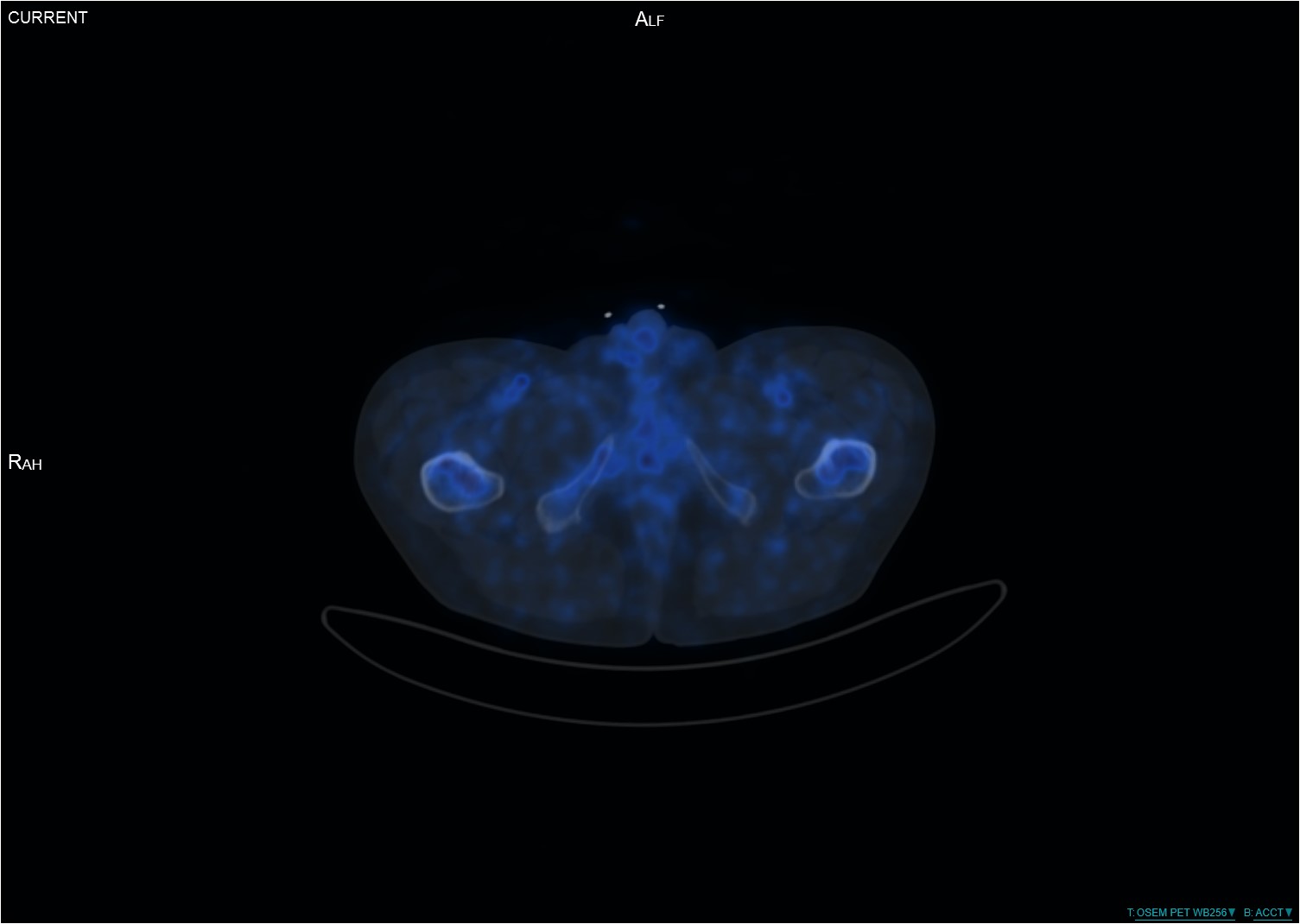

Supplement: Supplementary file 10 — Source data Fig. 8 [file 44321_2024_59_MOESM10_ESM.zip › Figure 8/8D–F/8F PET_CT.jpg]

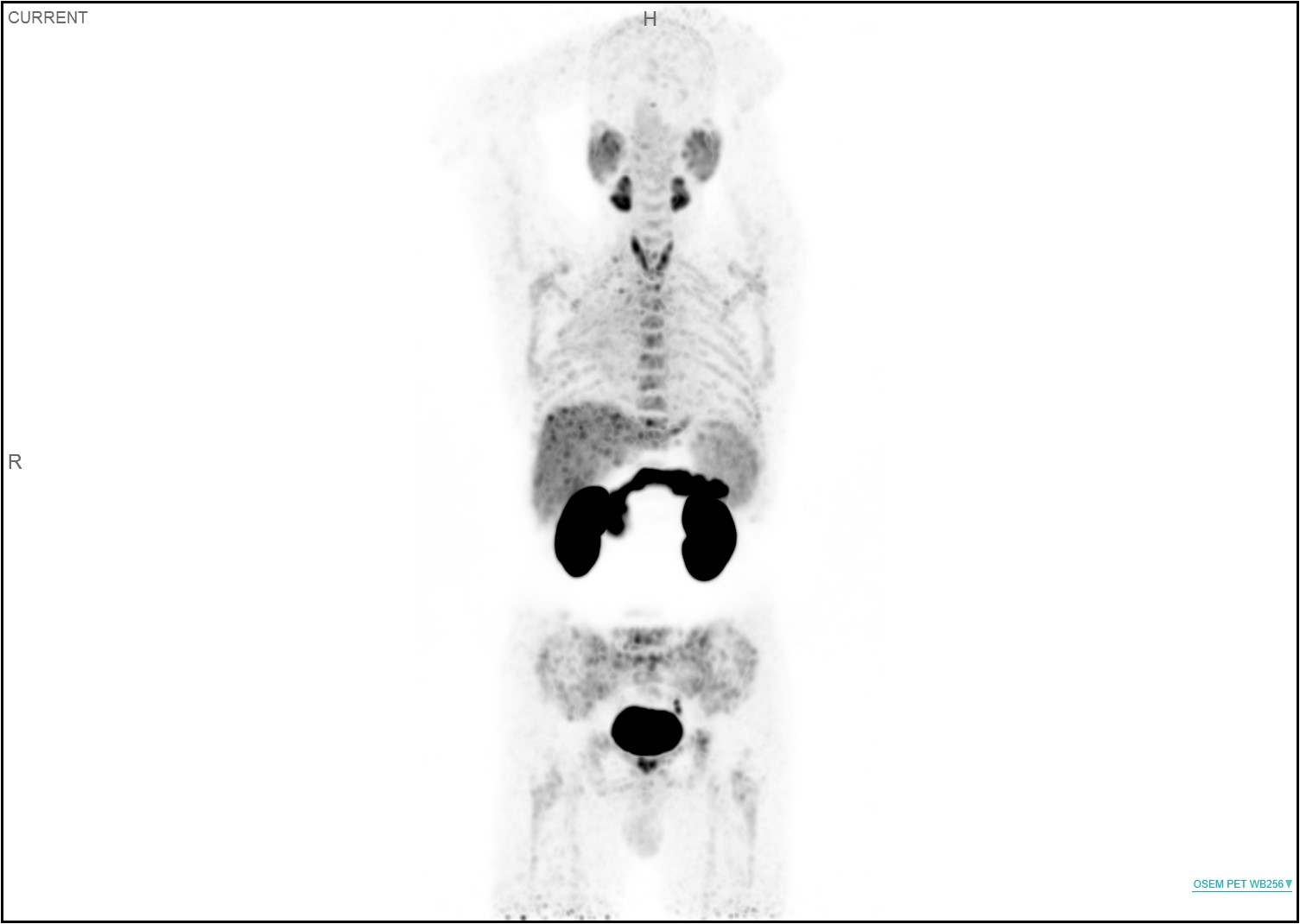

Supplement: Supplementary file 10 — Source data Fig. 8 [file 44321_2024_59_MOESM10_ESM.zip › Figure 8/8D–F/MIP.jpg]

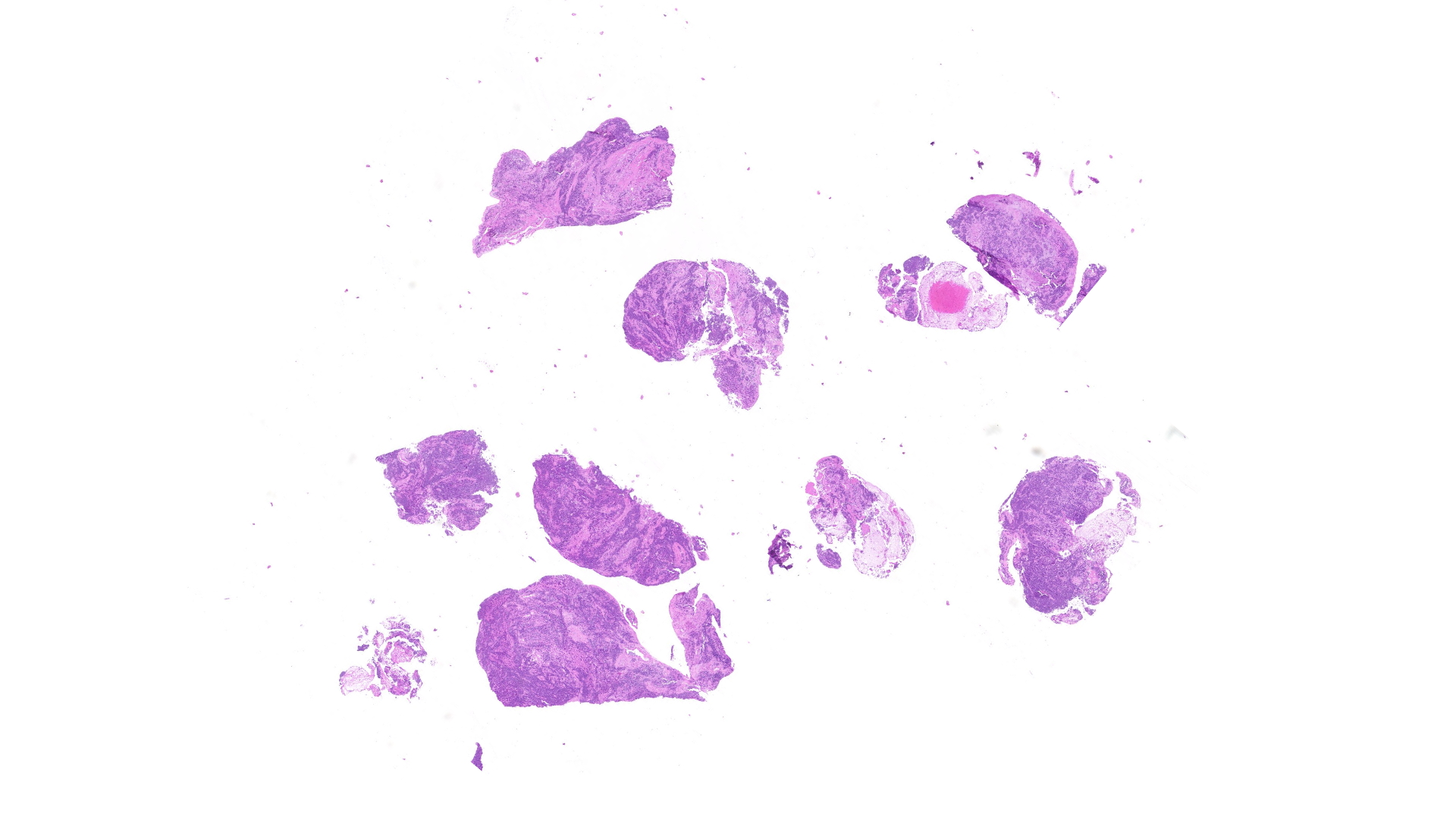

Supplement: Supplementary file 10 — Source data Fig. 8 [file 44321_2024_59_MOESM10_ESM.zip › Figure 8/8G/H&E_1000.jpg]

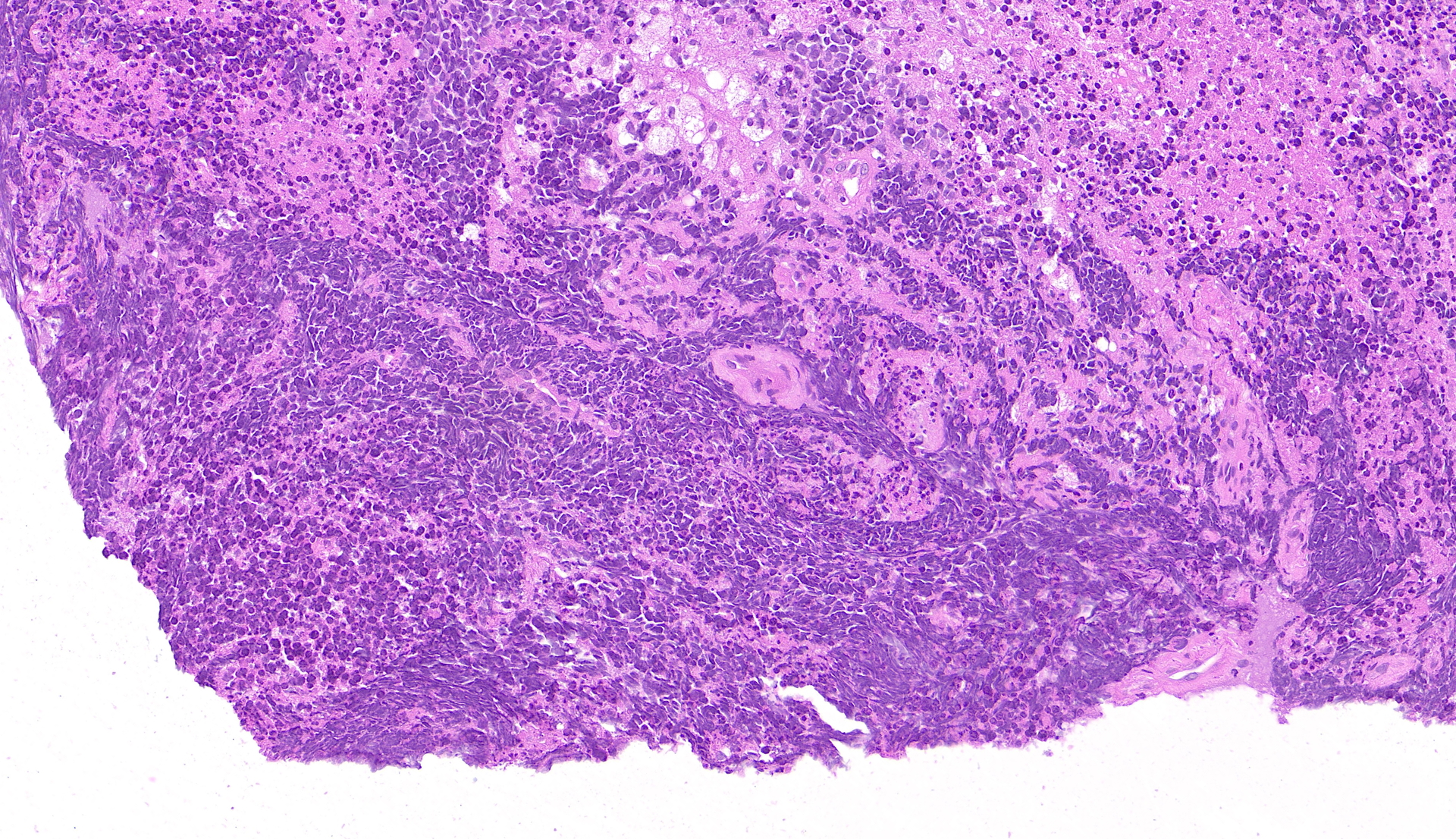

Supplement: Supplementary file 10 — Source data Fig. 8 [file 44321_2024_59_MOESM10_ESM.zip › Figure 8/8G/H&E_200.jpg]

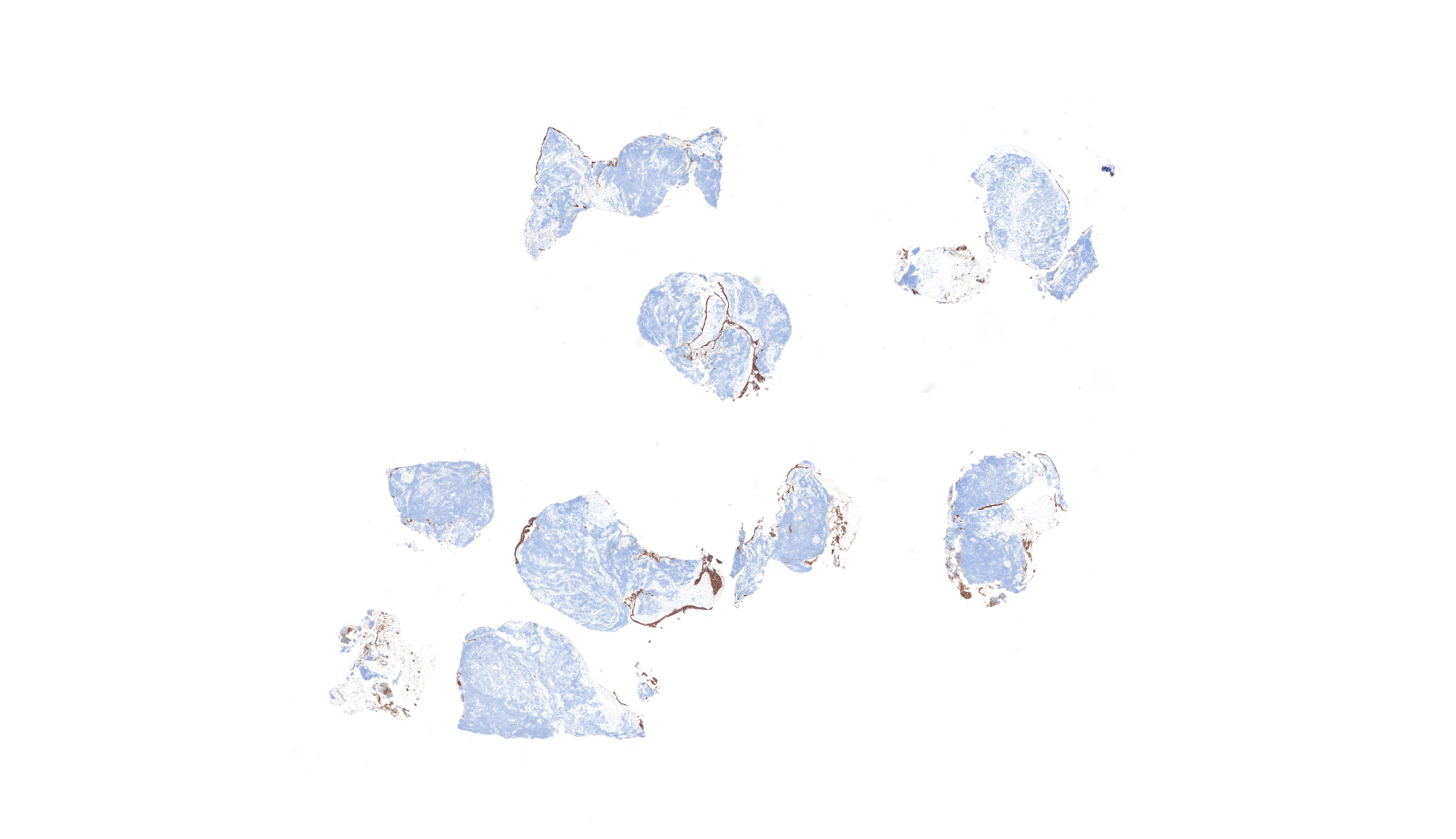

Supplement: Supplementary file 10 — Source data Fig. 8 [file 44321_2024_59_MOESM10_ESM.zip › Figure 8/8H/TROP2_1000.jpg]

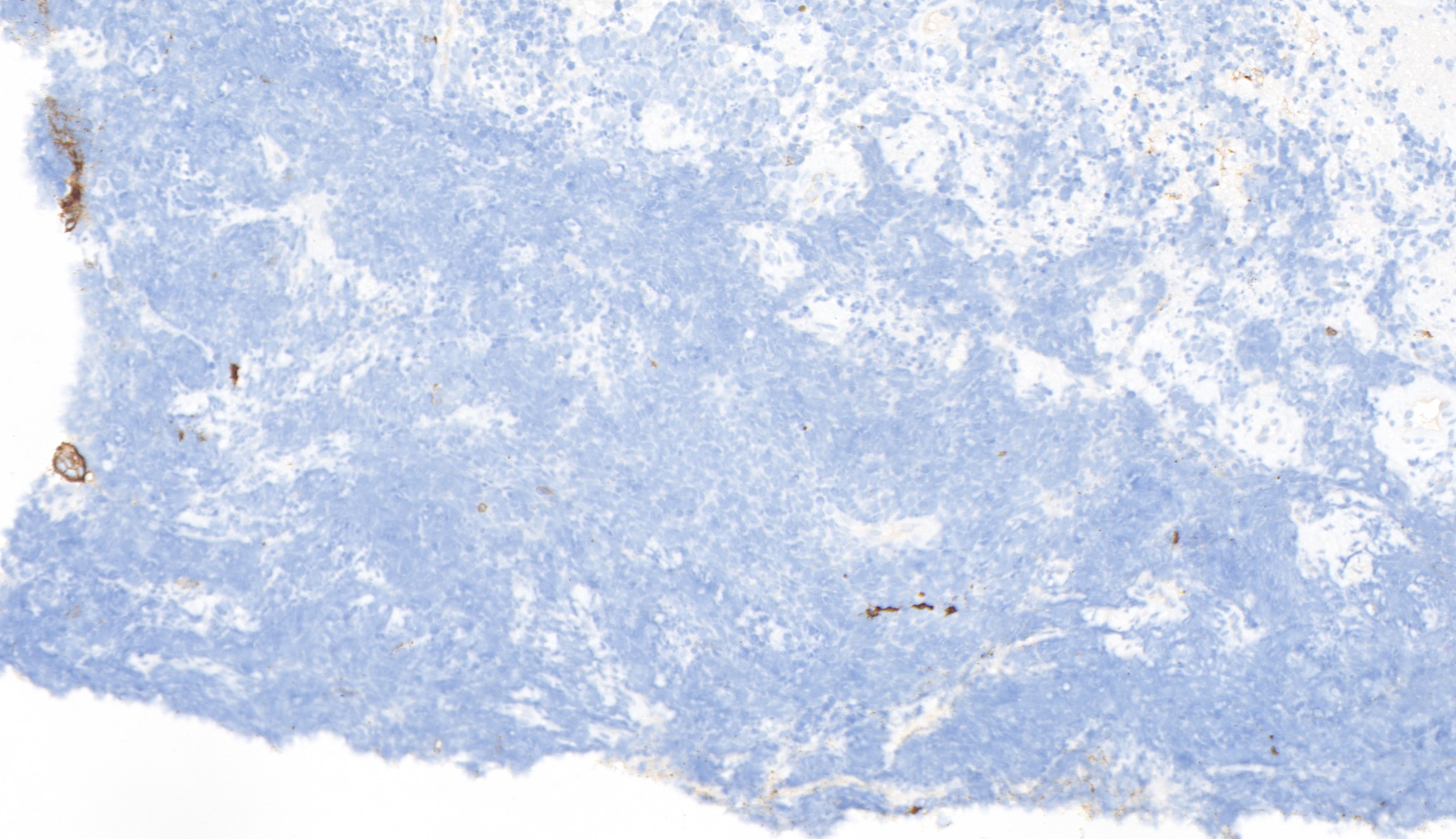

Supplement: Supplementary file 10 — Source data Fig. 8 [file 44321_2024_59_MOESM10_ESM.zip › Figure 8/8H/TROP2_200.jpg]

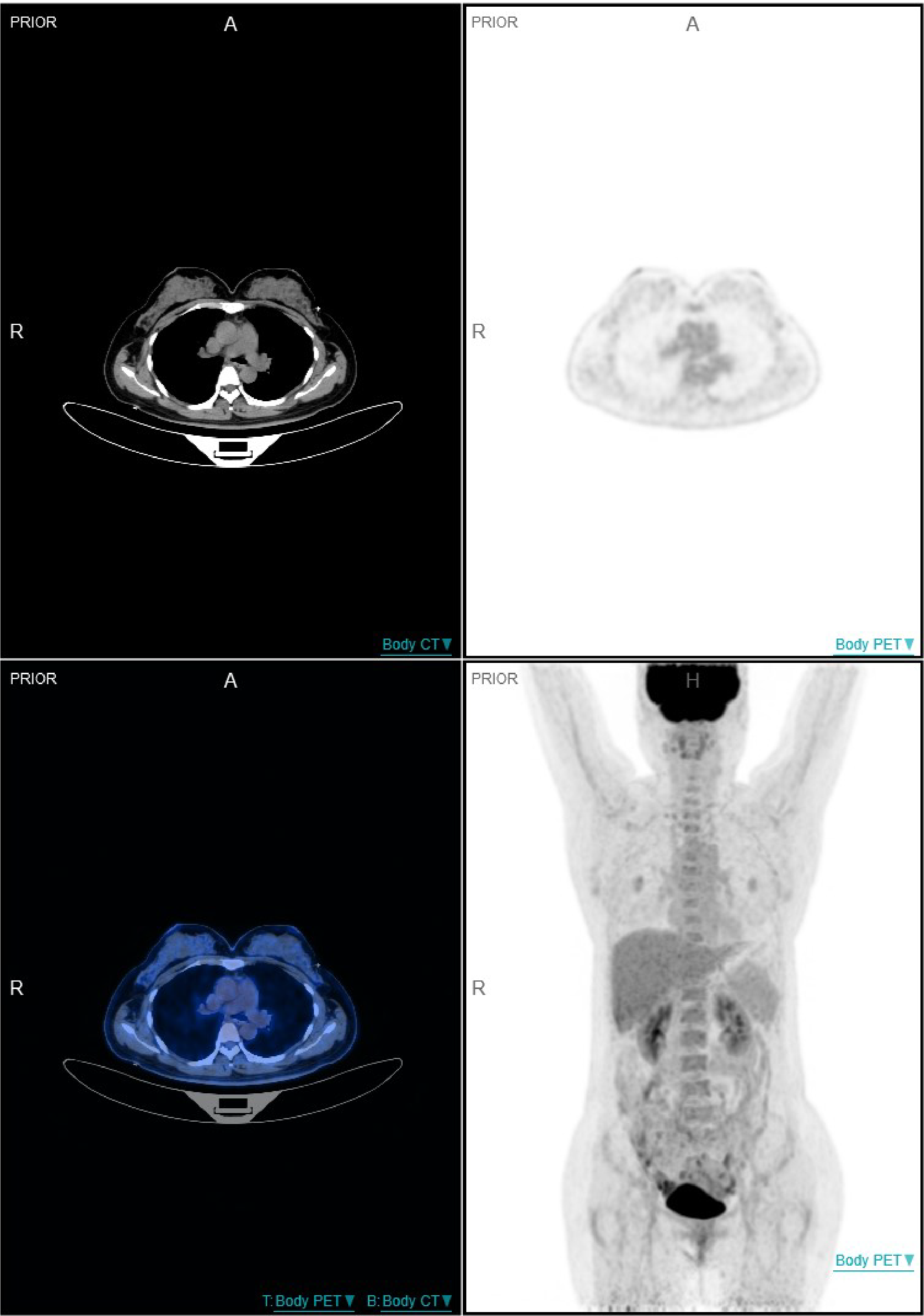

Supplement: Supplementary file 11 — Appendix and EV Figure Source Data [file 44321_2024_59_MOESM11_ESM.zip › Figure EV4 Source Data/blue arrows.png]

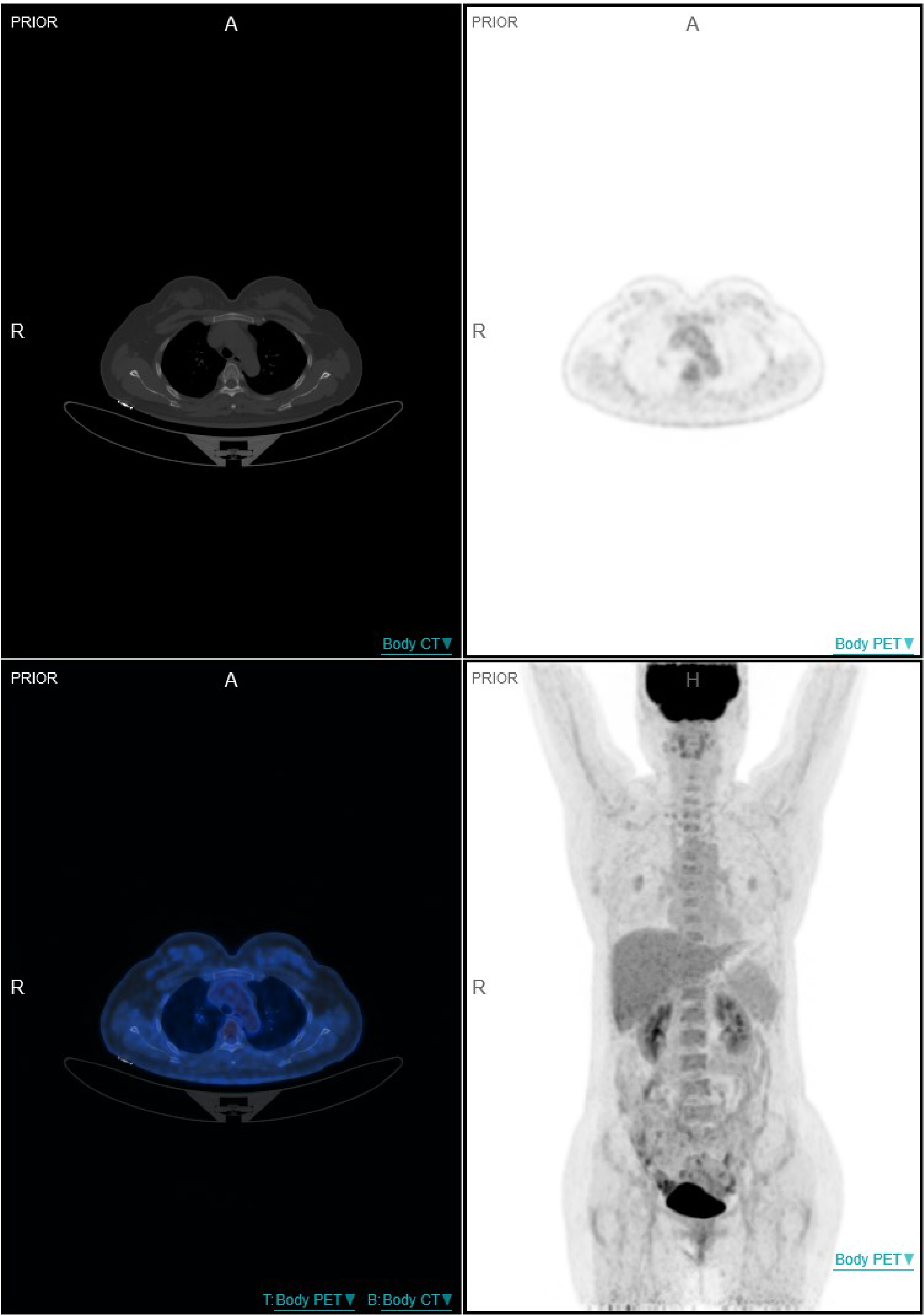

Supplement: Supplementary file 11 — Appendix and EV Figure Source Data [file 44321_2024_59_MOESM11_ESM.zip › Figure EV4 Source Data/green arrows.png]

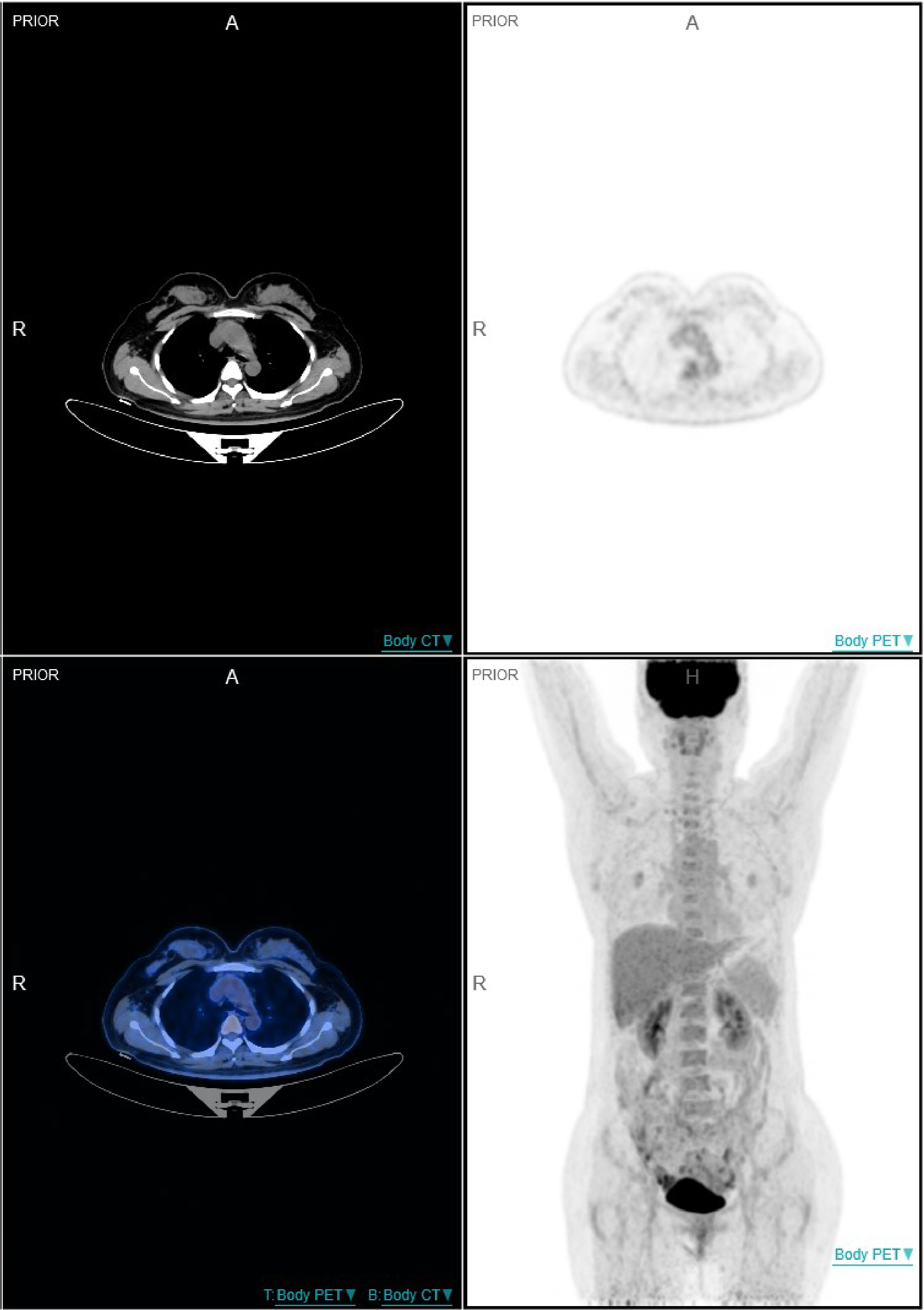

Supplement: Supplementary file 11 — Appendix and EV Figure Source Data [file 44321_2024_59_MOESM11_ESM.zip › Figure EV4 Source Data/orange arrows.png]

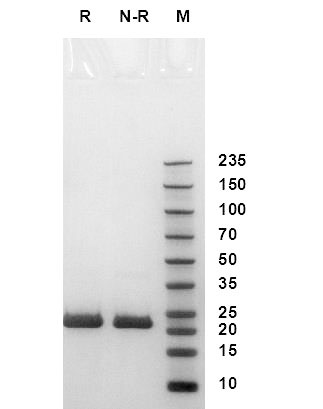

Supplement: Supplementary file 11 — Appendix and EV Figure Source Data [file 44321_2024_59_MOESM11_ESM.zip › Appendix Figure 2A Source Data/ABDT4.jpg]
