# Supplementary material for: ImmunoPET imaging of Trop2 in patients with solid tumours
Source: EMBO Mol Med. 2024 Apr 2;16(5):6. doi: 10.1038/s44321-024-00059-5 (PMC11099157; doi:10.1038/s44321-024-00059-5)
Supplement: Supplementary file 13 — Expanded View Figures [file 44321_2024_59_MOESM13_ESM.pdf]

Expanded View Figures

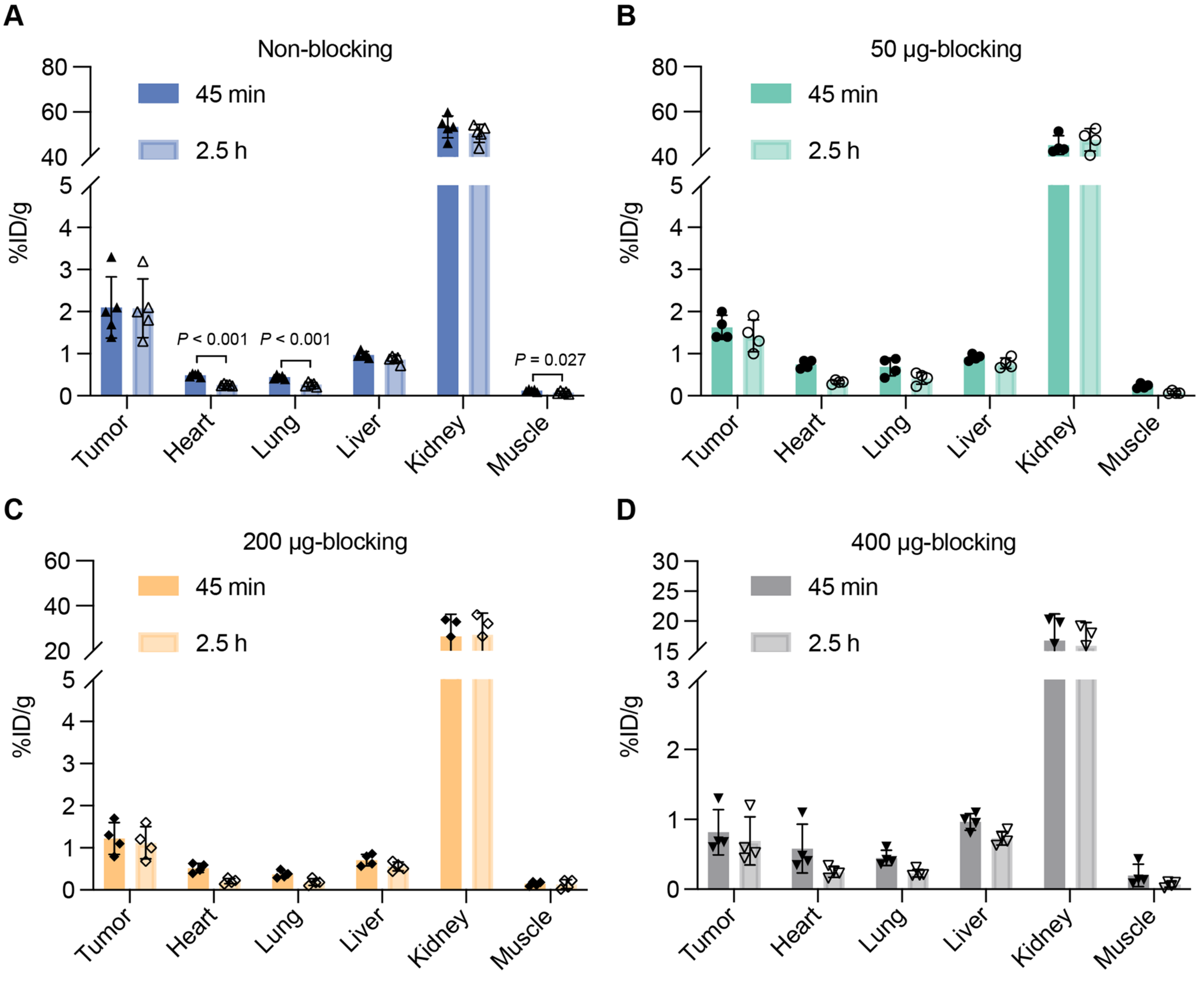

Figure EV1. [^{68}Ga]Ga-NOTA-T4 uptake in the non-blocking and three blocking groups.

Comparison of radioactivity uptake in tumour and major organs at two time points (45 min and 2.5 h) in the non-blocking group (**A,** $n = 5$), 50 µg-blocking (**B**, $n = 6$), 200 µg-blocking (**C**, $n = 6$), and 400 µg-blocking groups (**D**, $n = 6$). *t* test, mean ratio ± SD.

© The Author(s)

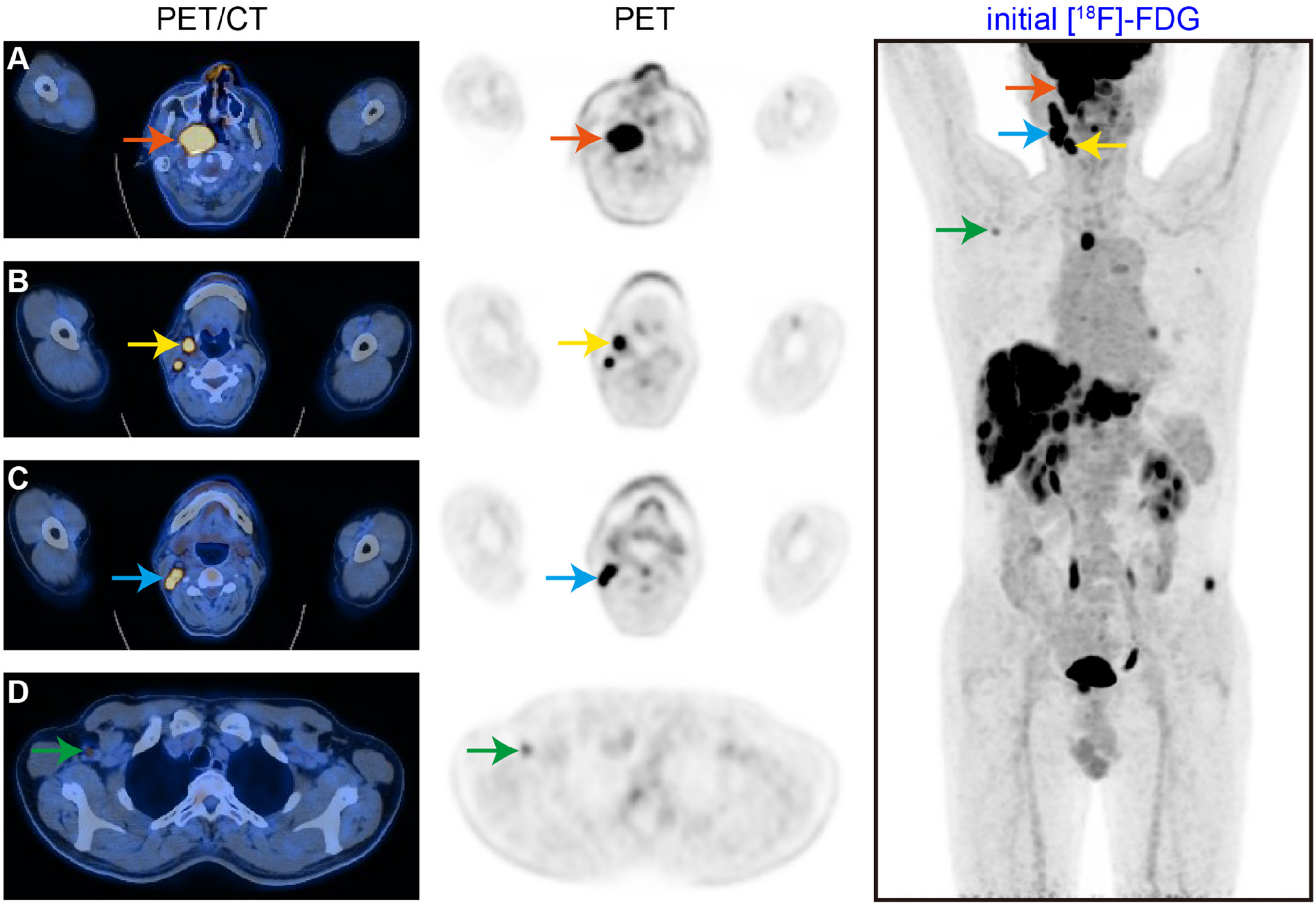

Figure EV2. The initial baseline [^{18}F]-FDG PET/CT examination of patient 1 with nasopharyngeal carcinoma.

The primary tumour (**A**, orange arrows) and multiple metastases, including the right parapharyngeal space lymph node (**B**, yellow arrows), right neck lymph node (**C**, blue arrows), bone and liver metastases (Fig. 6A–C in the main manuscript), and a suspicious right axillary lymph node metastasis (**D**, green arrows) were presented. Note: the [^{18}F]-FDG MIP image here is the same MIP image in Fig. 6.

 © The Author(s)

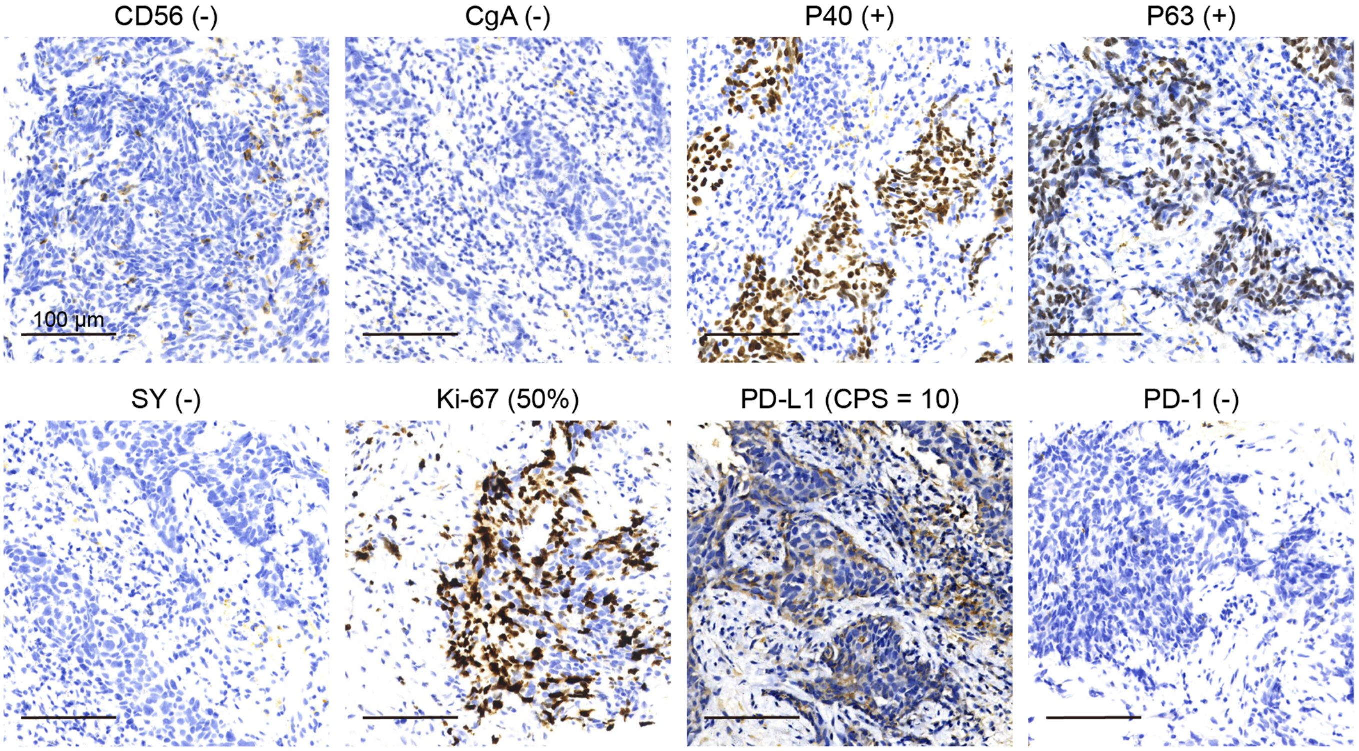

Figure EV3. Histopathological examination of the biopsied liver nodule.

The tested biomarkers confirmed the liver metastasis from poorly differentiated nasopharyngeal carcinoma. "+" means positive expression, and "-" means negative expression. Scale bar: 100 µm.

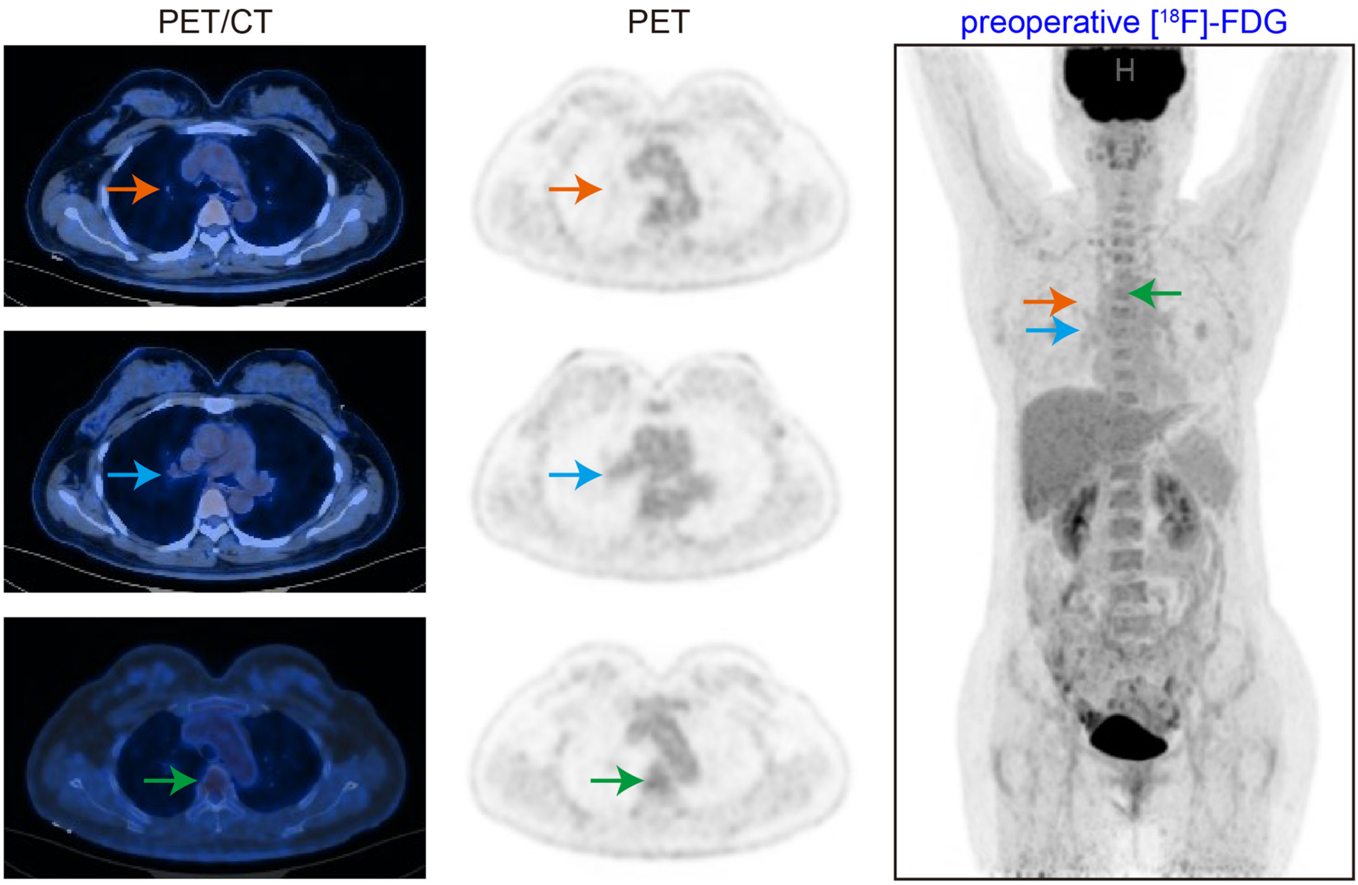

Figure EV4. The preoperative [^{18}F]-FDG PET/CT examination of patient 2.

The lesions and metastasis with enlarged uptake detected on the second [^{18}F]-FDG PET/CT were absent on the first preoperative [^{18}F]-FDG PET/CT. Source data are available online for this figure.

 © The Author(s)

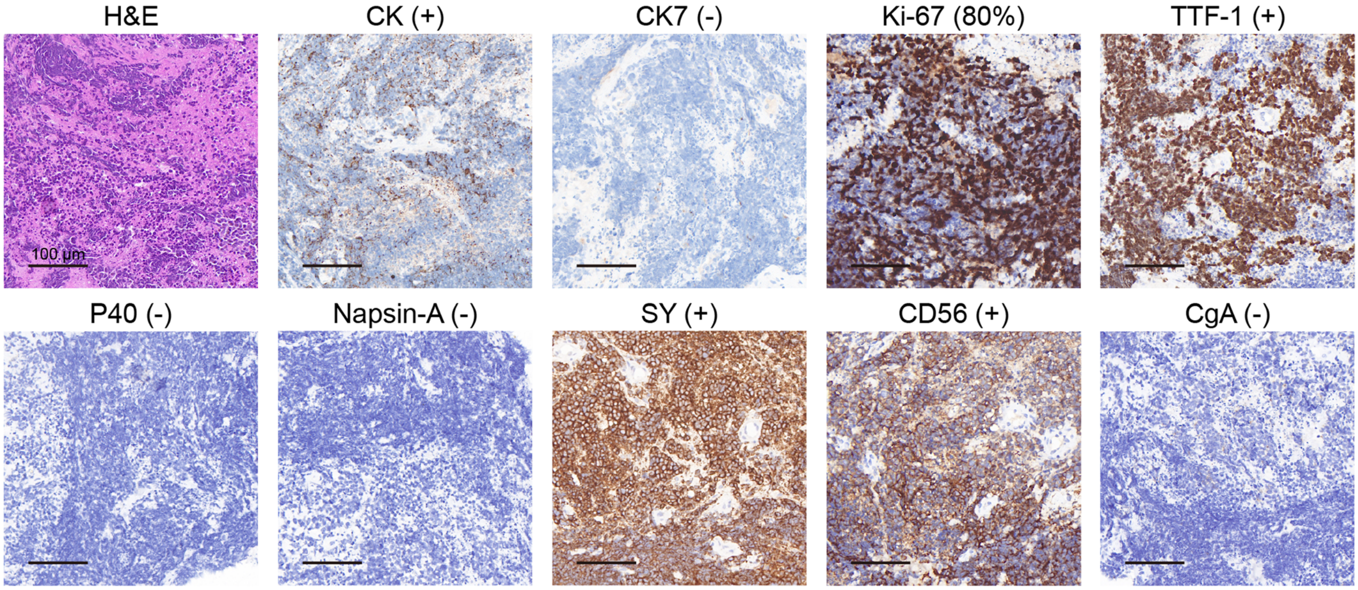

Figure EV5. Histopathological examination of the biopsied right upper lung mass.

The tested biomarkers indicated characteristics of small-cell lung cancer. "+" means positive expression, and "-" means negative expression. Scale bar: 100 μm.
